# Molecular dynamics reveals how calcium drives hetero- versus homodimerization of type I collagen

**DOI:** 10.1016/j.bpj.2026.01.033

**Published:** 2026-01-20

**Authors:** Emily J. Johnson, Shangze Xu, João V. de Souza, Anthony Evans, Agnieszka K. Bronowska, Elizabeth G. Canty-Laird

**Affiliations:** 1 Department of Musculoskeletal and Ageing Science, Institute of Life Course and Medical Sciences, University of Liverpool, William Henry Duncan Building, 6 West Derby Street, Liverpool L7 8TX, UK; 2 Computational Biology Facility, LIV-SRF, MerseyBio, University of Liverpool, Crown Street, Liverpool L69 7ZB, UK; 3 Institute of Systems, Molecular and Integrative Biology, University of Liverpool, Liverpool L69 7ZB, UK; 4 Chemistry-School of Natural and Environmental Sciences, Newcastle University, Newcastle Upon Tyne NE1 7RU, UK; 5 Newcastle University Centre for Cancer, Newcastle University, Newcastle Upon Tyne NE1 7RU, UK

## Abstract

Type I collagen is the main structural protein of vertebrates and forms molecular trimers from the *COL1A1* and *COL1A2* gene products, proα1(I) and proα2(I), during biosynthesis. Calcium ions are required for trimers to form. The amino acid sequence of the C-propeptide of collagen, which is removed before collagen fibril formation, initially drives heterotrimerization. Abnormal homotrimeric type I collagen is associated with age-related diseases including cancer, fibrosis, and musculoskeletal and cardiovascular conditions, but the circumstances under which the homotrimer may form are poorly understood. Here, we used molecular dynamics simulations of the C-propeptide protein structure to show that inter- and intrachain hydrogen bonding is affected by loss of calcium and that this leads chains to become destabilized, particularly at the interfaces of each chain. Loss of calcium resulted in increased distances between the cysteine residues that form interchain disulfide bonds, preventing the formation of these bonds. Pulling simulations and modeling of calcium dissociation from monomers showed that calcium ions were more strongly bound to the α1(I) than the α2(I) chain. However, enhanced sampling methods implied the α2(I) chain has a higher trimer affinity than a third α1(I) chain in the presence of structural calcium. To quantify assembly thermodynamics, we computed relative binding free energies by alchemical thermodynamic integration, demonstrating that α2(I)-specific residues at the interchain interface conferred a measurable thermodynamic advantage to trimer formation in the presence of calcium. Hence, although heterotrimerization is normally favored, in reduced calcium conditions the homotrimer can form by sequestering available calcium to the α1(I) chains. This study provides a molecular explanation for a calcium-based mechanism driving heterotrimerization versus homotrimerization of type I collagen.

## Significance

Type I collagen is the most abundant protein in the human body. Abnormal homotrimers contain three α1(I) chains, compared with normal heterotrimers with two α1(I) and, one α2(I). The homotrimers are implicated in cancer, fibrosis, and musculoskeletal and cardiovascular disease. Using molecular dynamics simulations and alchemical thermodynamic integration, we reveal how calcium concentration regulates trimer composition: heterotrimers are predicted to form preferentially when calcium is abundant (9.5-fold preference), but disruptions in endoplasmic reticulum calcium homeostasis—common in aging and disease—favor homotrimers because α1(I) chains bind calcium more strongly. This calcium-dependent mechanism could explain why homotrimers appear in pathological conditions and could provide molecular insights into collagen assembly disorders.

## Introduction

Type I collagen is normally a heterotrimer composed of two α1(I) chains and one α2(I) chain, derived from the *COL1A1* and *COL1A2* genes respectively. N- and C-terminal globular propeptide domains flank a 300-nm-long right-handed triple-helical domain that supercoils around a central axis with a pitch ranging from 10/3 to 7/2 depending on proline content (1). The helical region has a repeating Gly-X-Y amino acid structure, where X and Y are often proline and hydroxyproline, respectively. The lack of a side chain on glycine allows the bulky side chains of the other amino acids to occupy the outer positions, enabling tight packing (2,3). The C- and N-terminal propeptides confer solubility to the chains, preventing premature aggregation. The C-propeptide guides the trimerization process, as chain selection and alignment begins with C-propeptide trimerization, after which folding of the triple helix occurs from the C to N end (4). The propeptides are removed to facilitate assembly of trimeric type I collagen molecules into fibrils.

An abnormal homotrimeric form composed of three α1(I) chains has been reported in adult skin and embryonic tissues (5,6). However, the homotrimeric form is also associated with diseases such as cancer, osteoarthritis, osteoporosis, fibrosis, and Ehlers-Danlos syndrome (7,8). Molecular dynamics (MD) simulations of a 57-amino-acid region of the >1000-amino-acid triple-helical region have shown the homotrimer to be softer and more flexible (9). The homotrimeric helix freely rotates and forms kinks in the Gly-X-Y domain, which is predicted to lead to greater lateral distances between homotrimeric molecules in fibrils, consistent with experimental findings (10). Altered packing may be responsible for reported differences in intermolecular collagen cross-linking in the osteogenesis imperfecta murine (oim) mouse model (11,12,13), although the homotrimeric collagen is itself not responsible for bone fragility (14). Type I collagen homotrimer is resistant to proteolysis compared with the heterotrimer, and degradation by matrix metalloproteinase-1 (MMP-1) is approximately 10 times slower (15). The homotrimer however appears more sensitive to degradation under mechanical strain, while the heterotrimer is less sensitive (16,17). As the two trimeric forms of type I collagen demonstrate such different biophysical, dynamic, and structural properties, their physiological roles presumably differ, and synthesis needs to be tightly controlled to ensure the correct type of type I collagen is being produced.

The C-propeptide drives fibrillar collagen trimerization and determines trimer chain composition. In type III collagen, the chain recognition sequence coordinates trimerization and ensures that only α1(III) homotrimers form (18,19). This chain recognition mechanism does not however occur in type I collagen, where interchain interactions occur at key residues that form salt bridges (7), and trimer composition is partially governed by a network of disulfide bond-forming cysteines in the C-propeptide (20). The α1(I) chain C-propeptide contains eight cysteine residues (Cys 1–8), two of which participate in interchain disulfide bonding: C2 and C3. The α2(I) chain C-propeptide lacks the C2 residue and can only form one interchain disulfide bond. This ensures that only heterotrimers and α1(I) homotrimers can form and has been termed the “cysteine code.” Interchain disulfide bonding is however neither necessary nor sufficient for triple-helix formation, as the triple-helical domain of the α2(I) chain is unable to trimerize when coupled to a homotrimerizing α1(I) C-propeptide, but the α2(I) C-propeptide can trimerize and permit folding of a coupled α1(I) chain triple-helical domain (21). There is however a key role for calcium ions in mediating trimerization, as in the absence of available calcium in solution no heterotrimers or homotrimers can form (20). The α1(I) and α2(I) C-propeptides contain a conserved calcium-binding loop coordinating a structural calcium ion that sits at subunit interfaces in the C-propeptide trimer. Indeed, a *COL1A1* mutation substituting a calcium-binding residue in the C-propeptide of the α1(I) chain prevents trimerization and results in perinatal lethality (20,22).

In the present study, equilibrium MD simulations along with enhanced sampling techniques and free energy calculations were used to study type I collagen C-propeptide stability, with and without the structural calcium bound, to investigate how the structural calcium guides trimerization and how calcium homeostasis might play a role in homotrimer production.

## Materials and Methods

### Generating homology models

Homology models of the C-propeptide of heterotrimeric type I collagen were generated using the modified structure of the homotrimeric C-propeptide (PDB: 5K31) as a template (7). Mutations were reverted to the canonical sequence in PyMOL (“The PyMOL Molecular Graphics System,” Version 2, Schrödinger, New York, USA) and incomplete side chains rebuilt in SWISS-PDB Viewer. Glycerol and an excess chloride ion were also stripped. SWISS-MODEL (23) was used to create the homology model for the C-propeptide of heterotrimeric type I collagen. To create apo (Ca^2+^ depleted) versions of the proteins, the remaining calcium ions were also stripped. All trimers were simulated with the interchain disulfide bonds reduced. Monomers were created by extracting an α1(I) chain or the corresponding α2(I) chain.

### Equilibrium MD simulation protocol

GROMACS 2019.3 (24,25) was used to run all simulations of type I collagen trimers and monomers in solution. The AMBER99SB-ILDN force field (26) was used to describe the system topology. Three replicates were carried out. Hydrogens were replaced with virtual sites to allow for a longer time step of 5 fs and sampling of longer timescales (27). A fourth replicate with explicit hydrogens was also carried out for examination of hydrogen bonding patterns.

Standard simulation setups and protocols associated with the AMBER99SB-ILDN force field were used to carry out the MD calculations: electrostatic interactions were calculated using the particle-mesh Ewald method, and short-range nonbonded interactions were cut off at 1.0 nm (28,29). Verlet neighbor search was used with an update interval of 20 fs (30). The LINCS algorithm was used to constrain all bonds during the simulations (31).

TIP3P water model was used to solvate the system. The systems were neutralized using potassium ions, with additional potassium and chloride ions added to simulate physiological salt concentrations in the ER. Energy minimization was carried out using the steepest descent algorithm and terminated after 50,000 steps or when the maximum force was reduced to 1000 kJ mol^−1^ nm^−2^. Equilibration was carried out in two steps; the first step was a constant volume (NVT) ensemble where the protein and aqueous phase (water plus ions) were coupled to separate temperature baths at 310 K using the modified Berendsen thermostat (V-rescale). The second step was a constant pressure (NPT) equilibration with the Parrinello-Rahman barostat to maintain the pressure isotropically at 1.0 bar. Production runs were carried out in the NPT ensemble for 1000 ns (resulting in 16,000 ns of trajectory data in total).

Proteins were made whole and jumps and periodic boundary conditions were removed; then, translational and rotational movements were also removed. Analyses (root mean-square deviation (RMSD), root mean-square fluctuation (RMSF), radius of gyration, interchain distances) used standard GROMACS tools; trajectories were visualized in VMD (32) and PyMOL.

### Time series regression of MD data and further analysis

To compare differences in MD metrics between trimer types, accounting for the serial nature of the data, a Bayesian first-order linear autoregression (AR(1)) model was fitted using the *brms* v2.23.0 R package (33), utilizing the *Stan* probabilistic programming language (34). AR(1) terms were modeled for each replicate time series separately, with trimer types modeled as fixed effects. Weakly informative priors were utilized: β coefficients of trimer type effects were fitted with normal distribution priors with mean = 0 and standard deviation (SD) = 0.2, the AR(1) coefficient priors were specified as normal(mean = 0, SD = 0.3), the model intercept priors were specified as half-normal(mean = 0, SD = 1) with lower bound 0, and the model residual standard deviation priors were specified as exponential(rate = 1). Prior predictive checks revealed these priors imposed no effect of time and trimer type a priori, allowing any effects to be estimated primarily from the data while ensuring model predictions predominantly fell among a realistic range (Fig. S1).

Eight Monte Carlo Markov chains (MCMC) were used to fit the model posterior distribution, each with 1000 warmup and 1000 sampling iterations to yield 8000 draws per parameter. Model convergence was confirmed by ensuring R-hat statistics for all parameters were close to 1 as well as examining MCMC trace plots. Posterior predictive checks were performed using the *marginaleffects* v0.30.0 R package (35), to confirm that model predictions of expected metric values (means) were consistent with the data (Fig. S2). Counterfactual contrasts of expected value predictions were performed using the *tidybayes* v3.0.7 R package (36) for each trimer type comparison.

In addition to the above analysis, time-averaged structural properties were determined using a block-error approach, and block sizes were optimized using standard error convergence. Summary figures were produced using *ggplot2* (37) using the R programming statistical environment (version 4.3.2, R Core Team, 2023).

### Enhanced sampling protocols

Enhanced sampling methods were applied to type I collagen C-propeptide monomers and trimers to characterize chain-chain and ion-protein interactions. τRAMD (38), steered molecular dynamics (SMD), and umbrella sampling (39) were employed sequentially to explore dissociation pathways and quantify interaction free energies.

For trimeric systems, to investigate the affinity of an α1(I) or α2(I) chain for its neighboring two chains, τRAMD was first used to sample unbinding directions by pulling a mobile chain away from two stationary chains along randomized vectors. Twenty-five τRAMD trajectories were performed for each replicate following the standard τRAMD protocol.

Representative τRAMD trajectories were used to define the collective variable for subsequent SMD and umbrella sampling. Trimers were solvated, neutralized, and equilibrated as in the equilibrium simulations and rotated such that the mobile chain was orientated along the unbinding pathway. Backbone restraints were applied to the two stationary chains. A spring constant of 1500 kJ mol^−1^ nm^−2^ and a pull rate of 0.05 nm ps^−1^ were used, with 5 center-of-mass (COM) pulling simulations carried out per system. Average force-time and rupture-force profiles were obtained from these trajectories.

Snapshots spaced at 0.1–0.2 nm along the COM separation coordinate (to a maximum distance of ∼12 nm) were selected as starting structures for umbrella sampling. Due to computational resource limitations, umbrella sampling was carried out for one representative replicate per trimer system. Approximately 50 windows were simulated for 10 ns each, and the weighted histogram analysis method (WHAM) was used to construct the potential of mean force (PMF) (40). Statistical uncertainties in the PMFs were estimated using bootstrap resampling of the histograms. Bootstrapping was performed with 100 resamples to provide 95% confidence intervals for the free energy profiles (41).

To investigate calcium binding affinity for single α1(I) and α2(I) chains, the uncoupling of the structural calcium ion from the binding loop was analyzed using the same techniques. End-state coordinates from 10-ns production MD runs (three replicates) served as starting points (*n* = 3). Twenty-five τRAMD trajectories were performed per replicate using a 500-kJ mol^−1^ nm^−2^ pulling force applied to the ion in random directions until a 5-nm displacement from the loop was achieved. The relative residence time (τ_comp) was defined as the simulation time required for 50% of trajectories to exhibit complete dissociation. Results were analyzed using a freely available script (https://github.com/DKokh/tauRAMD), examining results for normality by visualizing the distributions and employing a Kolmogorov-Smirnov test.

For SMD of monomers, the calcium-binding loop backbone was position-restrained while side chains remained flexible. The calcium ion was pulled along the z-axis using a spring constant of 500 kJ mol^−1^ nm^−2^ and a pull rate of 0.005 nm ps^−1^. Snapshots were extracted every ∼0.2 nm up to a final COM distance of 5 nm, resulting in ∼30 umbrella sampling windows. Umbrella sampling was again performed for one representative replicate per monomer type, with each window simulated for 10 ns. WHAM was used to obtain the corresponding free energy profiles, with the same bootstrapping protocol as above.

### Alchemical relative binding free energy calculations

We computed relative binding free energies for heterotrimer formation using alchemical thermodynamic integration (TI) (42) via the thermodynamic cycle approach. Two interface mutations characteristic of the α2 chain (ASN-65 to THR and LEU-66 to MET) were evaluated individually in both complex (intact trimer) and solvent (monomer) routes for both apo- and holo-conditions.

Mutations were implemented using the pmx package (43) with the amber99sb-ildn-mut^∗^ force field and TIP3P water (44). Hybrid topologies were generated with mutate.py and generate_hybrid_topology.py and then processed with GROMACS (2023.2). Systems were energy minimized (50,000 steps, convergence at F_max <1000 kJ mol^−1^ nm^−1^) and then equilibrated for 500 ps each in NVT (300 K, V-rescale thermostat) and NPT (1 bar, Parrinello-Rahman) ensembles. For holo simulations, to ensure comparable reference frames across mutations were investigated, harmonic distance restraints (1000 kJ mol^−1^ nm^−2^) maintained Ca^2+^ coordination to three conserved heavy atoms within 4 Å.

**Figure 1:**
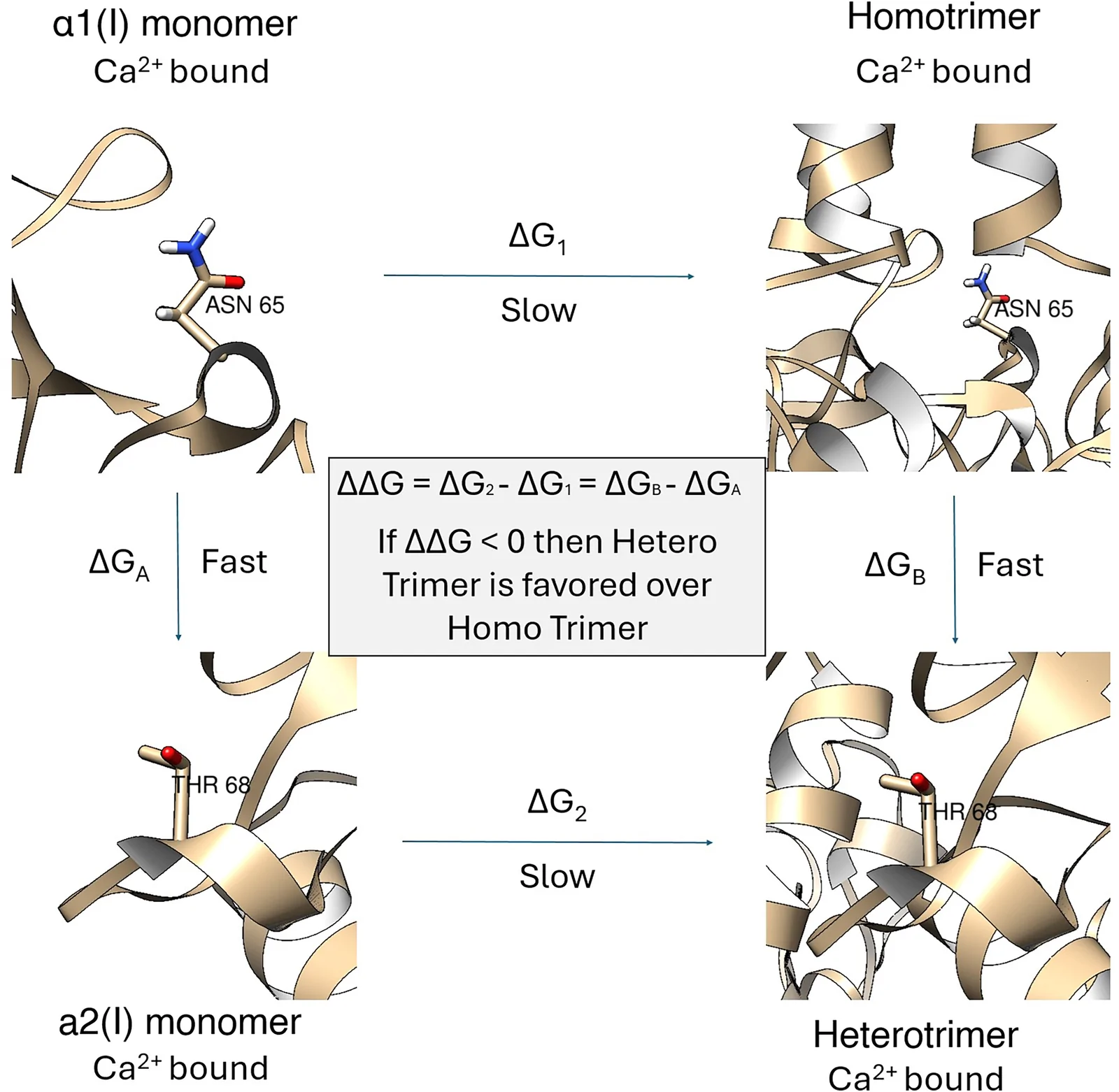
Thermodynamic cycle for relative binding free energy calculations. Top row: homotrimer and α1(I) chain; bottom row: heterotrimer and α2(I) chain. The left column is monomer, and the right column is trimer. Horizontal arrows ΔG_1_ and ΔG_2_ represent the assembly free energy of monomer to trimer (using slow TI); vertical arrows ΔGA and ΔGB represent alchemical mutations in the monomeric or trimer context (homotrimer to heterotrimer). Closure of the circuit gives the relative binding free energy (RBFE).

All alchemical runs were performed as a single continuous λ with GROMACS (free_energy = yes). The MD time step was 2 fs, and each trajectory was propagated for 1 × 108 steps, giving a total simulation time of 200 ns. The coupling parameter started from the homo state at λ_0_ = 0 and was increased linearly by Δλ = 1 × 10^−8^ per MD step (init_lambda = 0, delta_lambda = 0.00000001), reaching λ = 1 at the end of the run. Soft-core nonbonded potentials were used with α = 0.5, σ = 0.3 nm, and power = 1. Resulting dH/dayλ time series were numerically integrated over λ to obtain ΔG. Relative binding free energies were calculated as ΔΔG_bind = ΔΔG_complex − ΔΔG_monomer, with calcium dependence estimated as ΔΔΔG_Ca = ΔΔG_bind(holo) − ΔΔG_bind(apo) (Fig. 1).

### Interchain interaction enthalpies

Procollagen heterotrimer (two α1(I) chains and one α2(I) chain) was modeled using the crystal structure of fibrillar procollagen type I C-propeptide homotrimer (PDB: 5K31) and AlphaFold3 (45) model of α2(I) chains. The missing calcium cation was fitted, and both trimers were energy minimized. Interchain interaction enthalpy calculations for hetero- and homotrimers were performed using parameters derived from AMBER parm99 classical molecular mechanical force fields and a GB/SA implicit solvation model. All calculations were performed using INTAA webserver (46).

## Results

### The C-propeptide shows increased RMSD but decreased radius of gyration over 1000 ns of simulation

**Figure 2:**
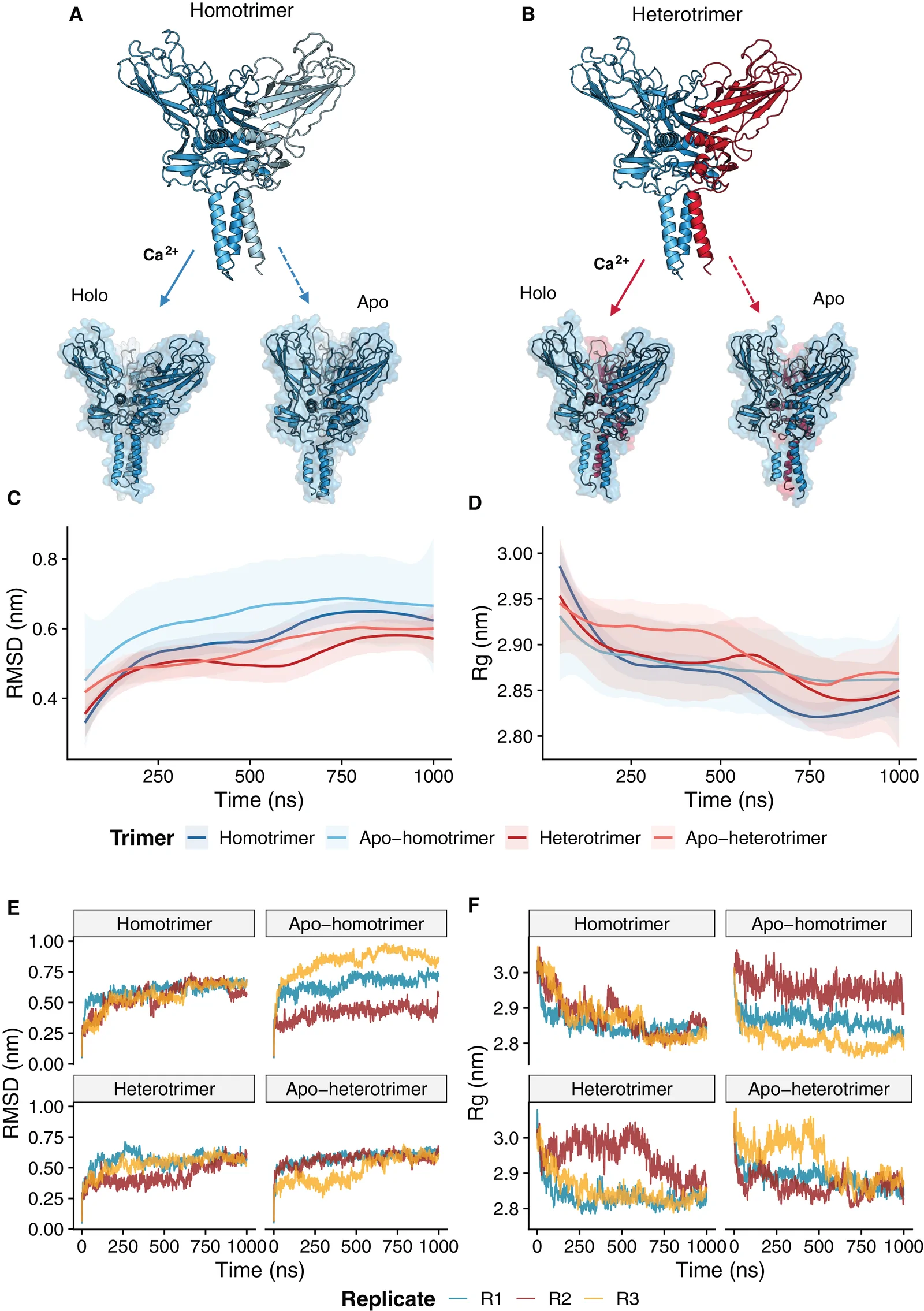
Molecular dynamics simulations of apo and holo version of the type I collagen C-propeptide homotrimers and heterotrimers. (*A* and *B*) Starting structures of the C-propeptide homotrimer (*A*) and heterotrimer (*B*) compared with the holo- and apo-trimers after 1000 ns of simulation. The α2(I) chain is shown in red for the heterotrimer structures, and the corresponding α1(I) chain in the homotrimer is shown in a lighter blue. Images are derived from one of three repeat simulations. (*C* and *D*) Time evolution of the root mean-square deviation (RMSD) (*C*) and radius of gyration (Rg) (*D*) over 1000 ns. Shaded regions represent ±1 standard error from block-averaged data across three replicate simulations, with smoothed mean trajectories overlaid. (*E* and *F*) Individual replicate trajectories for RMSD (*E*) and Rg (*F*) for each trimer system (homotrimer, apo-homotrimer, heterotrimer, apo-heterotrimer). Each replicate (termed “R1,” “R2,” and “R3”) is shown as a separate trace in blue, red, and yellow.

A model for the type I collagen heterotrimeric C-propeptide was generated by homology modeling using the crystal structure of the homotrimeric form as a template (Fig. S3). MD simulations were performed for 1 μs for both the homotrimer (Fig. 2 *A*) and heterotrimer (Fig. 2 *B*), in either the holo or apo states. In all simulations, the backbone RMSD increased over time (Fig. 2 *C*), whereas the radius of gyration (Rg) showed a gradual decrease (Fig. 2 *D*), indicating compaction of the trimeric assemblies during equilibration. Individual replicates for RMSD (Fig. 2 *E*) and Rg are shown (Fig. 2 *F*).

Overall, the RMSD did not consistently differ between trimer types (Table 1; Fig. S4A). Holo forms were generally more stable than corresponding apo forms, although variability was high, particularly for the apo-homotrimer. Conversely, the homotrimer maintained a slightly tighter fold and appeared to be more compact than the heterotrimer, based on lower Rg values. The holo forms showed marginally lower Rg than the corresponding apo forms, perhaps suggesting a loss of compactness upon calcium depletion. However, for both RMSD and Rg, the sampling error was large, making it difficult to infer any structure-wide differences (Table S1; Figs. S4 *B* and S5). As such, we next examined local residue-level interactions and bonding patterns at the trimer interfaces.

**Table 1:** Intertrimer Type Differences in Mean RMSD Model Predictions

Contrast	Difference in Mean RMSD Predictions (Median, 95% CrI)
Homotrimer - Apo-homotrimer	−0.0027 (−0.027–0.022)
Heterotrimer - Apo-heterotrimer	−0.0018 (−0.027–0.024)
Homotrimer - Heterotrimer	0.00022 (−0.026–0.026)
Apo-homotrimer - Apo-heterotrimer	0.0014 (−0.024–0.026)

For each contrast, mean RMSD predictions were estimated for the stated trimer types using all draws from the model posterior. The differences in per-draw predictions were calculated and summarized here as median and 95% credible (quantile) intervals.

### Hydrogen bonding is affected by the loss of calcium

**Figure 3:**
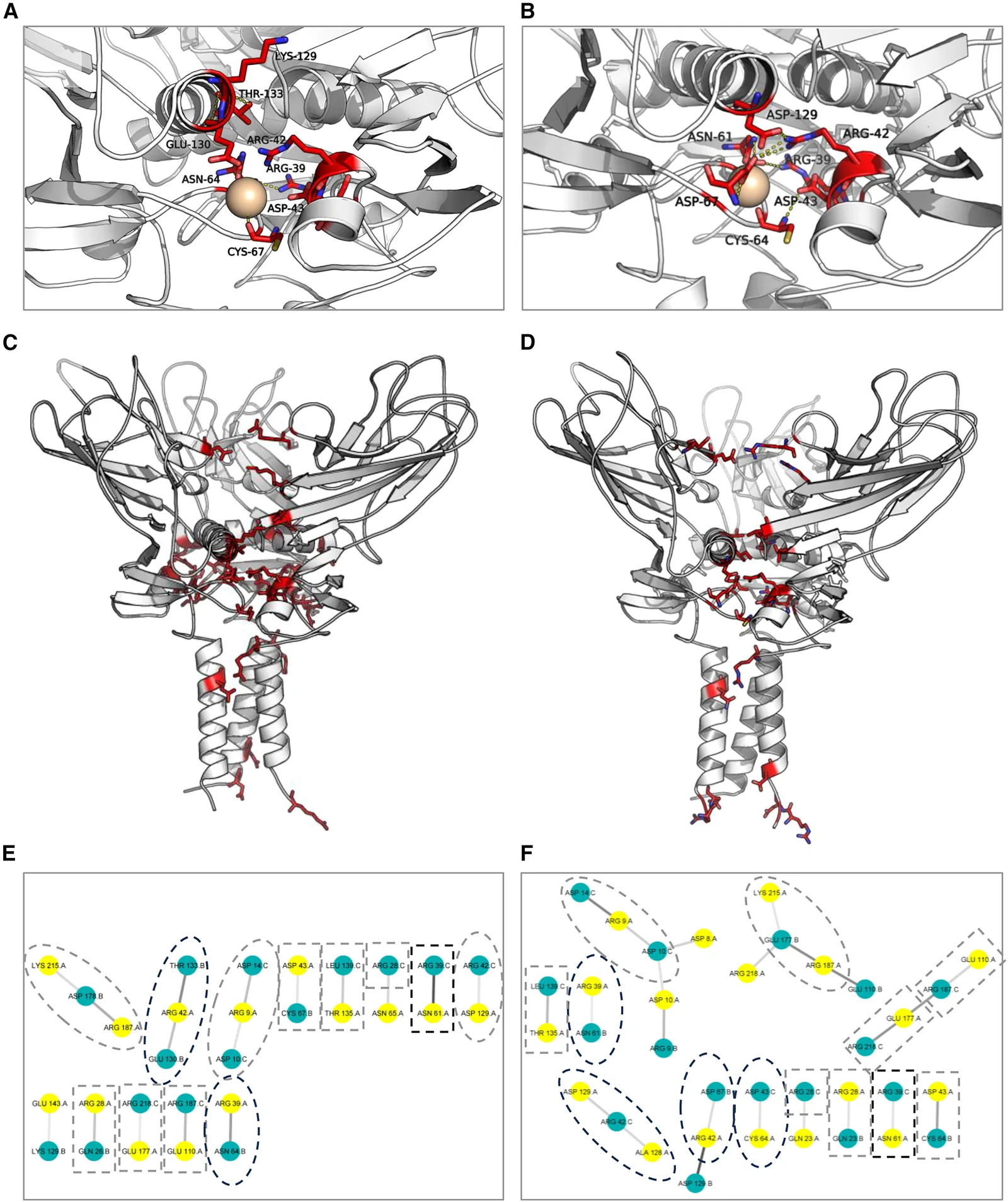
Hydrogen bonding networks in C-propeptide heterotrimers and homotrimers revealed by StructureViz analysis. (*A*–*D*) Trimer cartoons with residues that participate in interchain hydrogen bonds shown in red and as “stick” representations. (*A* and *B*) Hydrogen bonds at the chain interface for the heterotrimer (*A*) and homotrimer (*B*). (*C* and *D*) Interchain hydrogen bonds for the whole structure for the heterotrimer (*C*) and homotrimer (*D*). The calcium ion is shown as a wheat-colored sphere. The N-terminal regions demonstrate increased flexibility due to the lack of Gly-X-Y domain. (*E* and *F*) Hydrogen bonding network for the α1(I) chain A from explicit hydrogen MD simulations for the heterotrimer (*E*) and homotrimer (*F*). Only one replicate was carried out with explicit hydrogens, with a time step of 2 fs. α1(I) chain A is shown in yellow; chain B, i.e., α1(I) in the homotrimer and α2(I) in the heterotrimer, is shown in teal; α1(I) chain C is also shown in teal. The edge weight corresponds to how conserved the bond was throughout the simulation. The darker edges represent bonds that were present throughout most of the simulation; the lighter ones were more transient bonds. Black circles denote bonds referred to in the text. Gray circles denote bonds that are conserved, or similar, between the heterotrimer and homotrimer.

To examine how calcium influences the hydrogen bonding network, inter- and intrachain hydrogen bonds were identified and analyzed using the Cytoscape–Chimera StructureViz package (Figs. 3 and S6; Table S2). In the homotrimer, residues CYS-64 (C3), ASP- 67 and ASN-61 within the calcium-binding loop formed hydrogen bonds with ASP-43, ARG-42, and ARG-39, respectively, on the neighboring chain. This region corresponds to the α-helix containing the partner cysteine (C2) involved in C2–C3 binding. These interactions were conserved between the α1(I) and α2(I) chains.

The α1(I) alpha-helical central region contains an ASP-129 residue that formed a salt bridge with ARG-42 on the neighboring chain as previously described (7). ALA-128 could also form a hydrogen bond with ARG-42, although not as frequently. In the heterotrimer, the α2(I) chain GLU-130 residue formed a salt bridge with ARG-42 on the neighboring chain instead.

These interchain interactions were stabilized by intrachain hydrogen bonds. In the α1(I) chains, the ARG-39–ASN-61 hydrogen bond was reinforced by additional intrachain interactions between ASN-61 and GLN-133, and between ARG-39 and both PRO-60 and GLN-62. The ARG-42 and ASP-67/ASP-129 salt bridges were stabilized by ARG-42 forming hydrogen bonds with LEU-246 and THR-142. ASP-43 also formed an intrachain salt bridge with ARG-39, which may act to stabilize the protein structure and overall interchain hydrogen bonding network, as ARG-39 is one of the residues that participates in binding at the interface. In the heterotrimer, the stabilizing intrachain hydrogen bonds for the α2(I) chain involved ARG-45–PHE-246, ASN-64–GLN-134, and ASP-70–GLN-134 interactions.

In addition to ASP-43 forming an interchain hydrogen bond with CYS-64 and intrachain salt bridge with ARG-39, it also forms an intrachain hydrogen bond with CYS-47 (i.e., it binds both partners in C2-C3 disulfide bond formation). It may be that this residue shuttles between forming hydrogen bonds with one cysteine or the other, coordinating their association during trimer assembly.

**Figure 4:**
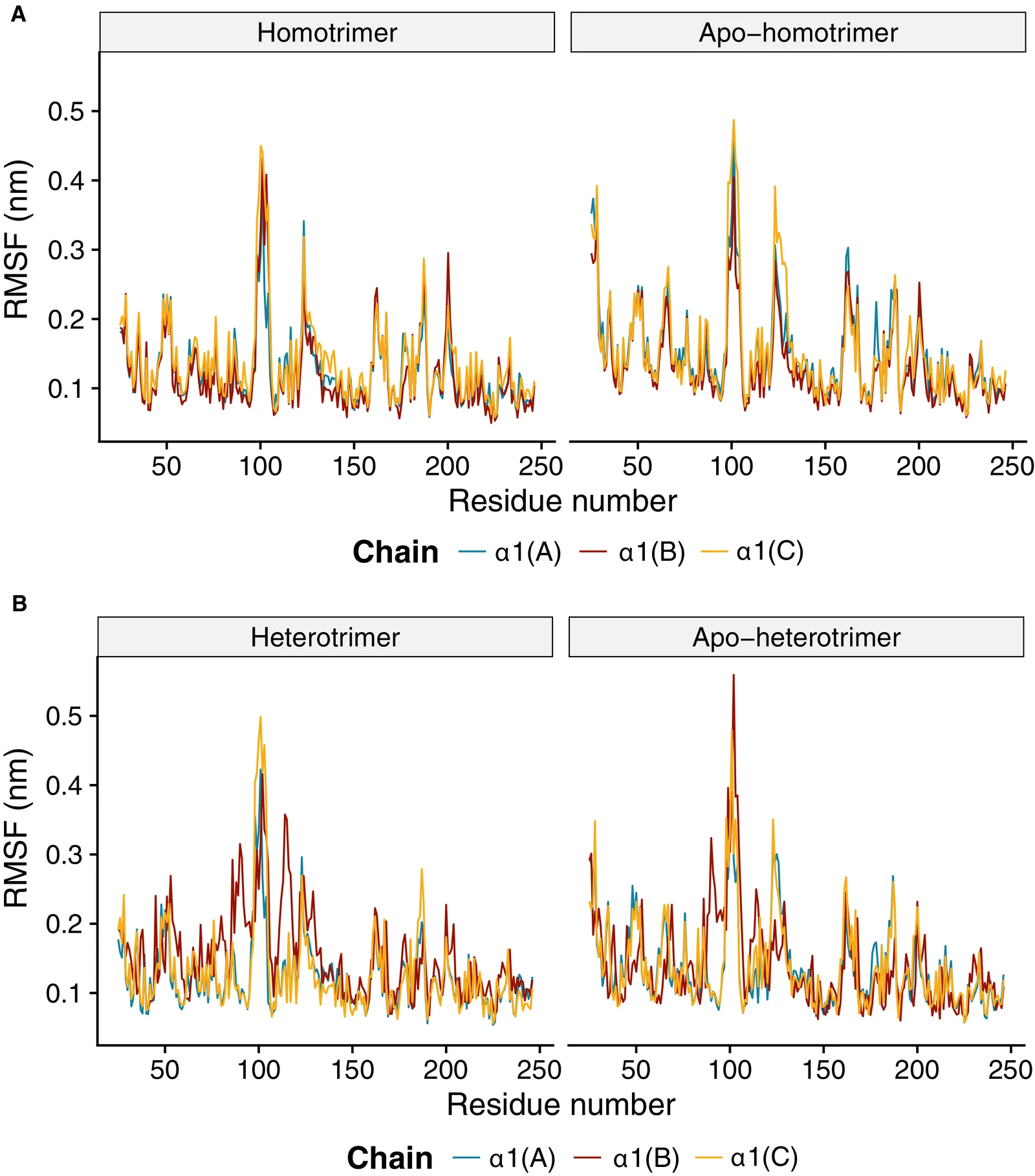
The RMSF of the backbone as a function of amino acids. (*A*) Holo-homotrimer and apo-heterotrimer. (*B*) Holo-heterotrimer and apo-heterotrimer. Each trimer has three chains: chains A, B, and C. In the homotrimer, all three chains are α1(I). In the heterotrimer chains A and C are α1(I) and chain B is α2(I). Each chain is colored separately per trimer: blue (chain A), red (chain B), and yellow (chain C).

To assess whether the hydrogen-bonding networks at the chain interfaces are affected by calcium loss, RMSF values were calculated per residue over each 1000-ns trajectory (Fig. S7) and averaged across the three replicates (Fig. 4).

**Figure 5:**
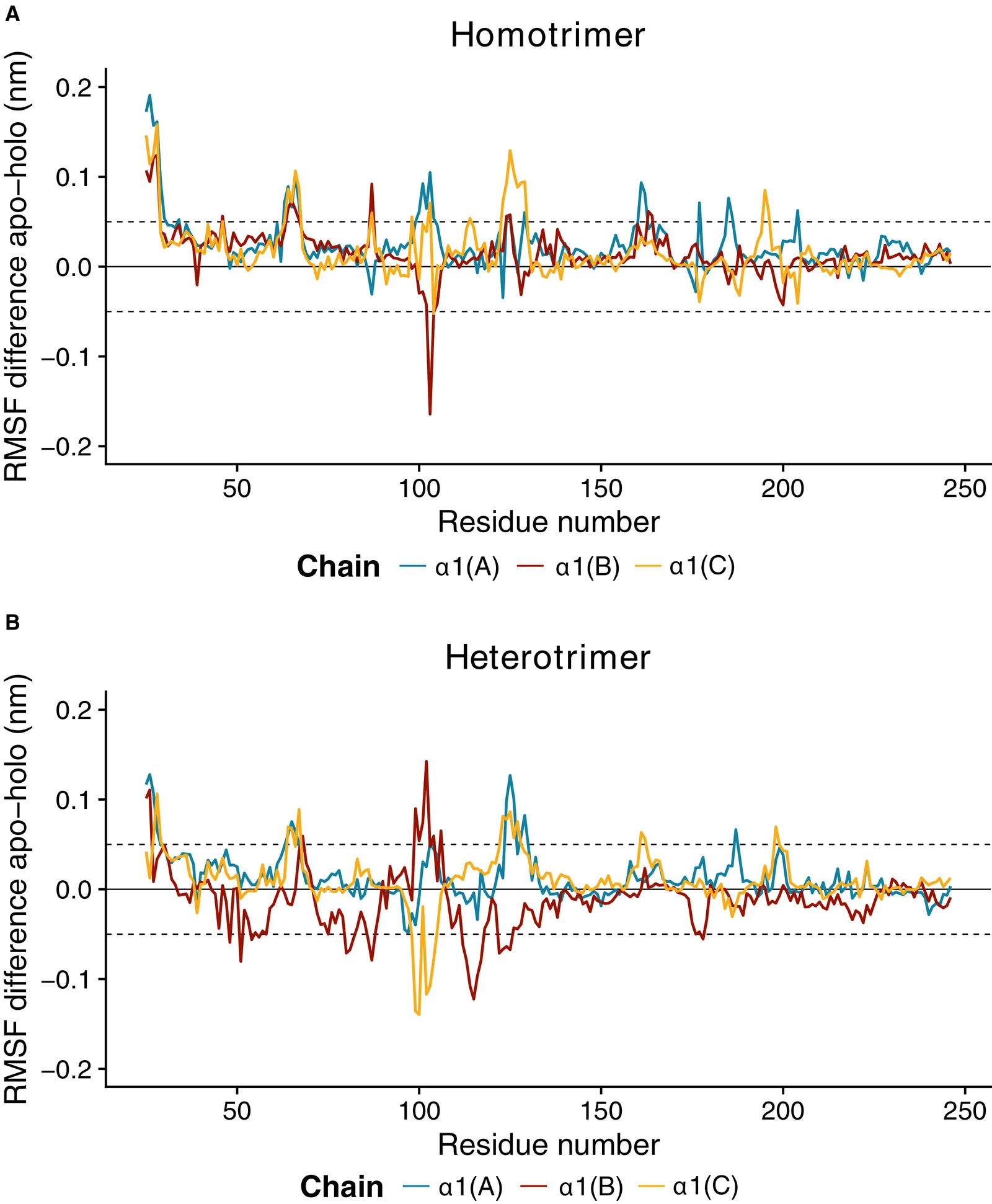
Per-residue RMSF differences between holo- and apo-trimers. The RMSF values for the apo-trimers were subtracted from the holo-trimers and the difference was then plotted per chain for the homotrimers (*A*) and heterotrimers (*B*).

To capture changes in dynamics that were directly due to calcium loss, RMSF differences between apo and holo states were calculated for both the heterotrimer and homotrimer (Fig. 5) (termed RMSF “hotspots”). Positive values indicated increased flexibility in the apo form relative to the holo form (destabilization); negative values indicated reduced flexibility in the apo form relative to the holo form (stabilization). This revealed an overall destabilization of the α1(I) chains of the heterotrimer and homotrimer without calcium, indicated by positive values.

**Figure 6:**
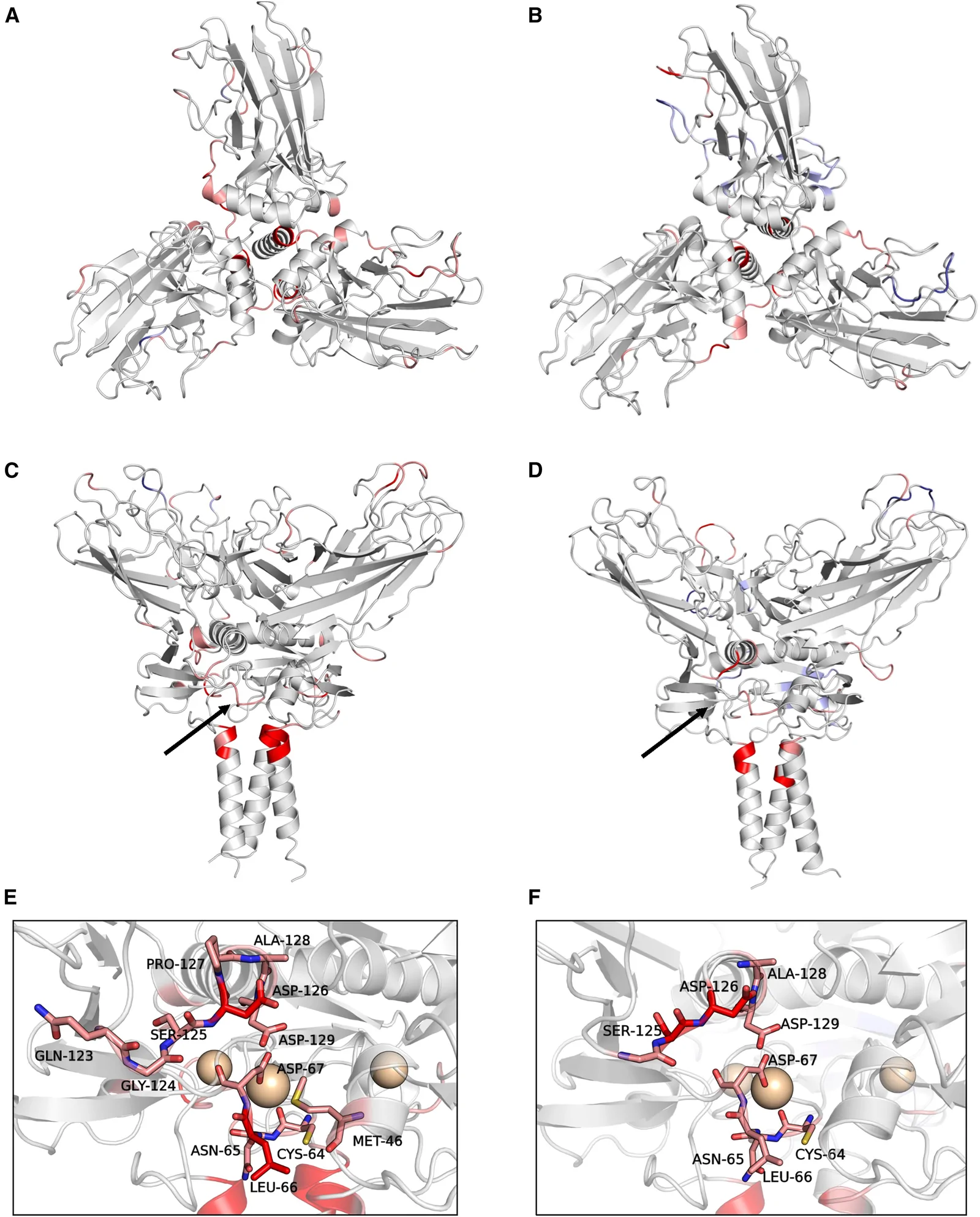
RMSF hotspots in the heterotrimer and homotrimer. (*A* and *B*) Top-down view of the homotrimer (*A*) and heterotrimer (*B*). (*C* and *D*) Side-on view of the homotrimer (*C*) and heterotrimer (*D*). Left chain is an α1(I) chain, and right chain is an α1(I) B chain in the homotrimer or an α2(I) B chain in the heterotrimer. (*E* and *F*) Zoomed view of calcium-binding region at the interface of the homotrimer (*E*) and heterotrimer (*F*). For all panels, RMSF hotspots (defined as residues with ΔRMSF values >0.05 or <−0.05) are colored in. Red: ΔRMSF >0.1; salmon: ΔRMSF 0.05–0.1; light blue: ΔRMSF −0.05 – 0.1; dark blue: ΔRMSF <−0.1. Calcium ions are shown as wheat-colored spheres.

To visualize the location of the RMSF hotspots, residues with ΔRMSF values >0.05 or <−0.05 were mapped onto the trimer structures (Fig. 6; Table S3). This confirmed that interface regions near the base of the trimers were most perturbed by removal of calcium ions from the structures. Many of the residues that were found to participate in interchain hydrogen bonds or salt bridges were among those destabilized. Interestingly, the exterior face of the α2(I) chain was stabilized in response to calcium depletion in the heterotrimer (Fig. S8). This could reflect the true interface no longer being favorable in the absence of bound ions. A notable stabilization trough was observed in the α1(I) B chain of the homotrimer (Fig. 5); however, this region was largely disordered and appeared to be driven by a single replicate (Fig. S7).

**Figure 7:**
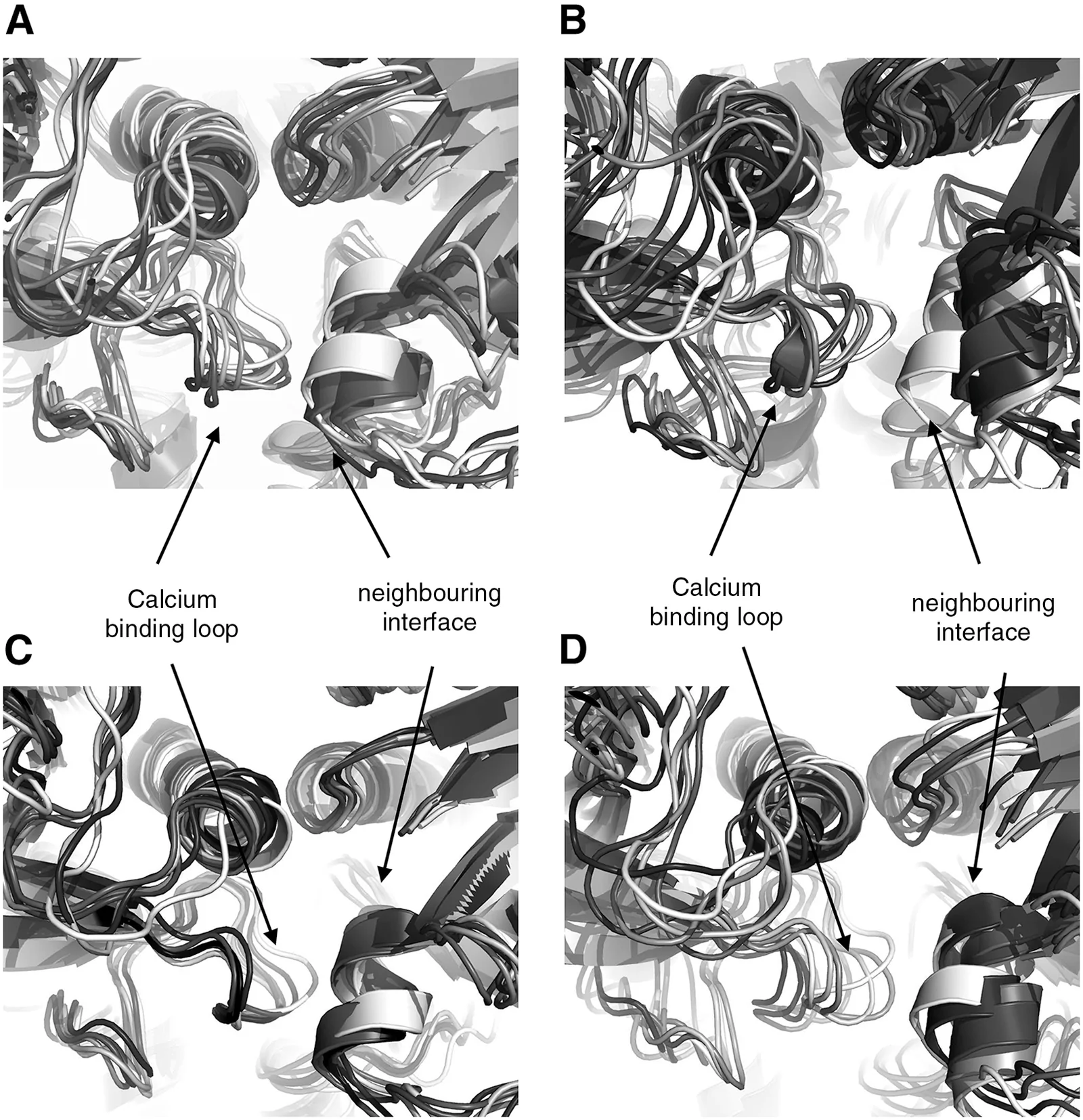
Time evolution of the calcium-binding region in homotrimers and heterotrimers. (*A*) Holo-homotrimer (*B*) Apo-homotrimer. (*C*) Holo-heterotrimer. (*D*) Apo-heterotrimer. Snapshots were taken every 200 ns from 0 to 1000 ns and overlaid. The time points are colored from the earliest time point in the lightest shade (*white*) to the latest time point in the darkest shade (*black*). Calcium ions are omitted from the holo structures for visual clarity.

To visualize the dynamic effects of calcium depletion over time, snapshots of the calcium binding region were taken every 200 ns, starting from 0 ns, for both the holo and apo systems, and then the structures were overlaid (Fig. 7). In both trimers, intrinsically disordered regions became more mobile upon calcium loss, but the calcium-binding loop was most obviously affected. In the holoproteins (Figs 7, A and C), the calcium-binding loops maintained a consistent orientation, remaining close to the neighboring chain. In contrast, in the apo systems the loop exhibited increased conformational flexibility, moving inward and away from the interface. The apo-homotrimer also developed a minor fold at later time points (Figs 7, B and D). The calcium-binding loop appeared more stable in the holo-heterotrimer than the holo-homotrimer.

Together, the StructureViz analysis, RMSF values, and trajectory visualization demonstrate a role for structural calcium in coordinating hydrogen bond and salt bridge formation at the interface between chains. In the absence of calcium, these interface regions display increased conformational flexibility, which is likely to impair trimer assembly. Notably, these interface loops also contain the disulfide-forming cysteines, which next prompted the investigation of calcium coordination and cysteine pairing.

### Calcium maintains sufficient proximity for interchain disulfide bonding

**Figure 8:**
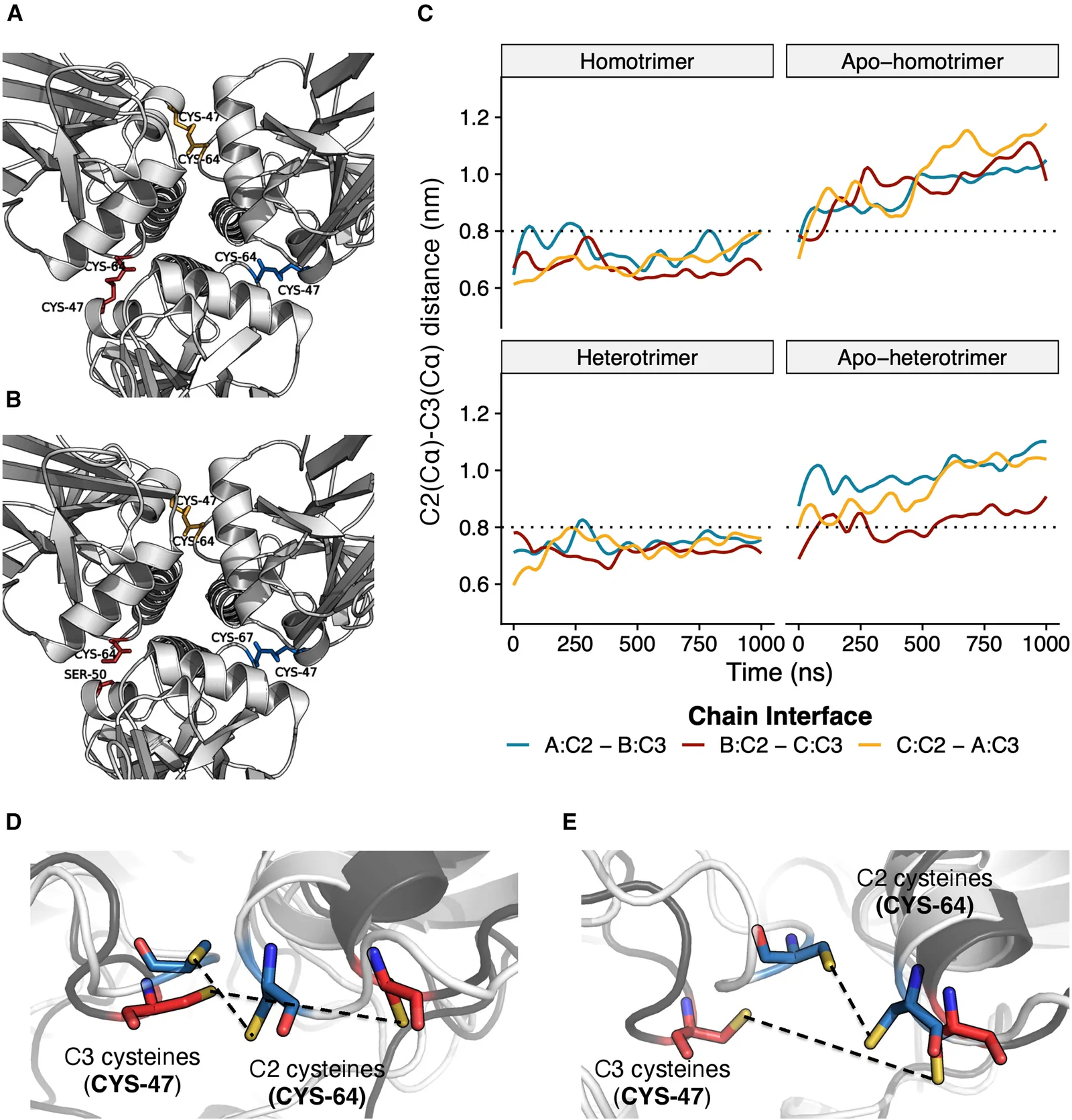
Interchain disulfide bonds and simulated interresidue distances for the C-propeptide heterotrimer and homotrimer. (*A* and *B*) Top-down view of the interchain disulfide bonding cysteines for the homotrimer (*A*) and heterotrimer (*B*). For the heterotrimer (*B*), the α2(I):α1(I) chain interface (chain B:chain C) (*red*) does not contain a disulfide bond, due to the Cys-Ser substitution in the α2(1) chain. The bonds are shown as sticks. The remainder of the chains are shown in light gray as cartoon representations. (*C*) Smoothed average distance between the Cα atoms of the interchain disulfide forming residues over the course of the three equilibrium simulation replicates. Each panel shows a different trimer, in order: the homotrimer, apo-homotrimer, heterotrimer, apo-heterotrimer. Each trimer has three chains: chains A, B, and C. In the homotrimer, all three chains are α1(I). In the heterotrimer, chains A and C are α1(I), and chain B is α2(I). The chain A-B interface C2-C3 distance is shown in blue, the chain B-C interface C2-C3 distance is shown in red, and the chain C-A interface C2-C3 distance is shown in yellow. In the heterotrimer the red interface is the distance between the equivalent SER-50 Cα and CYS-64 Cα (C3). (*D* and *E*) Zoomed-in view of the C2-C3 cysteines off the homotrimer (*D*) and heterotrimer (*E*) α1 chain A and α1 chain C (*yellow interface*). The cysteines are shown as stick representations. Blue: 0 ns; red: 1000 ns. Distances between the sulfhydryl groups are shown as dotted lines.

It is known that after the individual α-chains associate, they become irreversibly disulfide bonded by protein disulfide isomerase (PDI). The cysteine code ensures the proper assembly of homotrimers and heterotrimers and prevents α2(I) homotrimers from forming interchain disulfide bonds. In the absence of calcium, no trimers form; only monomers and short-lived dimers are present (20). The relevant cysteines and the α2(I) serine are visualized in Figs. 8, *A* and *B*.

To determine the impact loss of calcium has on covalent bond formation, the distance between the Cα atoms of C2 and C3 cysteines, and the α2(I) serine to C3 cysteine for the heterotrimer, at each chain interface was measured (Fig. 8 *C*). Disulfide bonds can form between cysteines at distances of 0.3–0.75 nm (47,48). After the disulfide bond has formed the linkage is typically about 2.04 Å (0.2 nm) in length. At distances greater than 0.8 nm, disulfide bonds are unlikely to be observed (47). For the holo-trimers, the distance between the C2 and C3 cysteines remained near constant through the equilibrium simulations, fluctuating between 0.6 and 0.8 nm (Figs. 8 *C*, S9, and S10), with only modest differences in mean distance observed between the holo-trimers at chain interfaces B and C (Fig. S11; Table S4). In contrast, for the apo-trimers, the C2-C3 distance increased over the course of the simulation, with average values generally greater than 0.9 nm (Fig. S10). Consistent with this observation, mean distances were greater for apo-trimers than their respective holo-trimers across all interfaces (Fig. S11; Table S4). Hence, simulations indicate that structural calcium in the trimers maintains a suitable distance between the C2 and C3 cysteines for covalent disulfide bonds to form.

### Pulling simulations and τRAMD demonstrate that calcium is more strongly bound to α1(I) than the α2(I) chain

**Figure 9:**
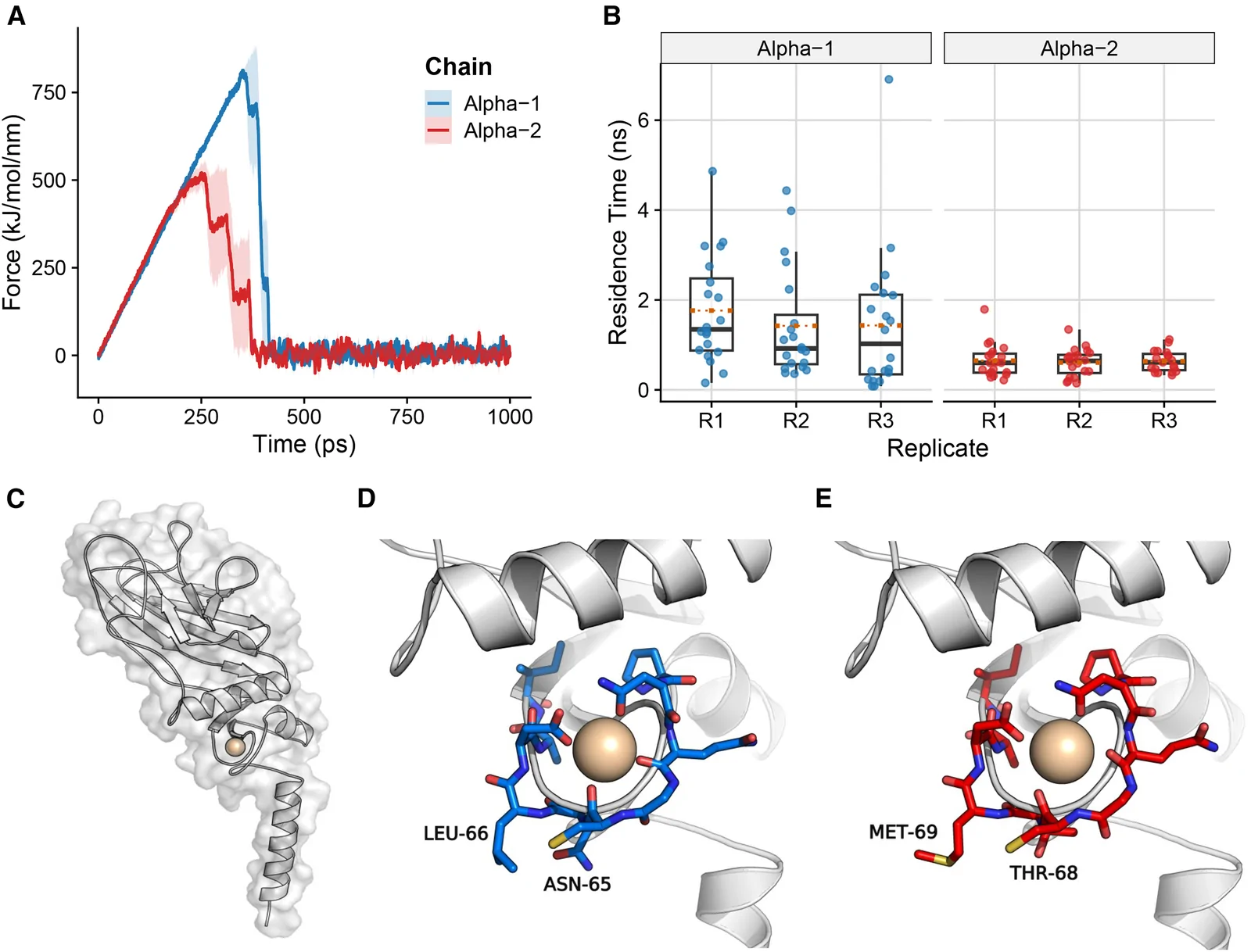
Steered molecular dynamics simulations and τRAMD simulations of calcium uncoupling from C-propeptide monomers. (*A*) Force-time curves derived using default Amber ff99SB-ILDN parameters. The darker lines are the average values over five replicates. The shaded areas are the error (standard deviation). (*B*) The residence times for three replicas for the α1(I) and α2(I) chain. Boxplots summarise the τRAMD results (25 independent trajectories per replicate). Boxes represent the interquartile range (IQR; 25th to 75th percentiles), whiskers extend to the most extreme values with 1.5 times the interquartile range. Individual points correspond to residence times from single τRAMD trajectories. The median value is shown as a black line and the mean value by an orange dotted line. (*C*) Surface representation of an α1(I) monomer, demonstrating that calcium is exposed to solvent. (*D* and *E*) Differences between the α1(I) (*D*) and α2(I) (*E*) calcium-binding loops. Calcium ions are shown as spheres.

The calcium ion is coordinated by three conserved residues in the calcium binding loop: ASP-59, ASN-61, and ASP-67 in the α1(I) chain and ASP-62, ASN-64 and ASP-70 in the α2(I) chain. The calcium binding loops share 83% sequence identity and vary by only two residues. ASN-65 and LEU-66 in the α1(I) chain are replaced by THR-68 and MET-69 in the α2(I) chain. To compare calcium binding strength, COM pulling simulations and τ-random accelerated molecular dynamics (τRAMD) were performed (Fig. 9).

After a short 10-ns equilibration, COM pulling simulations (SMD) were carried out to extract the calcium ion from its binding site (Fig. S12) (49). The resulting force-time curves for the dissociation of calcium from each chain showed an approximately twofold difference in the rupture force (F_max_) required to separate the calcium ion from its binding site in the α1(I) chain, compared with the α2(I) chain (Figs 9 *A* and S13). The force profiles demonstrated a three-step unbinding pattern, appearing to correspond to the sequential loss of the three coordinating residues.

Umbrella sampling was employed to compute the PMF along the unbinding coordinate (Fig. S14). Both α1(I) and α2(I) unbinding profiles featured a single intermediate (PL^∗^), consistent with a two-state release mechanism. The bootstrapped standard deviation of the PMF was narrow (<0.5 kcal mol^−1^) in the bound region and widened slightly upon ion release (Fig. S14 *A*). The α1(I) chain exhibited a deeper binding well (ΔG_min_ = – 4.99 kcal mol^−1^) than α2(I) (ΔG_min_ = – 3.52 kcal mol^−1^) (Table S5), suggesting a more stable bound state; however, there was only a modest change in Gibbs free energy, ΔG (50). The similar free energies suggested the α1(I) chain and α2(I) chains have comparable binding affinity for the calcium ion, in contrast with the SMD, which had higher rupture forces for α1(I). This could reflect the one-dimensional reaction coordinate being insufficient to capture all the barriers arising from coordination and local gating, and additionally, the histograms showed some limited sampling at the start of the reaction coordinate, despite the narrow windows (Fig. S14B).

τRAMD simulations were employed to cross-validate the pulling results and to quantify relative residence times (τ) of calcium in each chain (Figs 9 *B* and S15). Each trajectory was tested for distributional normality using the Kolmogorov-Smirnov (KS) test (Table 2; Figs. S15, *E* and *F*). Across replicates, calcium residence times were significantly longer in α1(I) (mean = 1.54 ± 0.22 ns) than in α2(I) (mean = 0.63 ± 0.06 ns), corresponding to a ∼2.4-fold difference (*p* = 0.014; Table 3). The slower dissociation kinetics in α1(I), coupled with the deeper PMF well and higher rupture forces, suggest that although both loops have similar overall thermodynamic affinity, calcium is mechanically and kinetically more tightly bound to α1(I).

**Table 2:** Residence Times, Relative k_off_ Values, and Kolmogorov-Smirnov Test Results Calculated from τRAMD Simulations

Chain	Replicate	Mean Relative Residence Time (ns)	Standard Deviation (ns)	KS Test
α1(I)	1	1.76	0.14	0.2
α1(I)	2	1.42	0.09	0.15
α1(I)	3	1.43	0.36	0.16
α2(I)	1	0.65	0.06	0.26
α2(I)	2	0.61	0.05	0.18
α2(I)	3	0.64	0.06	0.39

**Table 3:** Mean Residence Times and Relative k_off_ Values Calculated from τRAMD Simulations

Monomer	Mean Residence Time (ns)	Standard Deviation	Relative k_off_ (ns^−1^)
α1(I)	1.54	0.22	0.65
α2(I)	0.63	0.06	1.59

### Enhanced sampling methods suggest that the α2(I) chain has a higher trimer affinity than a third α1(I) chain in the presence of structural calcium

To investigate the energetic basis for preferential heterotrimer formation, a combination of SMD, umbrella sampling, interchain interaction enthalpy (INTAA) analysis, and thermodynamic integration calculations was employed. These complementary methods were used to evaluate both the mechanical and thermodynamic stability of homotrimeric and heterotrimeric type I collagen C-propeptide assemblies for both the holo and apo states.

**Figure 10:**
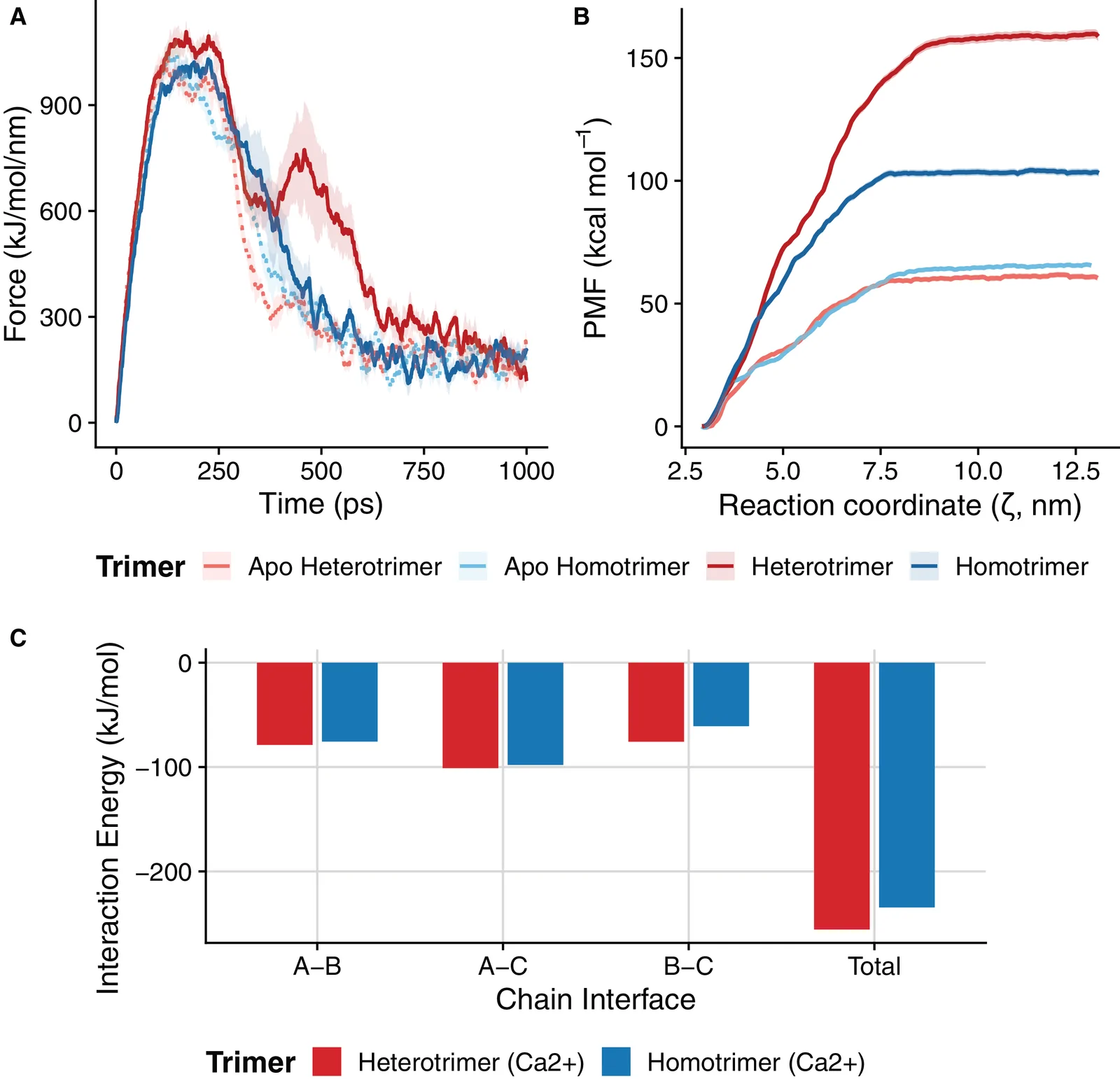
Steered molecular dynamics simulations of the α2(I) or corresponding α1(I) chain pulled from C-propeptide heterotrimers or homotrimers and interchain interaction enthalpies. (*A*) Force-time curves of type I collagen trimers with and without calcium bound (holo and apo). The darker lines are the average values over 5 replicates. The shaded areas are the error (standard deviation). (*B*) Potential of mean force (PMF) curves for: heterotrimer, apo-heterotrimer, homotrimer, and apo-homotrimer, obtained via weighted histogram analysis (WHAM). The PMF is shown as a solid line, and the shaded regions denote the bootstrapped ±1 SD uncertainties (100 resamples). (*C*) Summary of interchain interaction enthalpy (INTAA) calculations for each trimer at each interface.

The force-time profiles generated from SMD (Fig. S16) suggested that the holo-heterotrimer exhibited the highest rupture force, followed by the holo-homotrimer, whereas both apo systems were considerably weaker (Fig. 10 *A*). The heterotrimer displayed a biphasic unbinding process, corresponding to an initial rupture followed by a smaller secondary event that was mirrored in the number of interchain hydrogen bonds.

Umbrella sampling analyzed using the WHAM produced PMF profiles for chain dissociation (Fig. 10 *B*). Histogram overlap confirmed sufficient sampling (Fig. S17). The PMFs suggested the holo-heterotrimer had the highest binding affinity (ΔG) −159.4 kJ mol^−1^, followed by the holo-homotrimer (−104.5 kJ mol^−1^), whereas the apo forms were markedly weaker (−66.1 and −62.7 kJ mol^−1^, respectively) (Table 4).

**Table 4:** Apparent Binding Free Energy (ΔG_app) Derived from PMF Dissociation Profiles

Trimer	ΔG
Homotrimer	−104.52
Heterotrimer	−159.40
Apo-homotrimer	−66.13
Apo-heterotrimer	−62.70

Values represent relative association strength rather than absolute binding energies due to the one-dimensional reaction coordinate used. Values were derived from chain B, either an α1(I) or α2(I), uncoupling from chains A and C, which were both α1(I). Simulations were carried out in apo- and holo-type I collagen trimer isoforms.

However, the one-dimensional coordinate used in the umbrella sampling does not fully represent the conformational rearrangements accompanying trimer association or dissociation. Consequently, the PMFs reflect relative dissociation barriers rather than absolute binding energies. To provide a more comprehensive energetic assessment, additional enthalpic and free energy analyses were undertaken. Interchain interaction enthalpies calculated using the INTAA method showed that the calcium-bound heterotrimer possessed stronger total interchain interactions (−255.7 kJ mol^−1^) than the calcium-bound homotrimer (−234.6 kJ mol^−1^), a difference of −21.1 kJ mol^−1^ (Fig. 10 *C*). The greatest difference in stabilization occurred at the α2(I)-α1(I) (B-C) interface (−14.9 kJ mol^−1^). The additional stability was primarily attributed to hydrophobic contacts between MET-69 (from LEU-66 to MET) and MET-46, and favorable interactions near the C2-C3 disulfide bridge. THR-68 (from ASN-65 to THR) contributed additional solvent-exposed hydrogen bonding, improving the local electrostatic environment.

### Alchemical relative free energy perturbation calculations quantify heterotrimer stabilization

To quantify the thermodynamic contribution of α2(I)-specific residues to trimer stability, thermodynamic integration simulations were performed. Two interface substitutions characteristic of the α2(I) chain (ASN-65 to THR and LEU-66 to MET) were evaluated individually and in combination in both the apo and holo states. Each alchemical transformation was propagated for 200 ns under identical force field and solvation conditions to ensure direct comparability.

Relative binding free energies (ΔΔGbind) were derived from paired simulations of the trimeric complex (“complex route”) and corresponding monomeric chains (“solvent route”), with calcium dependence calculated as ΔΔΔGCa^2+^ = ΔΔGbind(holo) – ΔΔGbind(apo). In the holo systems, the combined mutations stabilized the heterotrimer relative to the homotrimer by ΔΔGbind = −5.6 kJ mol^−1^, whereas in the apo systems the difference was negligible (−0.9 kJ mol^−1^), resulting in a calcium-dependent stabilization of ΔΔΔGCa^2+^ = −4.7 kJ mol^−1^ (Table 5). At the simulation temperature of 300 K, this ΔΔGbind(holo) corresponds to approximately 2.2 RT units and an equilibrium constant (Krel) of approximately 9.5, indicating a 9.5-fold energetic preference for heterotrimer formation (51). This energetic preference is essentially eliminated under calcium depletion (ΔΔGbind = −0.9 kJ mol^−1^, Krel ≈1.4), demonstrating the role of calcium in biasing trimer composition. The calcium-dependent difference (ΔΔΔGCa^2+^ = −4.7 kJ mol^−1^, approximately 1.9 RT units) represents a 6.6-fold enhancement of heterotrimer preference upon calcium binding. This moderate but consistent energy difference (52) indicates that the α2(I)-specific residues confer a measurable thermodynamic advantage to trimer formation in the presence of calcium, which complements the structural constraints imposed by the cysteine code.

**Table 5:** Summary of FEP Calculations for Alchemical Thermodynamic Integration Energy Perturbation ΔΔG_bind_ = ΔG_trimer_ − ΔG_monome**r**_

Mutations	Ca^2+^ Status kJ/mol	ΔG_Trimer kJ/mol	ΔG_Monomer kJ/mol	ΔΔG_bind kJ/mol kJ/mol	ΔΔΔG_Ca^2+^ kJ/mol
ASN-65→THR	Apo	248.7	249.1	−0.4	–
ASN-65→THR	Holo	244.3	257.4	−13.1	–
LEU-66→MET	Apo	77.7	78.2	−0.5	–
LEU-66→MET	Holo	79.7	72.2	7.5	–
Sum of both mutations	Apo	326.4	327.3	**−0.9**	–
Sum of both mutations	Holo	324.0	329.6	**−5.6**	–
Holo − Apo	–	–	–	–	**−4.7**

Values in bold were used to calculate the equilibrium constant (Krel)

## Discussion

**Figure 11:**
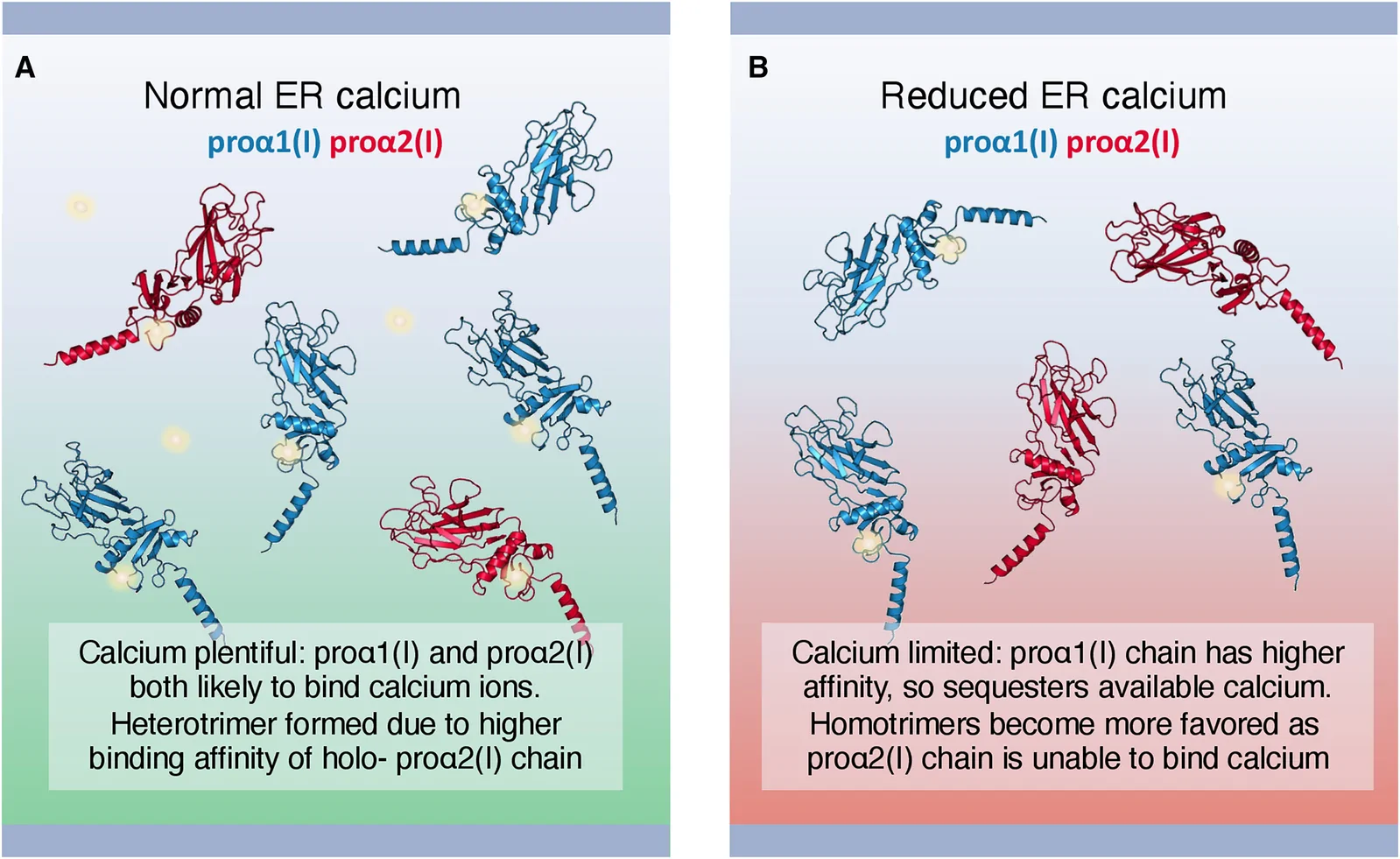
Proposed model for calcium regulation of type I collagen hetero- versus homotrimerization. (*A*) Heterotrimers are energetically favored in normal ER calcium concentrations, where there is sufficient calcium to bind all proα1(I) and proα2(I) monomers. (*B*) Under calcium-depleted conditions, energetic preference for heterotrimers is lost, and if proα1(I) chains preferentially sequester available calcium due to their longer residence times, homotrimer formation may be favored. This model is supported by free energy calculations, calcium residence times, and structural dynamics simulations. For clarity, only the C-propeptides of the procollagen proα1(I) and proα2(I) chains are shown; triple-helical regions and N-propeptides are omitted. Other ER chaperones required for procollagen folding are not shown. The proα1(I) C-propeptide monomers are shown in blue and the proα2(I) monomers in red. Calcium ions are depicted as light-yellow spheres.

In the present study we have uncovered a calcium-dependent mechanism that determines whether type I collagen forms heterotrimers or homotrimers. Our approach, integrating multiple simulation approaches and analyses, reveals a hierarchical series of calcium-mediated effects: calcium binding stabilizes the hydrogen bonding network at chain interfaces, maintaining the proximity required for disulfide bond formation between cysteine residues; α1(I) chains bind calcium approximately twice as strongly as α2(I) chains; and α2(I)-specific residues confer a 9.5-fold thermodynamic advantage for heterotrimerization, which is essentially eliminated under calcium depletion. We propose a model whereby reduced calcium concentration in the endoplasmic reticulum would favor homotrimerization over heterotrimerization by allowing α1(I) chains to preferentially sequester available calcium ions (Fig. 11).

Previous studies have shown that the homotrimer will form in the absence of α2(I) chains due to genetic inactivation or epigenetic silencing of *COL1A2* (14,53,54) or by overproduction of the α1(I) chain, either artificially or due to a common *COL1A1* polymorphism (55,56). However gene dosage and the relative abundances of *COL1A1* and *COL1A2* mRNAs do not directly predict heterotrimerization versus homotrimerization (57,58), likely because *COL1* mRNA translation is coordinated by numerous cytosolic factors (59), and the α2(I) chain sequence itself favors heterotrimerization.

Collagens are co-translationally translocated into the lumen of the rough ER during biosynthesis. The entire chain must be translated before the C-propeptide can fold and mediate trimerization. Normal free ER calcium concentrations range from 0.5–2.0 mM in many different cell types. Calcium pumps and channels maintain calcium homeostasis (60). Calcium availability in the ER is buffered by calcium-binding proteins, primarily calsequestrin in skeletal and cardiac muscle, and calrecticulin in other tissues. Calcium-dependent collagen chaperones such as BiP/GRP78, GRP94, PDI, and calnexin also act as calcium stores (61). Previous sedimentation equilibrium experiments with recombinant C-propeptides lacking cysteines showed that homotrimerization occurred in 0.5 mM calcium, but that monomers predominated in the absence of calcium (20). Future studies with mixtures of α1(I) and α2(I) chains over a range of calcium concentrations could elucidate the minimum calcium concentration for heterotrimerization.

Chronic decreases in ER calcium concentrations can affect the unfolded protein response, leading to misfolded proteins and apoptosis (61). The importance of intracellular calcium homeostasis is demonstrated by the ER stress and dysregulated type I collagen synthesis caused by loss of *TMEM38B*/TRIC-B, an ER membrane cation channel, which results in osteogenesis imperfecta (OI) (62). However, ER calcium concentrations are decreased in many pathological states including diabetes, ischemia, cardiovascular disease, viral infections, asthma, liver disease, and cancer (61). Calcium homeostasis also becomes dysregulated in aging due to oxidative damage, decreased expression of the ER calcium ion pump SERCA, and alterations in calcium-sensing proteins (63,64,65). Hence age- and disease-associated decreases in ER calcium could lead to homotrimer formation. Chronic diseases can be associated with increased focal collagen production, and the corresponding production of the homotrimeric form could accelerate fibrosis, due to the increased resistance of the homotrimer to MMP-mediated turnover (15).

Fibroblasts characteristically produce abundant type I collagen, particularly during development and tissue homeostasis. In fibroblasts, calcium signaling is mechano-sensitive and responds to the cell’s three-dimensional environment (66), which is especially relevant for proliferation and migration of fibroblasts during wound healing and leads to cyclic fluctuations in the amount of free calcium available (67). Hence the chain-specific trimerization of type I collagen may be influenced by the mechanical environment; we previously noted evidence of type I collagen homotrimer synthesis in precontracted but not fully contracted tendon-like constructs (58).

In two diseases of the skin, Darier disease and Hailey-Hailey disease, mutations in the genes for the ER calcium ion pumps SERCA2 and SPCA1, respectively, lead to dysregulation of calcium homeostasis and phenotypic features such as the formation of warts, lesions, blisters, and malodorous plaques (68). It is unknown if homotrimeric type I collagen is present in these conditions, though a *COL1A1-PDGFB* fusion gene is implicated in similar skin conditions (69).

Type I collagen homotrimers promote metastasis and invasion in cancer cells by providing MMP-resistant pathways for cell migration (8,54). ER calcium concentrations are often decreased in cancer cells, creating apoptosis resistance (70). It may therefore be that this depleted ER calcium contributes to homotrimerization of type I collagen during malignant transformation.

Our reported changes in hydrogen bond formation and salt bridge formation also demonstrate how known type I collagen C-propeptide mutations can cause disease. Approximately 6.5% of OI patients have mutations in the C-propeptide. The OI-causing mutation P-1182-R (at position 63 in the α2(I) C-propeptide) is proximal to the ASN-64 residue (71,72), which is important in stabilizing the binding interface between chains. Replacing the neighboring small, neutrally charged proline with a bulky, positively charged arginine likely interferes with hydrogen bond formation and chain incorporation. The same principle applies for other OI-causing mutations. THR-1431 (located at position 213 in the α1(I) C-propeptide) is close to the ARG-218 residue that participates in hydrogen bonding with GLU-177 in the neighboring chain. TYR-1263 (located at position 144 in the α2(I) C-propeptide) (71) has two neighboring residues that participate in inter- and intrachain hydrogen bonding respectively. It may also be that these mutations interfere with the solvent accessible area or directly repel their neighbors.

While the combination of equilibrium and nonequilibrium simulations, alchemical free energy calculations, and enhanced sampling techniques employed in this work provide strong computational evidence for calcium-mediated control of trimerization, several avenues for experimental validation and extension remain. First, sedimentation equilibrium or isothermal titration calorimetry experiments with mixtures of recombinant α1(I) and α2(I) C-propeptides across a range of calcium concentrations could test our predictions of the calcium threshold for heterotrimerization and the predicted 9.5-fold preference. Second, cellular studies monitoring trimer composition in response to controlled ER calcium depletion (using thapsigargin or ionomycin) (73,74,75) would validate the physiological relevance of our model. Third, the simulations presented here focus on the C-propeptide; inclusion of the full-length triple helical domain (21) (or partial inclusion of the sequences near the C-terminus) in future studies could reveal how the differential stability of α1(I) and α2(I) helices further modulates calcium-dependent assembly. The α2(I) helix is less stable than α1(I), which could amplify the effects of calcium depletion on heterotrimerization. The stability of this region could be explored using the computational approaches we’ve used herein, adding to our understanding the multiple mechanisms governing type I trimerization. Finally, the one-dimensional distance coordinate used to generate the PMF profiles cannot fully capture the conformational complexity of trimer assembly. PMFs have been previously used to study tetramers with some caveats (76). Here, we interpreted the PMF profiles as representing dissociation barriers rather than absolute binding free energies, and thus, they should only be treated as relative comparisons. In future work, coarse-grained approaches could be used to better sample the collective variable (77).

Nevertheless, the consistency across our multiple independent computational approaches—equilibrium MD, steered MD, τRAMD, umbrella sampling, and thermodynamic integration—all demonstrating calcium-dependent preferential stabilization of heterotrimers, provides strong convergent evidence for the calcium-dependent mechanism proposed here and strengthens confidence that conclusions reflect genuine physical phenomena. Most critically, alchemical thermodynamic integration provides rigorous, pathway-independent quantification of α2(I)-specific contributions to binding. Furthermore, the relative nature of our comparisons (heterotrimer versus homotrimer; α1(I) versus α2(I)) ensures systematic errors largely cancel when computing differences.

From a translational perspective, our findings suggest that therapeutic interventions targeting ER calcium homeostasis might prevent or reduce pathological homotrimer formation. Calcium channel modulators, SERCA pump activators, or agents that enhance ER calcium buffering capacity could shift the equilibrium back toward heterotrimerization in disease contexts. Conversely, understanding the conditions favoring homotrimerization could inform tissue engineering applications where the distinct mechanical properties of homotrimeric collagen might be deliberately exploited. Further investigation of the relationship between calcium concentration and trimer composition in pathological tissues would be valuable for developing such targeted therapeutic strategies.

## Data and code availability

The data can be obtained from Emily J. Johnson (emily.johnson@liverpool.ac.uk) or from the corresponding author on request. Code is provided in the following link: https://github.com/CBFLivUni/EJohnson_calcium_collagen_trimerisation.

## Acknowledgments

This work made use of the Barkla High Performance Computing facilities at the University of Liverpool. The authors would also like to thank Professor Dan Rigden for his advice and support during the revisions of this paper. Finally, the authors acknowledge use of the Computational Biology Facility provided by Liverpool Shared Research Facilities, University of Liverpool.

This work was funded by the 10.13039/501100000268Biotechnology and Biological Sciences Research Council (BBSRC), UK (BB/M011186/1, 1945098).

## Author contributions

Conceptualization: E.J.J. and E.G.C.-L.; data curation: E.J.J.; formal analysis: E.J.J., S.X., J.V.d.S, and A.E.; funding acquisition: E.G.C.-L.; investigation: E.J.J., S. X., J.V.d.S, and A.E.; project administration: E.G.C.-L.; supervision: A.K.B. and E.G.C.-L.; visualization: E.J.J., S.X., and A.E.; writing – original draft: E.J.J. and E.G.C.-L.; writing – review & editing: E.J.J., S.X., J.V.d.S, A.E., A.K.B., and E.G.C.-L.

## Declaration of interests

The authors declare no competing interests.

## Supporting Material

Document S1. Figures S1–S17 and Tables S1–S5

Document S2. Article plus Supporting Material
