# Supplementary material for: Molecular dynamics reveals how calcium drives hetero- versus homodimerization of type I collagen
Source: Biophys J. 2026 Jan 20;125(5):1286–304. doi: 10.1016/j.bpj.2026.01.033 (PMC13351861; doi:10.1016/j.bpj.2026.01.033)
Supplement: Document S1. Figures S1–S17 and Tables S1–S5 [file mmc1.pdf]

Biophysical Journal, Volume *125*

Supplemental information

Molecular dynamics reveals how calcium drives hetero- versus homo-dimerization of type I collagen

Emily J. Johnson, Shangze Xu, João V. de Souza, Anthony Evans, Agnieszka K. Bronowska, and Elizabeth G. Canty-Laird

Supporting Material for "Molecular dynamics reveals how calcium drives hetero- versus homo-trimerisation of type I collagen"

Emily J Johnson[1,2,3], Shangze Xu[4], João V de Souza[4,5], Anthony Evans[2,3], Agnieszka K Bronowska[4,6] and Elizabeth G Canty-Laird[1]

[1]Department of Musculoskeletal and Ageing Science, Institute of Life Course and Medical Sciences, University of Liverpool, William Henry Duncan Building, 6 West Derby Street, Liverpool, L7 8TX, United Kingdom

[2]Computational Biology Facility, LIV-SRF, MerseyBio, University of Liverpool, Crown Street, Liverpool, L69 7ZB, United Kingdom

[3] Institute of Systems, Molecular and Integrative Biology, University of Liverpool, Liverpool L69 7ZB, United Kingdom

[4] Chemistry-School of Natural and Environmental Sciences, Newcastle University, Newcastle Upon Tyne, NE1 7RU, United Kingdom

[5] Current location: RxCelerate Ltd, Babraham Research Campus, Cambridge CB22 3FH, United Kingdom

[6] Newcastle University Centre for Cancer, Newcastle University, Newcastle Upon Tyne, NE1 7RU, United Kingdom

Supplementary Tables

Table S1: Time averaged structural properties calculated for the homotrimer, heterotrimer, apo-homotrimer and apo-heterotrimer across three replicates.

Trimer type	Backbone RMSD (nm)	Backbone-Rg (nm)
Holo-homotrimer	0.564 (0.015)	2.867 (0.007)
Holo-heterotrimer	0.511 (0.037)	2.878 (0.035)
Apo-homotrimer	0.632 (0.120)	2.877 (0.044)
Apo-heterotrimer	0.540 (0.032)	2.895 (0.021)

Each value represents the mean across three replicate trajectories, with standard errors shown in parentheses. Standard errors were estimated using block averaging with a block size of 50 ns, determined from analysis of the standard error dependence on block size (see Figure S5).

Table S2. Notable hydrogen bonds in the homotrimer and heterotrimer.

Trimer	Bond	Position in full length chain	Inter-chain/ intra-chain	Weight
Homotrimer	ARG 42.A - ASP 129.B	ARG 1260.A - ASP 1347.B	Inter-chain	2.00794
Homotrimer	ARG 39.B - ASN 61.C	ARG 1257.B - ASN 1279.C	Inter-chain	1.48413
Homotrimer	ARG 42.B - ASP 129.C	ARG 1260.B - ASP 1347.C	Inter-chain	1.1746
Homotrimer	ASP 43.A - CYS 64.B	ASP 1261.A - CYS 1282.B	Inter-chain	1.09524
Homotrimer	ALA 128.C - ARG 42.B	ALA 1346.C - ARG 1260.B	Inter-chain	0.650794
Homotrimer	ARG 42.C - ASP 129.A	ARG 1260.C - ASP 1347.A	Inter-chain	0.579365
Homotrimer	ALA 128.A - ARG 42.C	ALA 1346.A - ARG 1260.C	Inter-chain	0.515873
Homotrimer	ARG 42.A - ASP 67.B	ARG 1260.A - ASP 1285.B	Inter-chain	0.460317
Homotrimer	ASP 43.C - CYS 64.A	ASP 1261.C - CYS 1282.A	Inter-chain	0.444444
Homotrimer	ARG 39.C - ASN 61.A	ARG 1257.C - ASN 1279.A	Inter-chain	0.380952

Homotrimer	ARG 39.A - ASN 61.B	ARG 1257.A - ASN 1279.B	Inter-chain	0.301587
Homotrimer	ARG 39.B - GLN 62.C	ARG 1257.B - GLN 1280.C	Inter-chain	0.301587
Homotrimer	CYS 64.C - MET 46.B	CYS 1282.C - MET 1264.B	Inter-chain	0.246032
Homotrimer	PHE 245.A - THR 80.A	PHE 1463.A - THR 1298.A	Intra-chain	2.01587
Homotrimer	ILE 58.A - ILE 69.A	ILE 1276.A - ILE 1287.A	Intra-chain	2
Homotrimer	TYR 56.C - VAL 71.C	TYR 1274.C - VAL 1289.C	Intra-chain	2
Homotrimer	ILE 58.B - ILE 69.B	ILE 1276.B - ILE 1287.B	Intra-chain	2
Homotrimer	ILE 58.C - ILE 69.C	ILE 1276.B - ILE 1287.B	Intra-chain	2
Homotrimer	TYR 56.A - VAL 71.A	TYR 1274.A - VAL 1289.A	Intra-chain	2
Homotrimer	TYR 56.B - VAL 71.B	TYR 1274.B - VAL 1289.B	Intra-chain	1.98413
Homotrimer	CYS 41.B - THR 80.B	CYS 1259.B - THR 1298.B	Intra-chain	1.7619
Homotrimer	CYS 41.A - THR 80.A	CYS 1259.A - THR 1298.A	Intra-chain	1.68254
Homotrimer	CYS 41.C - THR 80.C	CYS 1259.C - THR 1298.C	Intra-chain	1.61905
Homotrimer	ARG 39.B - ASP 43.B	ARG 1257.B - ASP 1261.B	Intra-chain	1.53968
Homotrimer	ASP 43.B - CYS 47.B	ASP 1261.B - CYS 1265.B	Intra-chain	1.38889
Homotrimer	ASN 61.B - GLN 133.B	ASN 1279.B - GLN 1351.B	Intra-chain	1.24603
Homotrimer	ASP 43.C - THR 40.C	ASP 1261.C - THR 1258.C	Intra-chain	1.23016
Homotrimer	ASP 43.B - THR 40.B	ASP 1261.B - THR 1258.B	Intra-chain	1.14286
Homotrimer	ASN 61.A - GLN 133.A	ASN 1279.A - GLN 1351.A	Intra-chain	1.05556
Homotrimer	ASN 61.C - GLN 133.C	ASN 1279.C - GLN 1351.C	Intra-chain	1.03968
Homotrimer	ASP 43.C - CYS 47.C	ASP 1261.C - CYS 1265.C	Intra-chain	0.968254
Homotrimer	ASP 43.A - THR 40.A	ASP 1261.A - THR 1258.A	Intra-chain	0.912698
Homotrimer	ASN 61.B - ASP 67.B	ASN 1279.B - ASP 1285.B	Intra-chain	0.888889
Homotrimer	ARG 42.B - LEU 246.B	ARG 1260.B -LEU 1464.B	Intra-chain	0.873016
Homotrimer	ARG 39.C - PRO 60.C	ARG 1257.C - PRO 1278.C	Intra-chain	0.865079
Homotrimer	ASN 61.A - ILE 132.A	ASN 1279.A - ILE 1350.A	Intra-chain	0.857143
Homotrimer	ASN 61.C - ASP 67.C	ASN 1279.C - ASP 1255.C	Intra-chain	0.84127
Homotrimer	ASN 61.A - ASP 67.A	ASN 1279.A - ASP 1255.A	Intra-chain	0.84127
Homotrimer	ARG 42.C - MET 46.C	ARG 1260.C - MET 1264.C	Intra-chain	0.785714
Homotrimer	ARG 42.B - THR 142.B	ARG 1260.B - THR 1360.B	Intra-chain	0.769841
Homotrimer	ASP 43.A - CYS 47.A	ASP 1261.A - CYS 1265.A	Intra-chain	0.746032
Homotrimer	ARG 42.A - MET 46.A	ARG 1260.A - MET 1264.A	Intra-chain	0.68254
Homotrimer	ARG 42.C - LEU 246.C	ARG 1260.C - LEU 1464.C	Intra-chain	0.674603
Homotrimer	ARG 39.C - ASP 43.C	ARG 1257.C - ASP 1261.C	Intra-chain	0.650794
Homotrimer	ARG 39.A - ASP 43.A	ARG 1257.A - ASP 1261.A	Intra-chain	0.619048
Homotrimer	ARG 42.B - MET 46.B	ARG 1260.B - MET 1264.B	Intra-chain	0.595238
Homotrimer	ARG 42.C - THR 142.C	ARG 1260.C - THR 1360.C	Intra-chain	0.5
Homotrimer	ARG 39.B - GLN 62.B	ARG 1257.B - GLN 1280.B	Intra-chain	0.468254
Homotrimer	ASN 61.C - ASP 59.C	ASN 1279.C - ASP 1277.C	Intra-chain	0.468254
Homotrimer	ARG 39.B - PRO 60.B	ARG 1257.B - PRO 1278.B	Intra-chain	0.34127
Homotrimer	ASN 61.B - ILE 132.B	ASN 1279.B - ILE 1350.B	Intra-chain	0.293651
Heterotrimer	ARG 42.A - GLU 130.B	ARG 1260.A - GLU 1249.B	Inter-chain	2.45238
Heterotrimer	ARG 39.A - ASN 64.B	ARG 1257.A - ASN 1183.B	Inter-chain	1.59524
Heterotrimer	ASP 43.A - CYS 67.B	ASP 1261.A - CYS 1186.B	Inter-chain	1.26984
Heterotrimer	ARG 42.C - ASP 129.A	ARG 1260.C - ASP 1347.A	Inter-chain	0.984127
Heterotrimer	ARG 39.C - ASN 61.A	ARG 1257.C - ASN 1279.A	Inter-chain	0.984127
Heterotrimer	ARG 42.B - ASN 61.C	ARG 1161.B - ASN 1279.C	Inter-chain	0.896825
Heterotrimer	ASP 46.B - CYS 64.C	ASP 1165.B - CYS 1282.C	Inter-chain	0.746032
Heterotrimer	ARG 45.B - ASP 129.C	ARG 1164.B - ASP 1347.C	Inter-chain	0.587302
Heterotrimer	ARG 45.B - ASP 67.C	ARG 1164.B - ASP 1285.C	Inter-chain	0.103175
Heterotrimer	ASP 43.C - CYS 64.A	ASP 1261.C - CYS 1282.A	Inter-chain	0.103175

Heterotrimer	ASP 43.A - THR 40.A	ASP 1261.A - THR 1258.A	Intra-chain	2.15873
Heterotrimer	ARG 42.A - LEU 246.A	ARG 1260.A -LEU 1464.A	Intra-chain	2.01587
Heterotrimer	TYR 56.C - VAL 71.C	TYR 1274.C - VAL 1289.C	Intra-chain	2
Heterotrimer	TYR 56.A - VAL 71.A	TYR 1274.A - VAL 1289.A	Intra-chain	2
Heterotrimer	ILE 58.A - ILE 69.A	ILE 1276.A - ILE 1287.A	Intra-chain	2
Heterotrimer	TYR 59.B - VAL 74.B	TYR 1178.B - VAL 1193.B	Intra-chain	1.99206
Heterotrimer	ILE 61.B - ILE 72.B	ILE 1180.B - ILE 1191.B	Intra-chain	1.99206
Heterotrimer	ILE 58.C - ILE 69.C	ILE 1276.C - ILE 1287.C	Intra-chain	1.99206
Heterotrimer	ASP 46.B - THR 43.B	ASP 1165.B - THR 1162.B	Intra-chain	1.96032
Heterotrimer	ARG 39.C - PRO 60.C	ARG 1257.C - PRO 1278.C	Intra-chain	1.94444
Heterotrimer	ARG 42.B - PRO 63.B	ARG 1161.B - PRO 1182.B	Intra-chain	1.88889
Heterotrimer	CYS 41.C - THR 80.C	CYS 1259.C - THR 1298.C	Intra-chain	1.69841
Heterotrimer	ASP 59.C - GLN 62.C	ASP 1277.C - GLN 1280.C	Intra-chain	1.57143
Heterotrimer	CYS 41.A - THR 80.A	CYS 1259.A - THR 1298.A	Intra-chain	1.53968
Heterotrimer	ASP 43.A - CYS 47.A	ASP 1261.A - CYS 1265.A	Intra-chain	1.5
Heterotrimer	ASP 59.A - GLN 62.A	ASP 1277.A - GLN 1280.A	Intra-chain	1.47619
Heterotrimer	CYS 44.B - THR 83.B	CYS 1163.B - THR 1202.B	Intra-chain	1.46032
Heterotrimer	ARG 45.B - PHE 246.B	ARG 1164.B - PHE 1365.B	Intra-chain	1.36508
Heterotrimer	ARG 39.A - ASP 43.A	ARG 1257.A - ASP 1261.A	Intra-chain	1.33333
Heterotrimer	ASP 43.C - CYS 47.C	ASP 1261.C - CYS 1265.C	Intra-chain	1.30159
Heterotrimer	ASP 43.C - THR 40.C	ASP 1261.C - THR 1258.C	Intra-chain	1.27778
Heterotrimer	ASN 61.A - GLN 133.A	ASN 1279.A - GLN 1351.A	Intra-chain	1.24603
Heterotrimer	ASN 61.C - GLN 133.C	ASN 1279.C - GLN 1351.C	Intra-chain	1.03968
Heterotrimer	ASN 64.B - GLN 134.B	ASN 1183.B - GLN 1253.B	Intra-chain	1.03175
Heterotrimer	ASP 70.B - GLN 134.B	ASP 1189.B - GLN 1253.B	Intra-chain	1
Heterotrimer	ASP 67.A - GLN 133.A	ASP 1285.A - GLN 1351.A	Intra-chain	0.992063
Heterotrimer	ARG 42.C - LEU 246.C	ARG 1260.C - LEU 1464.C	Intra-chain	0.984127
Heterotrimer	ASN 61.A - ASP 67.A	ASN 1279.A - ASP 1285.A	Intra-chain	0.960317
Heterotrimer	ASN 61.C - ILE 132.C	ASN 1279.C - ILE 1350.C	Intra-chain	0.928571
Heterotrimer	ARG 45.B - LEU 49.B	ARG 1164.B - LEU 1168.B	Intra-chain	0.920635
Heterotrimer	ASN 64.B - ASP 70.B	ASN 1183.B - ASP 1189.B	Intra-chain	0.84127
Heterotrimer	ARG 39.C - ASP 43.C	ARG 1257.C - ASP 1261.C	Intra-chain	0.801587
Heterotrimer	ASN 64.B - THR 133.B	ASN 1183.B - THR 1252.B	Intra-chain	0.690476
Heterotrimer	ARG 42.C - MET 46.C	ARG 1260.C - MET 1264.C	Intra-chain	0.674603
Heterotrimer	ARG 42.A - MET 46.A	ARG 1260.A - MET 1264.A	Intra-chain	0.65873
Heterotrimer	ASN 61.A - ILE 132.A	ASN 1279.A - ILE 1350.A	Intra-chain	0.515873
Heterotrimer	ARG 42.B - ASP 46.B	ARG 1161.B - ASP 1165.B	Intra-chain	0.253968
Heterotrimer	ASP 67.C - GLN 133.C	ASP 1285.C - GLN 1351.C	Intra-chain	0.222222
Heterotrimer	ASN 61.C - ASP 67.C	ASN 1279.C - ASP 1285.C	Intra-chain	0.142857

Each trimer has three chains: A, B and C. In the homotrimer all three chains are α1. In the heterotrimer chains A and C are α1(I) and chain B is α2(I)α1(1) is denoted chain A, α2 in the heterotrimer and α1(2) in the homotrimer are denoted chain B, whilst α1(3) is denoted chain C. The weight corresponds to how conserved the bond was throughout the simulation, with higher weights corresponding to the most conserved bonds and lower weights corresponding to more transient bonds.

Table S3. ΔRMSF values between the apo- and holo- forms of the homotrimer and heterotrimer.

Trimer	Residue	Chain	Difference	Mean Holo	Mean Apo
Heterotrimer	26	A	0.1279	0.159567	0.287467
Heterotrimer	125	A	0.126733	0.173633	0.300367
Heterotrimer	25	A	0.116633	0.177433	0.294067
Heterotrimer	27	A	0.107333	0.151067	0.2584
Heterotrimer	126	A	0.101033	0.1857	0.286733
Heterotrimer	124	A	0.099167	0.190267	0.289433
Heterotrimer	129	A	0.082233	0.150167	0.2324
Heterotrimer	65	A	0.075333	0.1395	0.214833
Heterotrimer	187	A	0.066433	0.2024	0.268833
Heterotrimer	28	A	0.066	0.220633	0.286633
Heterotrimer	66	A	0.064833	0.137567	0.2024
Heterotrimer	64	A	0.061467	0.123267	0.184733
Heterotrimer	128	A	0.0591	0.156133	0.215233
Heterotrimer	103	A	0.051433	0.2087	0.260133
Heterotrimer	67	A	0.0501	0.107133	0.157233
Heterotrimer	102	B	0.1425	0.416633	0.559133
Heterotrimer	26	B	0.110533	0.190567	0.3011
Heterotrimer	25	B	0.101267	0.190033	0.2913
Heterotrimer	99	B	0.0898	0.306333	0.396133
Heterotrimer	101	B	0.0743	0.3144	0.3887
Heterotrimer	106	B	0.0651	0.1534	0.2185
Heterotrimer	104	B	0.059367	0.326	0.385367
Heterotrimer	68	B	0.059233	0.117267	0.1765
Heterotrimer	100	B	0.054067	0.250067	0.304133
Heterotrimer	75	B	-0.05	0.176933	0.126933
Heterotrimer	176	B	-0.05	0.1353	0.0853
Heterotrimer	58	B	-0.05013	0.188233	0.1381
Heterotrimer	56	B	-0.05347	0.1745	0.121033
Heterotrimer	82	B	-0.05433	0.174867	0.120533
Heterotrimer	178	B	-0.0555	0.184533	0.129033
Heterotrimer	86	B	-0.0561	0.2923	0.2362
Heterotrimer	54	B	-0.0562	0.2041	0.1479
Heterotrimer	48	B	-0.05913	0.200667	0.141533
Heterotrimer	112	B	-0.0618	0.235533	0.173733
Heterotrimer	124	B	-0.06367	0.268867	0.2052
Heterotrimer	123	B	-0.0668	0.2286	0.1618
Heterotrimer	125	B	-0.06723	0.224633	0.1574
Heterotrimer	81	B	-0.06733	0.176967	0.109633
Heterotrimer	80	B	-0.07107	0.210833	0.139767
Heterotrimer	122	B	-0.07117	0.2454	0.174233
Heterotrimer	87	B	-0.07897	0.1867	0.107733
Heterotrimer	117	B	-0.07913	0.270867	0.191733
Heterotrimer	113	B	-0.07937	0.261633	0.182267

Heterotrimer	51	B	-0.08027	0.239367	0.1591
Heterotrimer	116	B	-0.09653	0.280267	0.183733
Heterotrimer	114	B	-0.10797	0.357733	0.249767
Heterotrimer	115	B	-0.12227	0.350533	0.228267
Heterotrimer	28	C	0.106433	0.2417	0.348133
Heterotrimer	67	C	0.0889	0.1387	0.2276
Heterotrimer	125	C	0.0863	0.168733	0.255033
Heterotrimer	123	C	0.081333	0.269333	0.350667
Heterotrimer	124	C	0.077867	0.205033	0.2829
Heterotrimer	127	C	0.075467	0.135067	0.210533
Heterotrimer	64	C	0.069533	0.126767	0.1963
Heterotrimer	198	C	0.069433	0.1126	0.182033
Heterotrimer	65	C	0.067167	0.1599	0.227067
Heterotrimer	161	C	0.063267	0.1866	0.249867
Heterotrimer	126	C	0.057367	0.181267	0.238633
Heterotrimer	27	C	0.056867	0.171467	0.228333
Heterotrimer	162	C	0.056533	0.210433	0.266967
Heterotrimer	29	C	0.0538	0.127133	0.180933
Heterotrimer	98	C	-0.0515	0.403967	0.352467
Heterotrimer	104	C	-0.07797	0.375133	0.297167
Heterotrimer	103	C	-0.1076	0.457867	0.350267
Heterotrimer	102	C	-0.1169	0.4181	0.3012
Heterotrimer	99	C	-0.13567	0.417967	0.2823
Heterotrimer	100	C	-0.1395	0.465133	0.325633
Homotrimer	26	A	0.190767	0.182933	0.3737
Homotrimer	25	A	0.1724	0.179767	0.352167
Homotrimer	28	A	0.1613	0.225767	0.387067
Homotrimer	27	A	0.1572	0.167467	0.324667
Homotrimer	103	A	0.104833	0.1876	0.292433
Homotrimer	66	A	0.101533	0.151767	0.2533
Homotrimer	161	A	0.093567	0.196367	0.289933
Homotrimer	29	A	0.093433	0.131967	0.2254
Homotrimer	101	A	0.092467	0.3903	0.482767
Homotrimer	64	A	0.089133	0.138333	0.227467
Homotrimer	162	A	0.0819	0.220933	0.302833
Homotrimer	185	A	0.076267	0.165867	0.242133
Homotrimer	63	A	0.071433	0.1186	0.190033
Homotrimer	177	A	0.071033	0.153567	0.2246
Homotrimer	67	A	0.0696	0.116133	0.185733
Homotrimer	65	A	0.066233	0.1531	0.219333
Homotrimer	102	A	0.064767	0.2411	0.305867
Homotrimer	204	A	0.062433	0.0934	0.155833
Homotrimer	100	A	0.061233	0.2943	0.355533
Homotrimer	129	A	0.060233	0.147933	0.208167
Homotrimer	124	A	0.056167	0.2093	0.265467
Homotrimer	186	A	0.055833	0.1726	0.228433

Homotrimer	104	A	0.053667	0.236733	0.2904
Homotrimer	30	A	0.053567	0.115767	0.169333
Homotrimer	125	A	0.052467	0.188133	0.2406
Homotrimer	34	A	0.0523	0.1584	0.2107
Homotrimer	99	A	0.05	0.2543	0.3043
Homotrimer	28	B	0.124167	0.2363	0.360467
Homotrimer	27	B	0.1211	0.164667	0.285767
Homotrimer	25	B	0.107133	0.187767	0.2949
Homotrimer	26	B	0.094833	0.1858	0.280633
Homotrimer	87	B	0.091933	0.1013	0.193233
Homotrimer	65	B	0.073567	0.158233	0.2318
Homotrimer	64	B	0.0646	0.135233	0.199833
Homotrimer	66	B	0.0634	0.151833	0.215233
Homotrimer	163	B	0.061333	0.1714	0.232733
Homotrimer	125	B	0.0577	0.162333	0.220033
Homotrimer	164	B	0.056633	0.1501	0.206733
Homotrimer	46	B	0.056167	0.137767	0.193933
Homotrimer	124	B	0.056133	0.1806	0.236733
Homotrimer	67	B	0.052233	0.111233	0.163467
Homotrimer	104	B	-0.05113	0.301933	0.2508
Homotrimer	103	B	-0.16427	0.408333	0.244067
Homotrimer	28	C	0.158333	0.234133	0.392467
Homotrimer	25	C	0.146133	0.191	0.337133
Homotrimer	125	C	0.1292	0.195367	0.324567
Homotrimer	27	C	0.125	0.189933	0.314933
Homotrimer	26	C	0.1143	0.203833	0.318133
Homotrimer	66	C	0.106533	0.168733	0.275267
Homotrimer	126	C	0.104967	0.210033	0.315
Homotrimer	129	C	0.0945	0.177733	0.272233
Homotrimer	124	C	0.093333	0.217133	0.310467
Homotrimer	128	C	0.0933	0.188233	0.281533
Homotrimer	127	C	0.0884	0.190567	0.278967
Homotrimer	67	C	0.087133	0.130933	0.218067
Homotrimer	64	C	0.0862	0.158333	0.244533
Homotrimer	195	C	0.085	0.119433	0.204433
Homotrimer	123	C	0.072433	0.318767	0.3912
Homotrimer	65	C	0.0717	0.1742	0.2459
Homotrimer	103	C	0.071	0.341467	0.412467
Homotrimer	196	C	0.0612	0.100067	0.161267
Homotrimer	87	C	0.059733	0.135233	0.194967
Homotrimer	98	C	0.0553	0.342233	0.397533
Homotrimer	114	C	0.053733	0.132867	0.1866
Homotrimer	46	C	0.050167	0.144033	0.1942
Homotrimer	104	C	-0.05253	0.365333	0.3128

Results are sorted by trimer, then chain, then ΔRMSF value. Positive values are residues that were destabilised in response to calcium depletion and negative values were residues that were stablised in response to calcium depletion.

Table S4. Inter-trimer type differences in mean distance predictions.

**Contrast**	**Difference in mean distance predictions (median, 95% CrI)**
Chain interface A:C2 – B:C3	
Apo-homotrimer - Homotrimer	0.2 (0.19–0.21)
Apo-heterotrimer - Heterotrimer	0.25 (0.24–0.26)
Heterotrimer - Homotrimer	0.0044 (-0.0023–0.011)
Apo-heterotrimer - Apo-homotrimer	0.053 (0.046–0.06)
Chain interface B:C2 – C:C3	
Apo-homotrimer - Homotrimer	0.28 (0.28–0.29)
Apo-heterotrimer - Heterotrimer	0.1 (0.094–0.11)
Heterotrimer - Homotrimer	0.038 (0.032–0.044)
Apo-heterotrimer - Apo-homotrimer	-0.15 (-0.15–-0.14)
Chain interface C:C2 – A:C3	
Apo-homotrimer - Homotrimer	0.3 (0.3–0.31)
Apo-heterotrimer - Heterotrimer	0.21 (0.2–0.21)
Heterotrimer - Homotrimer	0.04 (0.033–0.047)
Apo-heterotrimer - Apo-homotrimer	-0.059 (-0.066–-0.052)

For each contrast, mean distance predictions were estimated for the stated trimer types using all draws from the model posterior. The differences in per-draw predictions between trimer type were calculated and summarised as the median plus 95% credible (quantile) intervals.

Table S5. Comparisons between umbrella sampling of calcium ion uncoupling from α1(I) or α2(I) chain

**Comparison**	**α1**	**α2**	**Difference α1 − α2**
ΔG_min (binding well)	−4.99	−3.52	−1.5 kcal mol^{-1}

Supplementary Figures

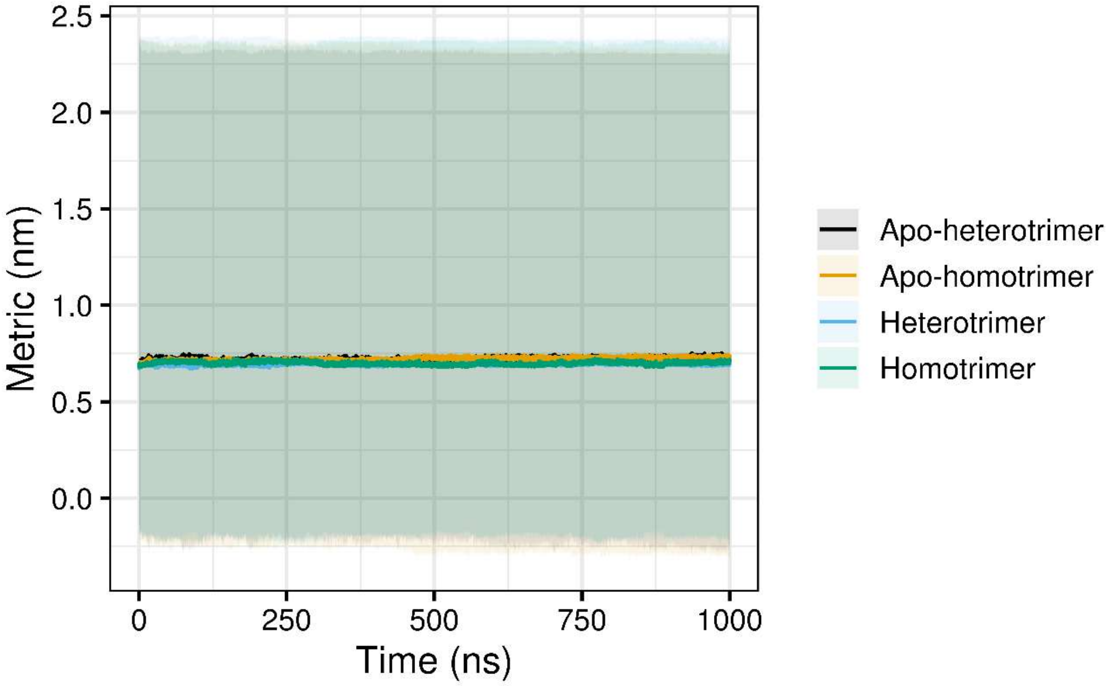

Figure S1: Prior predictive check for Bayesian linear autoregressive (AR) models. Implied model predictions from the weakly informative priors are shown, with no effect of time or trimer type imposed. Predictions for the metrics of interest (distance or RMSD) are mostly positive and fall within a plausible range of values. The means of the distributions are plotted as lines, with shaded regions indicating 95% credible intervals.

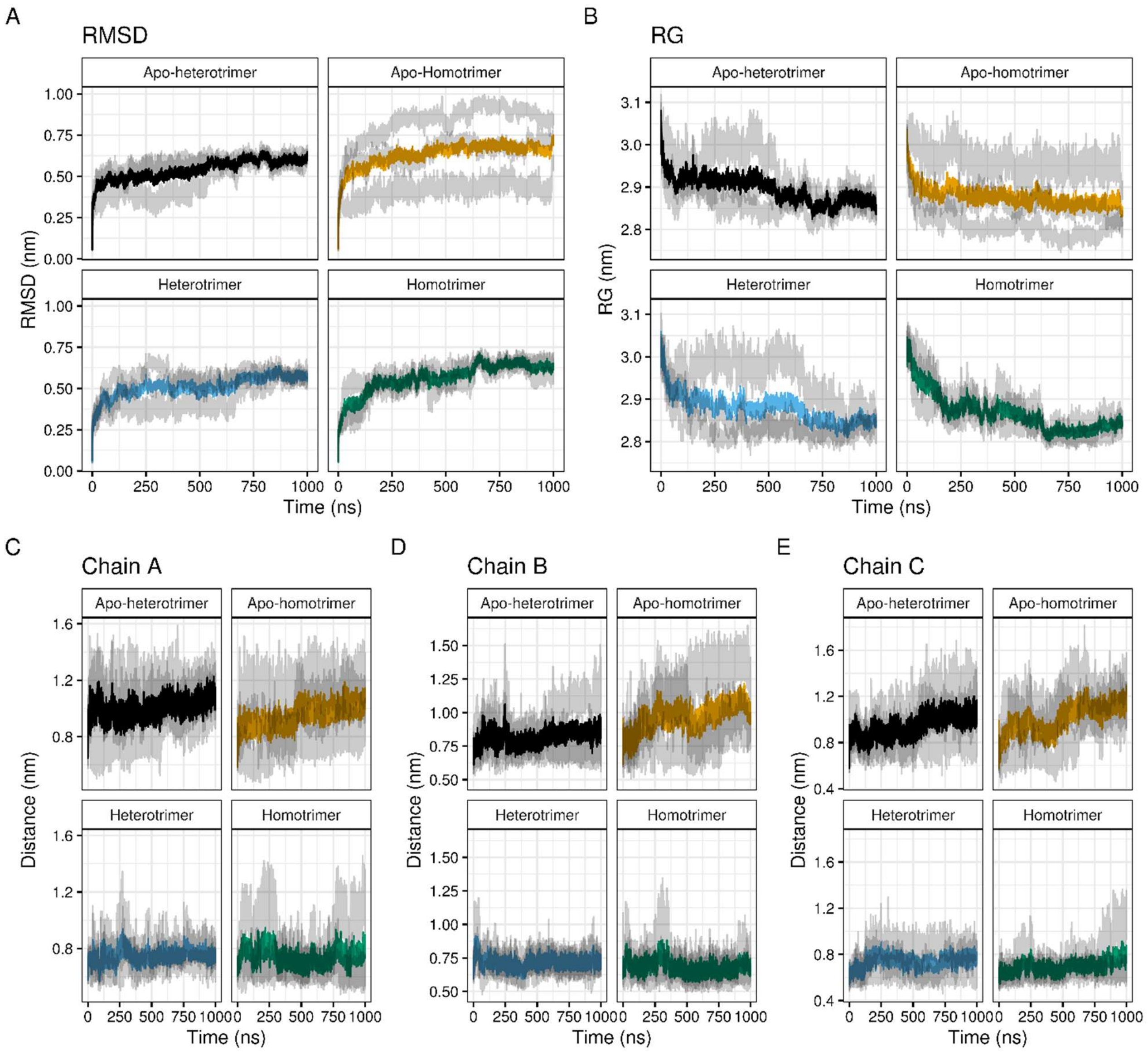

Figure S2: Posterior predictive checks on the fitted Bayesian linear autoregressive (AR) models. Posterior mean predicted distances are plotted as coloured lines with 95% credible intervals for RMSD (A), Rg (B), chain interface A (C), chain interface B (D) and chain interface C (E) distances for each trimer type. Raw data are plotted in grey for comparison, as separate replicate time series.

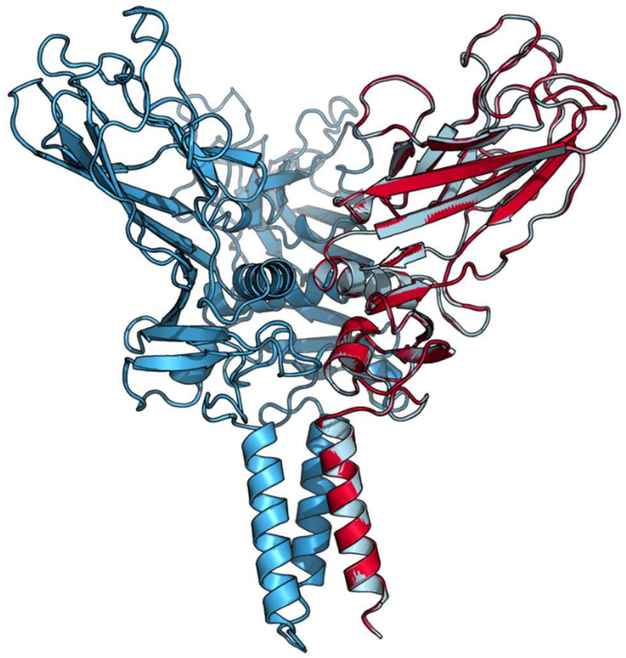

Figure S3: Comparison of the crystal structure of the homotrimeric type I collagen C-propeptide and the homology model of the heterotrimeric C-propeptide (SWISS-MODEL). The two structures are overlaid; in the heterotrimer the α2(I) chain is shown in red, in the homotrimer the corresponding chain is shown in light blue.

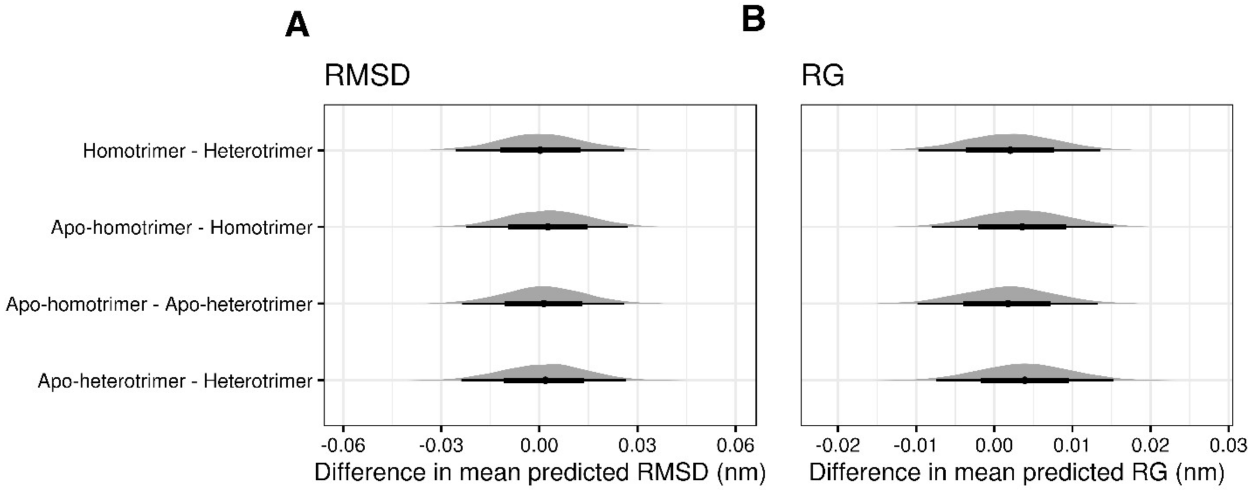

Figure S4: Posterior prediction contrasts between trimer types. Distributions of contrasted posterior predictions of RMSD (A) and Rg (B) independent of time, are plotted as kernel density estimation curves, alongside point intervals indicating the median, 66% and 95% credible intervals of the differences.

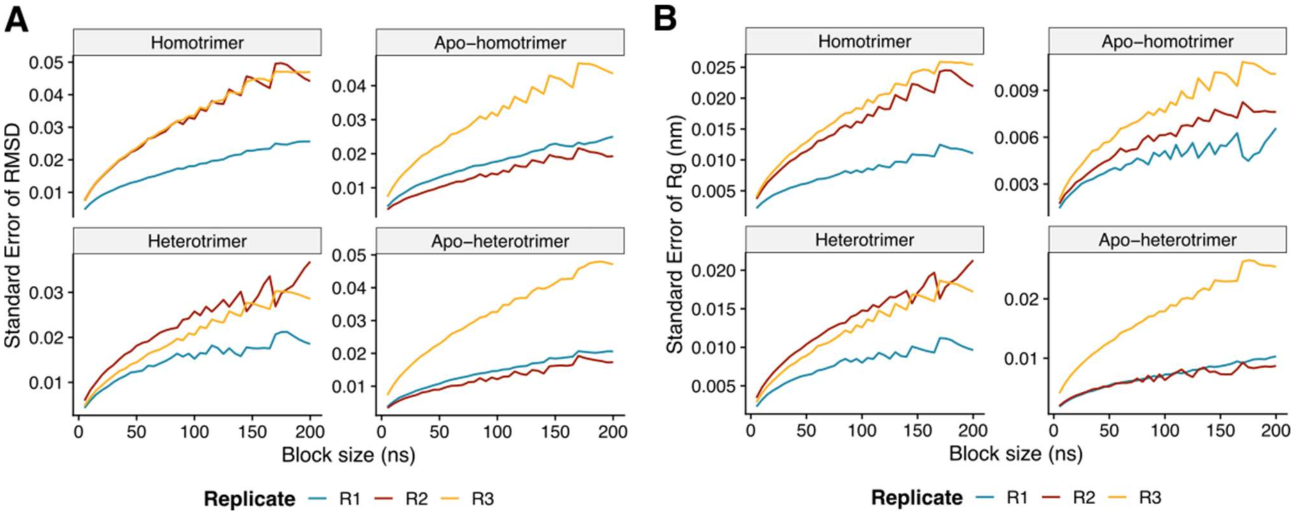

Figure S5: Block Error Analysis: Standard Error (SE) vs Block Size. The SE of the mean RMSD (A) and Rg (B) was evaluated as a function of block size for each replicate trajectory of the holo-homotrimer, apo-homotrimer, holo-heterotrimer, and apo-heterotrimer systems. The coloured lines represent individual replicates. For each trimer type, SE estimates stabilised at block sizes between 40–60 ns, after which variability in SE increased substantially. A block size of 50 ns was therefore selected for subsequent block averaging and summaries of equilibrium simulations.

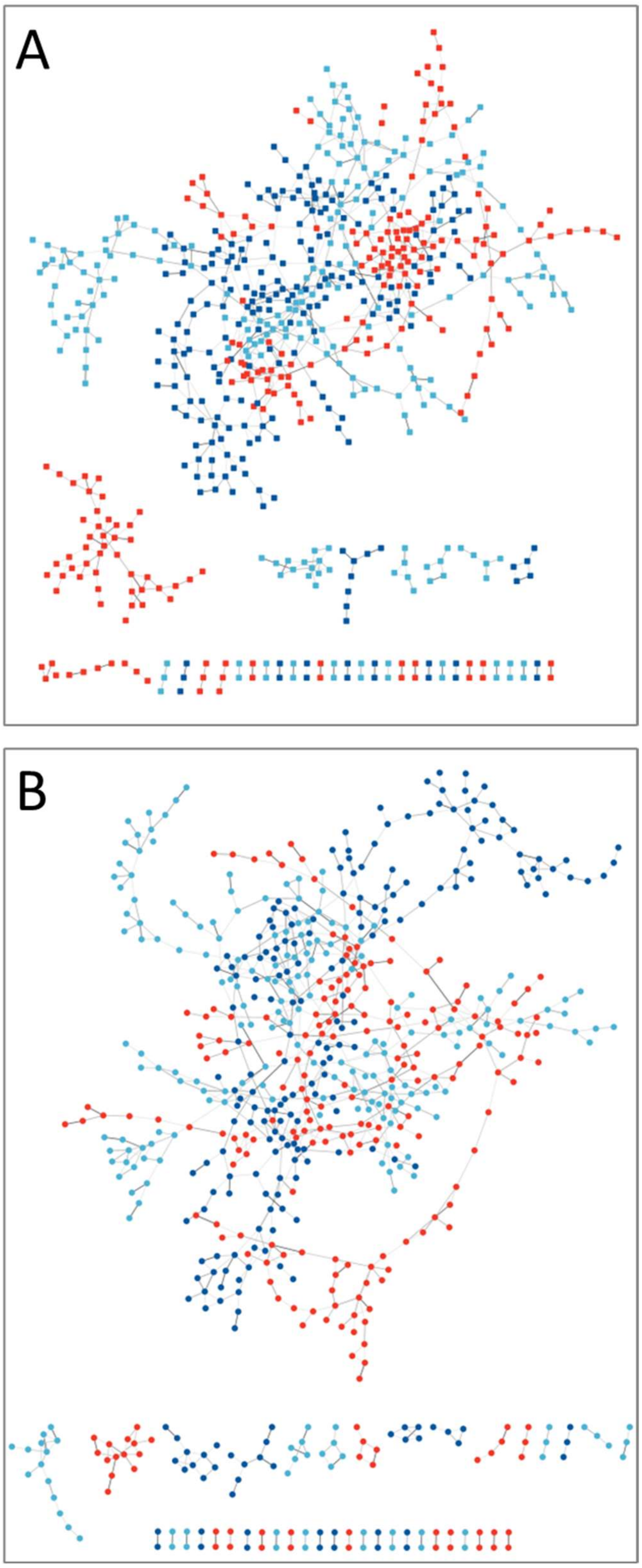

Figure S6: Cytoscape chimera full hydrogen bonding networks. A: Heterotrimer. B: Homotrimer. Red denotes the α2(I) chain for the heterotrimer (A) and the α1 B chain for the homotrimer (B). Blues represent the other α1 chains in each trimer (chains A and C).

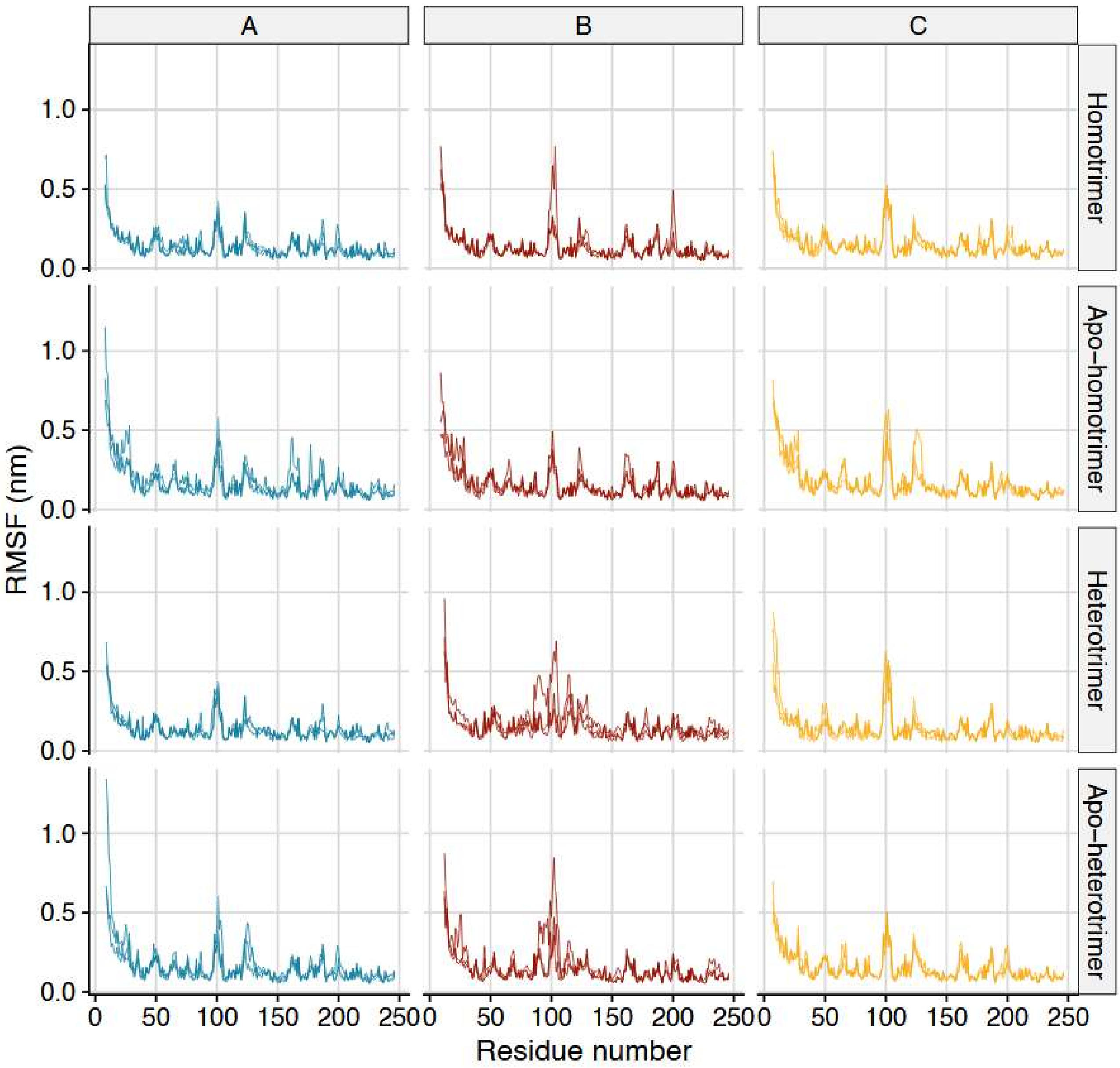

Figure S7: Backbone RMSF (nm) per trimer and chain. Each panel represents a different trimer and chain combination. There are three traces per panel, representing each replicate. The traces are coloured by chain. Each trimer has three chains: chains A, B and C. In the homotrimer all three chains are α1(I). In the heterotrimer chains A and C are α1(I) and chain B is α2(I). Chain A is shown in blue, chain B is shown in red and chain C is shown in yellow.

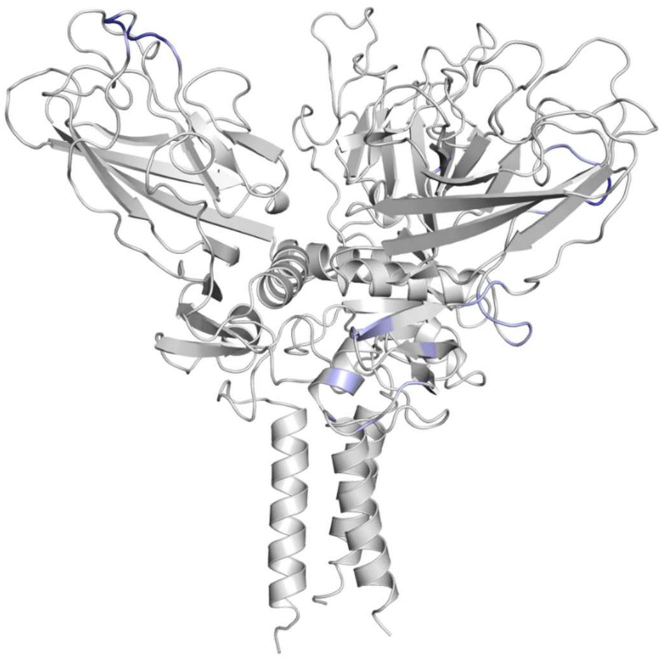

Figure S8: Residues that were stabilised by calcium depletion in the heterotrimer (shown in light blue: ΔRMSF < -0.05 or dark blue: ΔRMSF < -0.1). Most were confined to the exterior of the α2(I) chain.

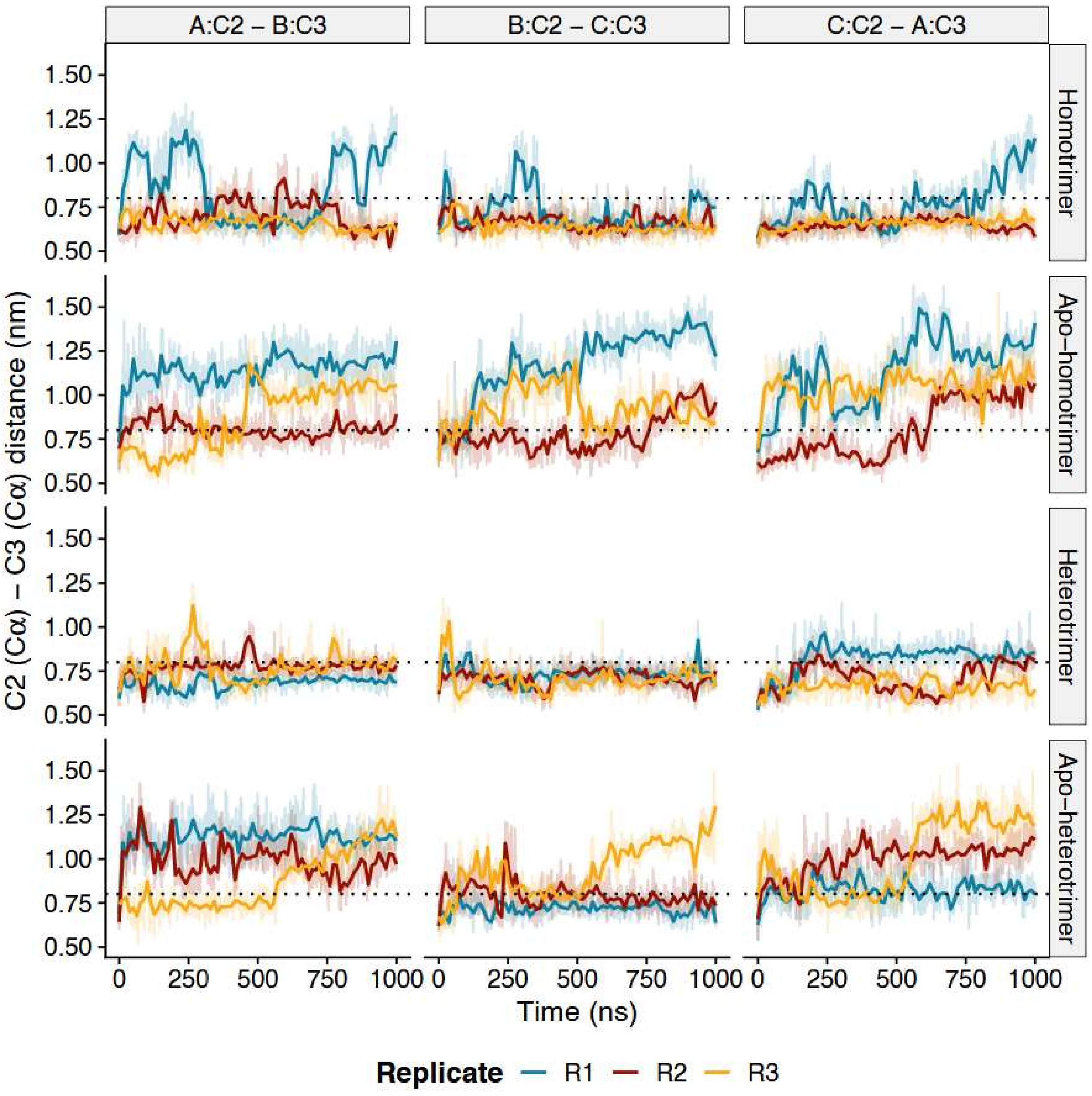

Figure S9: C2-C3 Cα distances per trimer and chain interface. Each panel shows a combination of trimer and chain interface, with distances between C2-C3 alpha carbons displayed as a time series, with each coloured trace representing a different simulation replica. The thick lines show the moving average of 20 ns, while the thinner lines show the distance every 1 ns. The dotted black line is at 0.8 nm, beyond which disulphide bonds are unlikely to form.

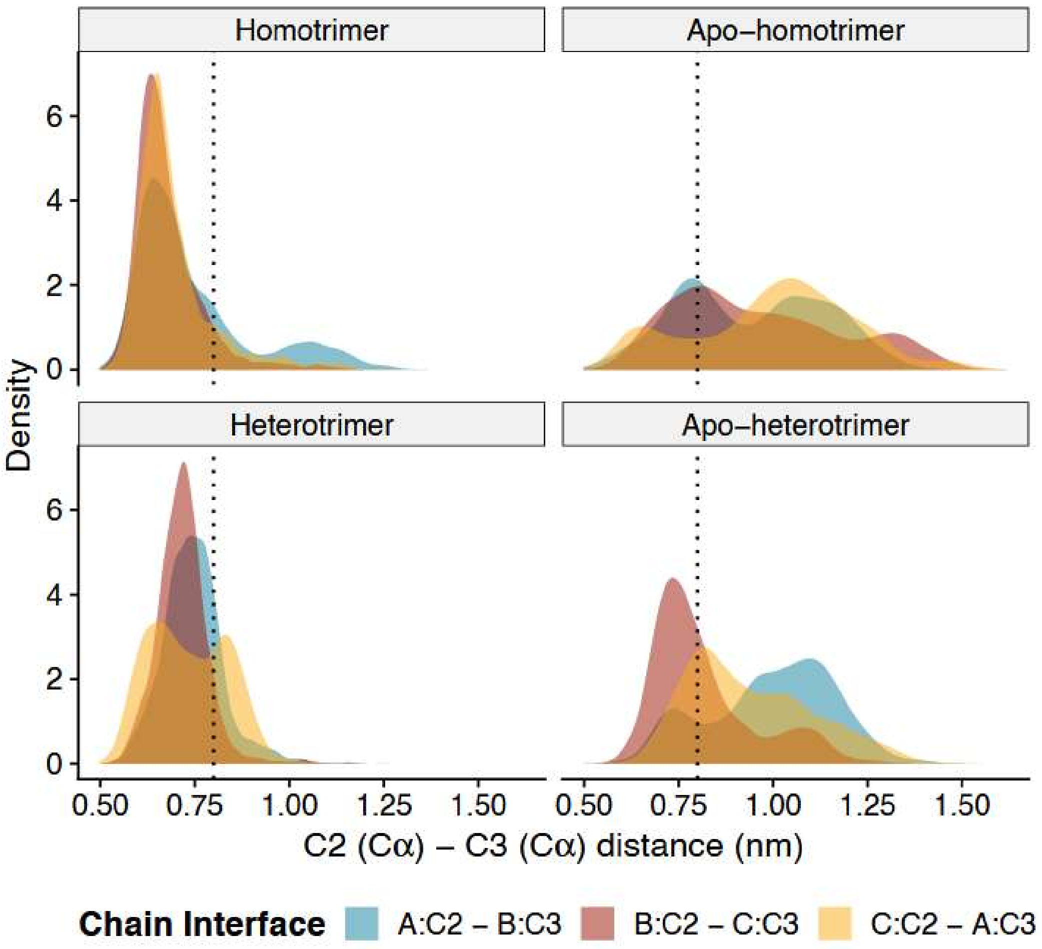

Figure S10: Distribution of C2-C3 Cα distances. The distribution of C2-C3 Cα distances is shown using a kernel density estimate (KDE) function with combined data of all three replicas. Each panel represents a different trimer. The dotted black line is at 0.8 nm, beyond which disulphide bonds are unlikely to form.

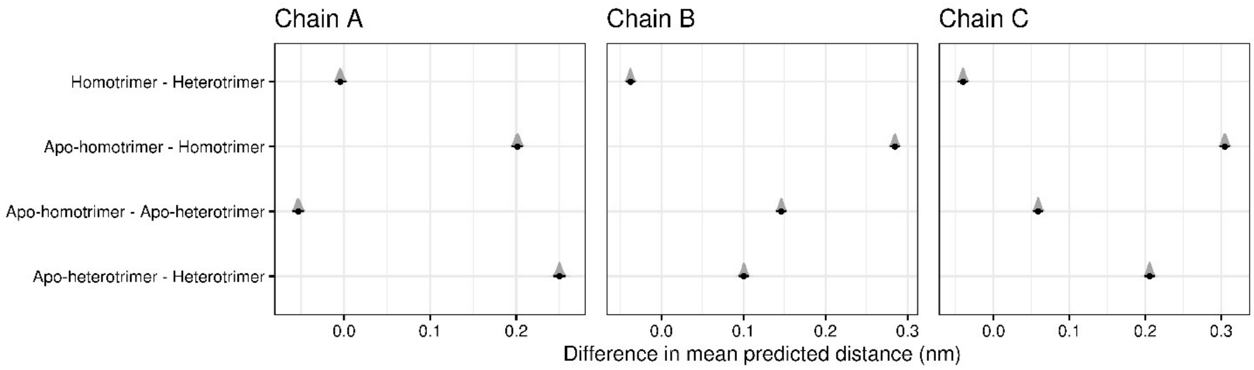

Figure S11: Posterior prediction contrasts between trimer types. Distributions of contrasted posterior predictions of chain interface distances, independent of time, are plotted as kernel density estimation curves, alongside point intervals indicating the median, 66% and 95% credible intervals of the differences.

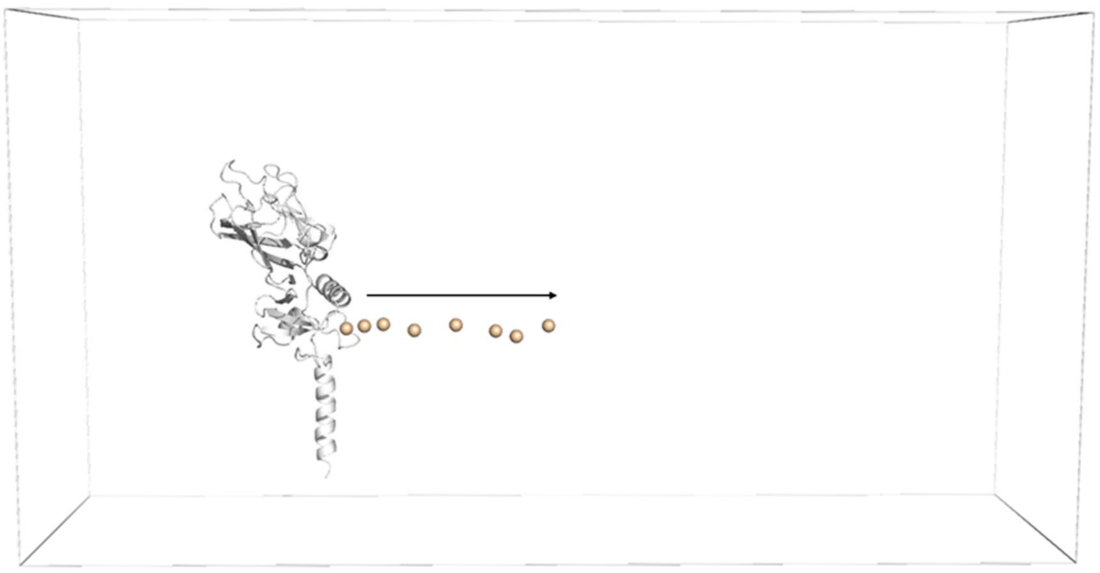

Figure S12: Centre of mass pulling simulation for a calcium ion extracted from its binding site in a monomer by steered molecular dynamics. An α1(I) monomer is shown in white and the calcium ion as a wheat-coloured sphere. The box defines the periodic boundary conditions. Multiple frames of the calcium moving away are shown along the arrow, which was the direction of the pull force.

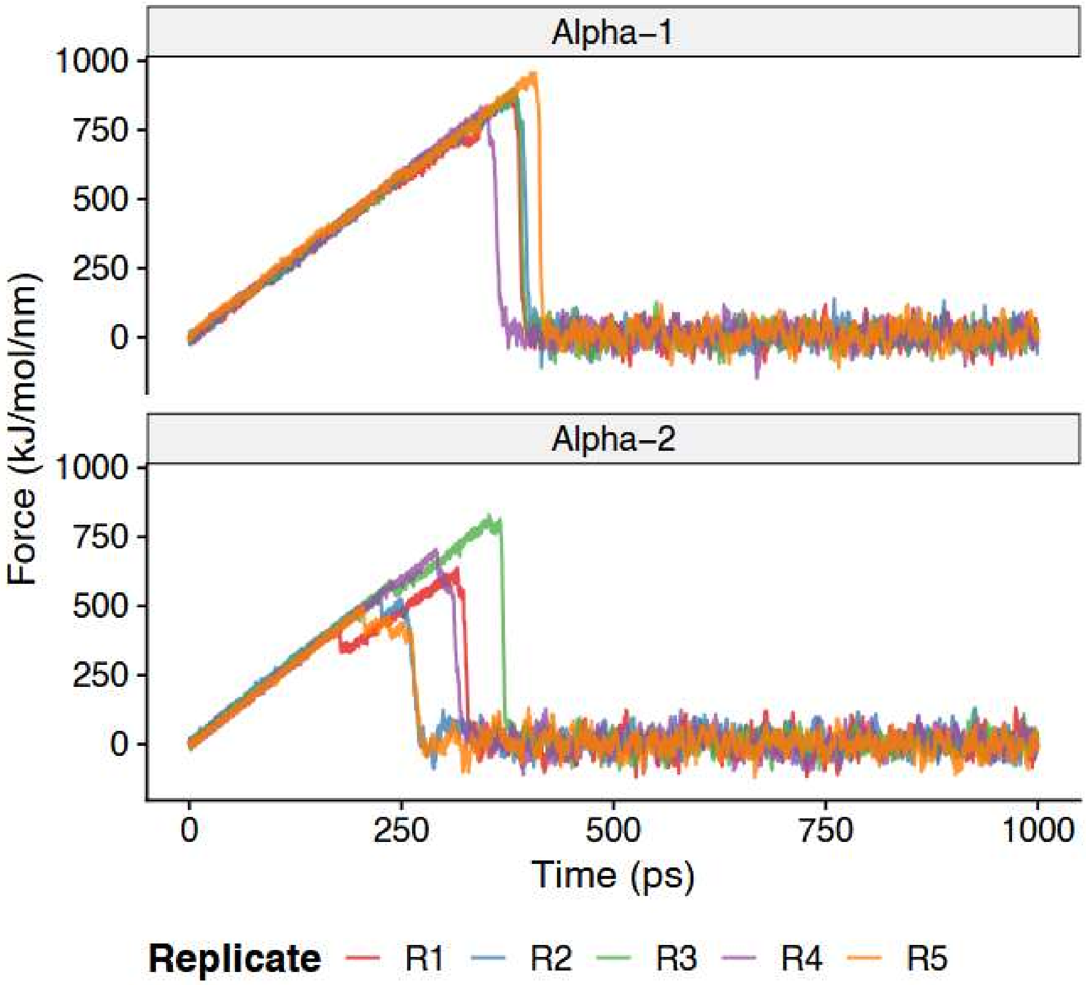

Figure S13: Pulling force per replicate for COM pulling simulations of calcium ion detaching from α1(I) or α2(I) chain. Each panel shows a different chain. The replicates (denoted 'R1', 'R2', etc.) are shown as individual traces.

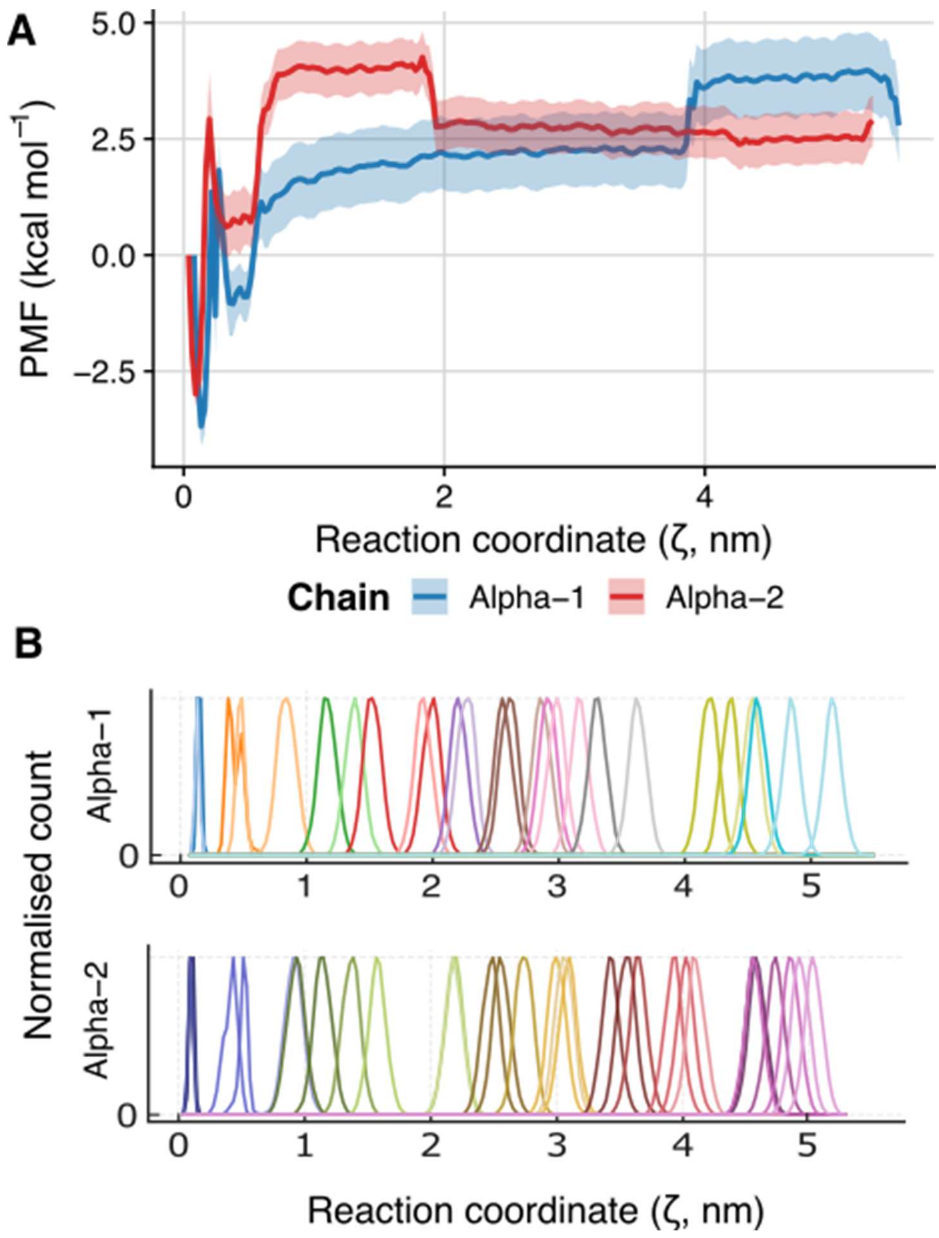

Figure S14: PMFs and umbrella sampling histograms for Ca^{2+} dissociation from the α1(I) and α2(I) chain, obtained via weighted histogram analysis (WHAM). A) The PMF is shown as a solid line for the α1(I) (blue) and α2(I) chains (red), the shaded regions denote the bootstrapped ± 1 SD uncertainties (100 resamples).. The bootstrapped SD bands are narrow (< 0.5 kcal mol^{-1}) in the bound region and widen in the unbound region. However, differences between the two were modest. The deeper α1(I) well could suggest lower bound-state energy. Consistently, t-RAMD yields ~2x longer residence times and steered molecular dynamics shows higher rupture forces for α1(I), implying additional pathway-dependent hidden barriers. These could arise from coordination and local gating, that are not fully captured by the 1D reaction coordinate. B) Normalised umbrella sampling histograms for the α1(I) and α2(I) windows, respectively, illustrating overlap along the reaction co-ordinate.

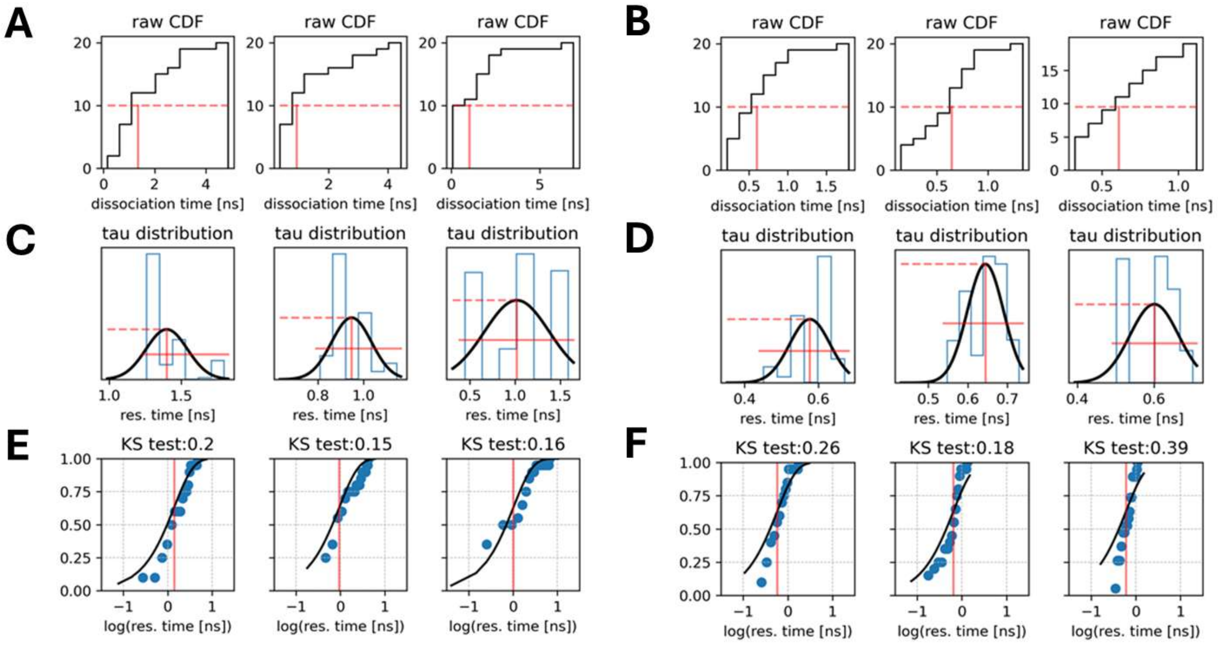

Figure S15: τRAMD statistical analysis for the α1(I) and α2(I) chains. A, B: Analysis of the time at which 50% of the trajectories had dissociated for the α1(I) (A) and α2(I) chain (B). C, D: Fit of a normal distribution to the data for the α1(I) (C) and α2(I) chain (D). E, F: Kolmogorov–Smirnov test results for the for the α1(I) (E) and α2(I) chain (F). The line is the Poisson cumulative distribution function and the blue dots the cumulative density function (CDF).

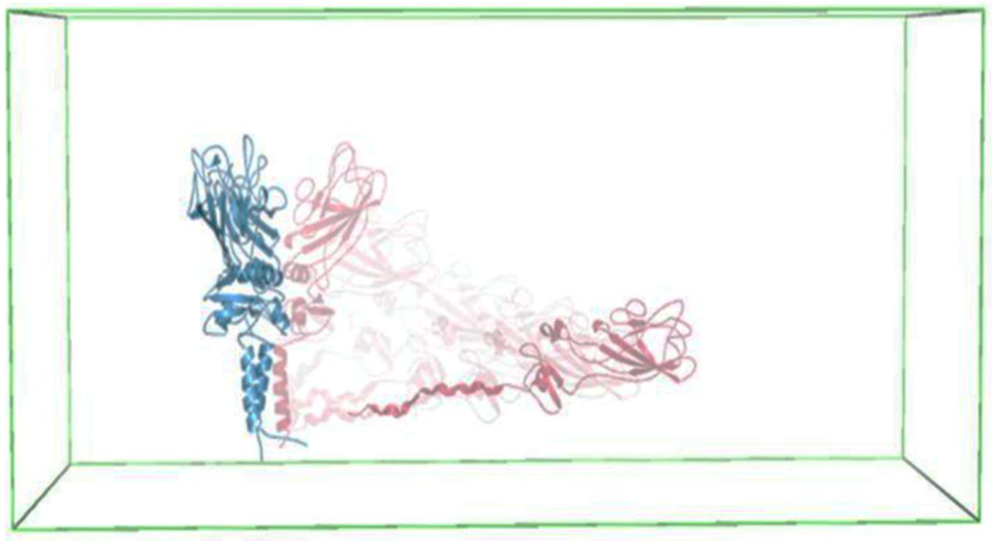

Figure S16: Sample unbinding pathway for the heterotrimer. An α2(I) chain is dissociating from two α1(I) chains along the collective variable (z-axis). The end configuration is shown as the darkest shade of red; the earliest configuration is shown as the next darkest shade. The α1(I) chains are shown in blue.

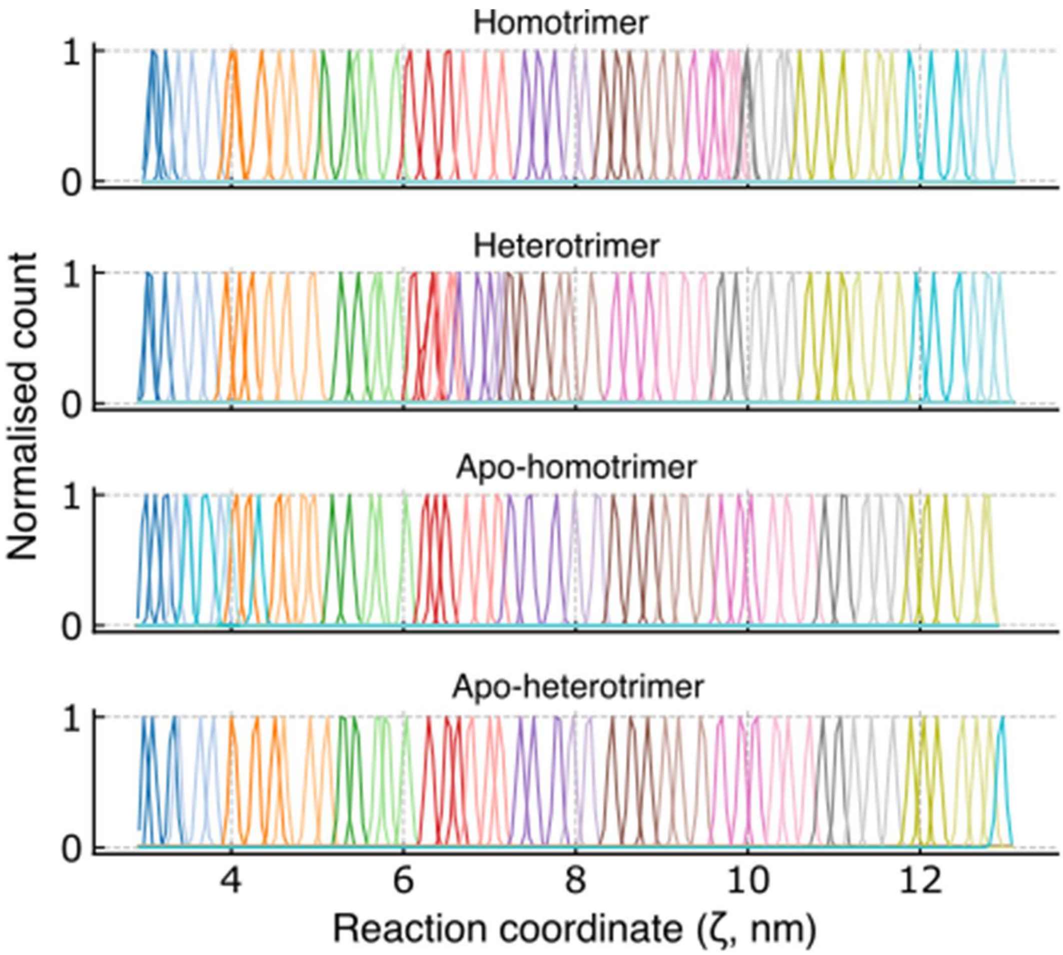

Figure S17: Histograms of the sampling windows for trimer pulling simulations. Histograms show the distribution of umbrella sampling windows used to construct the potential of mean force (PMF) profiles in Figure 10B. The reaction coordinate (ζ, nm) corresponds to the separation of the α2(I) chain (or the corresponding α1(I) chain in homotrimers) from the remaining two chains along a one-dimensional pulling pathway. Each coloured bin represents an individual umbrella sampling window, and the degree of overlap between adjacent windows indicates adequate sampling of configurational space for WHAM analysis. The trimers are a homotrimer, heterotrimer, apo-homotrimer and apo-heterotrimer respectively.
