# Supplementary material for: Molecular dynamics reveals how calcium drives hetero- versus homodimerization of type I collagen
Source: Biophys J. 2026 Jan 20;125(5):1286–304. doi: 10.1016/j.bpj.2026.01.033 (PMC13351861; doi:10.1016/j.bpj.2026.01.033)
Supplement: Document S2. Article plus Supporting Material [file mmc2.pdf]

Biophysical Journal
Article

Biophysical Society

Molecular dynamics reveals how calcium drives hetero- versus homodimerization of type I collagen

Emily J. Johnson,[1,2,3] Shangze Xu,[4] João V. de Souza,[4] Anthony Evans,[2,3] Agnieszka K. Bronowska,[4,5] and Elizabeth G. Canty-Laird[1,*]

[1]Department of Musculoskeletal and Ageing Science, Institute of Life Course and Medical Sciences, University of Liverpool, William Henry Duncan Building, 6 West Derby Street, Liverpool L7 8TX, UK; [2]Computational Biology Facility, LIV-SRF, MerseyBio, University of Liverpool, Crown Street, Liverpool L69 7ZB, UK; [3]Institute of Systems, Molecular and Integrative Biology, University of Liverpool, Liverpool L69 7ZB, UK; [4]Chemistry-School of Natural and Environmental Sciences, Newcastle University, Newcastle Upon Tyne NE1 7RU, UK; and [5]Newcastle University Centre for Cancer, Newcastle University, Newcastle Upon Tyne NE1 7RU, UK

ABSTRACT Type I collagen is the main structural protein of vertebrates and forms molecular trimers from the *COL1A1* and *COL1A2* gene products, proα1(I) and proα2(I), during biosynthesis. Calcium ions are required for trimers to form. The amino acid sequence of the C-propeptide of collagen, which is removed before collagen fibril formation, initially drives heterotrimerization. Abnormal homotrimeric type I collagen is associated with age-related diseases including cancer, fibrosis, and musculoskeletal and cardiovascular conditions, but the circumstances under which the homotrimer may form are poorly understood. Here, we used molecular dynamics simulations of the C-propeptide protein structure to show that inter- and intrachain hydrogen bonding is affected by loss of calcium and that this leads chains to become destabilized, particularly at the interfaces of each chain. Loss of calcium resulted in increased distances between the cysteine residues that form interchain disulfide bonds, preventing the formation of these bonds. Pulling simulations and modeling of calcium dissociation from monomers showed that calcium ions were more strongly bound to the α1(I) than the α2(I) chain. However, enhanced sampling methods implied the α2(I) chain has a higher trimer affinity than a third α1(I) chain in the presence of structural calcium. To quantify assembly thermodynamics, we computed relative binding free energies by alchemical thermodynamic integration, demonstrating that α2(I)-specific residues at the interchain interface conferred a measurable thermodynamic advantage to trimer formation in the presence of calcium. Hence, although heterotrimerization is normally favored, in reduced calcium conditions the homotrimer can form by sequestering available calcium to the α1(I) chains. This study provides a molecular explanation for a calcium-based mechanism driving heterotrimerization versus homotrimerization of type I collagen.

SIGNIFICANCE Type I collagen is the most abundant protein in the human body. Abnormal homotrimers contain three α1(I) chains, compared with normal heterotrimers with two α1(I) and, one α2(I). The homotrimers are implicated in cancer, fibrosis, and musculoskeletal and cardiovascular disease. Using molecular dynamics simulations and alchemical thermodynamic integration, we reveal how calcium concentration regulates trimer composition: heterotrimers are predicted to form preferentially when calcium is abundant (9.5-fold preference), but disruptions in endoplasmic reticulum calcium homeostasis—common in aging and disease—favor homotrimers because α1(I) chains bind calcium more strongly. This calcium-dependent mechanism could explain why homotrimers appear in pathological conditions and could provide molecular insights into collagen assembly disorders.

INTRODUCTION

Type I collagen is normally a heterotrimer composed of two α1(I) chains and one α2(I) chain, derived from the *COL1A1* and *COL1A2* genes respectively. N- and C-terminal globular propeptide domains flank a 300-nm-long right-handed triple-helical domain that supercoils around a central axis with a pitch ranging from 10/3 to 7/2 depending on proline content (1). The helical region has a repeating Gly-X-Y

Submitted May 19, 2025, and accepted for publication January 16, 2026.

*Correspondence: elizabeth.laird@liverpool.ac.uk

"João V. de Souza's present address is RxCelerate Ltd, Babraham Research Campus, Cambridge CB22 3FH, UK."

Editor: Frauke Graeter

https://doi.org/10.1016/j.bpj.2026.01.033

© 2026 The Author(s). Published by Elsevier Inc. on behalf of Biophysical Society.
This is an open access article under the CC BY license (http://creativecommons.org/licenses/by/4.0/).

amino acid structure, where X and Y are often proline and hydroxyproline, respectively. The lack of a side chain on glycine allows the bulky side chains of the other amino acids to occupy the outer positions, enabling tight packing (2,3). The C- and N-terminal propeptides confer solubility to the chains, preventing premature aggregation. The C-propeptide guides the trimerization process, as chain selection and alignment begins with C-propeptide trimerization, after which folding of the triple helix occurs from the C to N end (4). The propeptides are removed to facilitate assembly of trimeric type I collagen molecules into fibrils.

An abnormal homotrimeric form composed of three α1(I) chains has been reported in adult skin and embryonic tissues (5,6). However, the homotrimeric form is also associated with diseases such as cancer, osteoarthritis, osteoporosis, fibrosis, and Ehlers-Danlos syndrome (7,8). Molecular dynamics (MD) simulations of a 57-amino-acid region of the >1000-amino-acid triple-helical region have shown the homotrimer to be softer and more flexible (9). The homotrimeric helix freely rotates and forms kinks in the Gly-X-Y domain, which is predicted to lead to greater lateral distances between homotrimeric molecules in fibrils, consistent with experimental findings (10). Altered packing may be responsible for reported differences in intermolecular collagen cross-linking in the osteogenesis imperfecta murine (oim) mouse model (11–13), although the homotrimeric collagen is itself not responsible for bone fragility (14). Type I collagen homotrimer is resistant to proteolysis compared with the heterotrimer, and degradation by matrix metalloproteinase-1 (MMP-1) is approximately 10 times slower (15). The homotrimer however appears more sensitive to degradation under mechanical strain, while the heterotrimer is less sensitive (16,17). As the two trimeric forms of type I collagen demonstrate such different biophysical, dynamic, and structural properties, their physiological roles presumably differ, and synthesis needs to be tightly controlled to ensure the correct type of type I collagen is being produced.

The C-propeptide drives fibrillar collagen trimerization and determines trimer chain composition. In type III collagen, the chain recognition sequence coordinates trimerization and ensures that only α1(III) homotrimers form (18,19). This chain recognition mechanism does not however occur in type I collagen, where interchain interactions occur at key residues that form salt bridges (7), and trimer composition is partially governed by a network of disulfide bond-forming cysteines in the C-propeptide (20). The α1(I) chain C-propeptide contains eight cysteine residues (Cys 1–8), two of which participate in interchain disulfide bonding: C2 and C3. The α2(I) chain C-propeptide lacks the C2 residue and can only form one interchain disulfide bond. This ensures that only heterotrimers and α1(I) homotrimers can form and has been termed the "cysteine code." Interchain disulfide bonding is however neither necessary nor sufficient for triple-helix formation, as the triple-helical domain of the α2(I) chain is unable to trimerize when coupled to a homotrimerizing α1(I) C-propeptide, but the α2(I) C-propeptide can trimerize and permit folding of a coupled α1(I) chain triple-helical domain (21). There is however a key role for calcium ions in mediating trimerization, as in the absence of available calcium in solution no heterotrimers or homotrimers can form (20). The α1(I) and α2(I) C-propeptides contain a conserved calcium-binding loop coordinating a structural calcium ion that sits at subunit interfaces in the C-propeptide trimer. Indeed, a *COL1A1* mutation substituting a calcium-binding residue in the C-propeptide of the α1(I) chain prevents trimerization and results in perinatal lethality (20,22).

In the present study, equilibrium MD simulations along with enhanced sampling techniques and free energy calculations were used to study type I collagen C-propeptide stability, with and without the structural calcium bound, to investigate how the structural calcium guides trimerization and how calcium homeostasis might play a role in homotrimer production.

MATERIALS AND METHODS

Generating homology models

Homology models of the C-propeptide of heterotrimeric type I collagen were generated using the modified structure of the homotrimeric C-propeptide (PDB: 5K31) as a template (7). Mutations were reverted to the canonical sequence in PyMOL ("The PyMOL Molecular Graphics System," Version 2, Schrödinger, New York, USA) and incomplete side chains rebuilt in SWISS-PDB Viewer. Glycerol and an excess chloride ion were also stripped. SWISS-MODEL (23) was used to create the homology model for the C-propeptide of heterotrimeric type I collagen. To create apo (Ca^{2+} depleted) versions of the proteins, the remaining calcium ions were also stripped. All trimers were simulated with the interchain disulfide bonds reduced. Monomers were created by extracting an α1(I) chain or the corresponding α2(I) chain.

Equilibrium MD simulation protocol

GROMACS 2019.3 (24,25) was used to run all simulations of type I collagen trimers and monomers in solution. The AMBER99SB-ILDN force field (26) was used to describe the system topology. Three replicates were carried out. Hydrogens were replaced with virtual sites to allow for a longer time step of 5 fs and sampling of longer timescales (27). A fourth replicate with explicit hydrogens was also carried out for examination of hydrogen bonding patterns.

Standard simulation setups and protocols associated with the AMBER99SB-ILDN force field were used to carry out the MD calculations: electrostatic interactions were calculated using the particle-mesh Ewald method, and short-range nonbonded interactions were cut off at 1.0 nm (28,29). Verlet neighbor search was used with an update interval of 20 fs (30). The LINCS algorithm was used to constrain all bonds during the simulations (31).

TIP3P water model was used to solvate the system. The systems were neutralized using potassium ions, with additional potassium and chloride ions added to simulate physiological salt concentrations in the ER. Energy minimization was carried out using the steepest descent algorithm and terminated after 50,000 steps or when the maximum force was reduced to 1000 kJ mol^{-1} nm^{-2}. Equilibration was carried out in two steps; the first step was a constant volume (NVT) ensemble where the protein and aqueous phase (water plus ions) were coupled to separate temperature baths at 310 K using the modified Berendsen thermostat (V-rescale). The second step was a constant pressure (NPT) equilibration with the Parrinello-Rahman barostat

to maintain the pressure isotropically at 1.0 bar. Production runs were carried out in the NPT ensemble for 1000 ns (resulting in 16,000 ns of trajectory data in total).

Proteins were made whole and jumps and periodic boundary conditions were removed; then, translational and rotational movements were also removed. Analyses (root mean-square deviation (RMSD), root mean-square fluctuation (RMSF), radius of gyration, interchain distances) used standard GROMACS tools; trajectories were visualized in VMD (32) and PyMOL.

Time series regression of MD data and further analysis

To compare differences in MD metrics between trimer types, accounting for the serial nature of the data, a Bayesian first-order linear autoregression (AR(1)) model was fitted using the *brms* v2.23.0 R package (33), utilizing the *Stan* probabilistic programming language (34). AR(1) terms were modeled for each replicate time series separately, with trimer types modeled as fixed effects. Weakly informative priors were utilized: β coefficients of trimer type effects were fitted with normal distribution priors with mean = 0 and standard deviation (SD) = 0.2, the AR(1) coefficient priors were specified as normal(mean = 0, SD = 0.3), the model intercept priors were specified as half-normal(mean = 0, SD = 1) with lower bound 0, and the model residual standard deviation priors were specified as exponential(rate = 1). Prior predictive checks revealed these priors imposed no effect of time and trimer type a priori, allowing any effects to be estimated primarily from the data while ensuring model predictions predominantly fell among a realistic range (Fig. S1).

Eight Monte Carlo Markov chains (MCMC) were used to fit the model posterior distribution, each with 1000 warmup and 1000 sampling iterations to yield 8000 draws per parameter. Model convergence was confirmed by ensuring R-hat statistics for all parameters were close to 1 as well as examining MCMC trace plots. Posterior predictive checks were performed using the *marginaleffects* v0.30.0 R package (35), to confirm that model predictions of expected metric values (means) were consistent with the data (Fig. S2). Counterfactual contrasts of expected value predictions were performed using the *tidybayes* v3.0.7 R package (36) for each trimer type comparison.

In addition to the above analysis, time-averaged structural properties were determined using a block-error approach, and block sizes were optimized using standard error convergence. Summary figures were produced using *ggplot2* (37) using the R programming statistical environment (version 4.3.2, R Core Team, 2023).

Enhanced sampling protocols

Enhanced sampling methods were applied to type I collagen C-propeptide monomers and trimers to characterize chain-chain and ion-protein interactions. τRAMD (38), steered molecular dynamics (SMD), and umbrella sampling (39) were employed sequentially to explore dissociation pathways and quantify interaction free energies.

For trimeric systems, to investigate the affinity of an α1(I) or α2(I) chain for its neighboring two chains, τRAMD was first used to sample unbinding directions by pulling a mobile chain away from two stationary chains along randomized vectors. Twenty-five τRAMD trajectories were performed for each replicate following the standard τRAMD protocol.

Representative τRAMD trajectories were used to define the collective variable for subsequent SMD and umbrella sampling. Trimers were solvated, neutralized, and equilibrated as in the equilibrium simulations and rotated such that the mobile chain was orientated along the unbinding pathway. Backbone restraints were applied to the two stationary chains. A spring constant of 1500 kJ mol^{-1} nm^{-2} and a pull rate of 0.05 nm ps^{-1} were used, with 5 center-of-mass (COM) pulling simulations carried out per system. Average force-time and rupture-force profiles were obtained from these trajectories.

Snapshots spaced at 0.1–0.2 nm along the COM separation coordinate (to a maximum distance of ~12 nm) were selected as starting structures for umbrella sampling. Due to computational resource limitations, umbrella sampling was carried out for one representative replicate per trimer system. Approximately 50 windows were simulated for 10 ns each, and the weighted histogram analysis method (WHAM) was used to construct the potential of mean force (PMF) (40). Statistical uncertainties in the PMFs were estimated using bootstrap resampling of the histograms. Bootstrapping was performed with 100 resamples to provide 95% confidence intervals for the free energy profiles (41).

To investigate calcium binding affinity for single α1(I) and α2(I) chains, the uncoupling of the structural calcium ion from the binding loop was analyzed using the same techniques. End-state coordinates from 10-ns production MD runs (three replicates) served as starting points ($n = 3$). Twenty-five τRAMD trajectories were performed per replicate using a 500-kJ mol^{-1} nm^{-2} pulling force applied to the ion in random directions until a 5-nm displacement from the loop was achieved. The relative residence time (τ_comp) was defined as the simulation time required for 50% of trajectories to exhibit complete dissociation. Results were analyzed using a freely available script (https://github.com/DKokh/tauRAMD), examining results for normality by visualizing the distributions and employing a Kolmogorov-Smirnov test.

For SMD of monomers, the calcium-binding loop backbone was position-restrained while side chains remained flexible. The calcium ion was pulled along the z-axis using a spring constant of 500 kJ mol^{-1} nm^{-2} and a pull rate of 0.005 nm ps^{-1}. Snapshots were extracted every ~0.2 nm up to a final COM distance of 5 nm, resulting in ~30 umbrella sampling windows. Umbrella sampling was again performed for one representative replicate per monomer type, with each window simulated for 10 ns. WHAM was used to obtain the corresponding free energy profiles, with the same bootstrapping protocol as above.

Alchemical relative binding free energy calculations

We computed relative binding free energies for heterotrimer formation using alchemical thermodynamic integration (TI) (42) via the thermodynamic cycle approach. Two interface mutations characteristic of the α2 chain (ASN-65 to THR and LEU-66 to MET) were evaluated individually in both complex (intact trimer) and solvent (monomer) routes for both apo- and holo-conditions.

Mutations were implemented using the pmx package (43) with the amber99sb-ildn-mut* force field and TIP3P water (44). Hybrid topologies were generated with mutate.py and generate_hybrid_topology.py and then processed with GROMACS (2023.2). Systems were energy minimized (50,000 steps, convergence at F_max <1000 kJ mol^{-1} nm^{-1}) and then equilibrated for 500 ps each in NVT (300 K, V-rescale thermostat) and NPT (1 bar, Parrinello-Rahman) ensembles. For holo simulations, to ensure comparable reference frames across mutations were investigated, harmonic distance restraints (1000 kJ mol^{-1} nm^{-2}) maintained Ca^{2+} coordination to three conserved heavy atoms within 4 Å.

All alchemical runs were performed as a single continuous λ with GROMACS (free_energy = yes). The MD time step was 2 fs, and each trajectory was propagated for 1 × 108 steps, giving a total simulation time of 200 ns. The coupling parameter started from the homo state at $\lambda_0 = 0$ and was increased linearly by $\Delta\lambda = 1 \times 10^{-8}$ per MD step (init_lambda = 0, delta_lambda = 0.00000001), reaching λ = 1 at the end of the run. Soft-core nonbonded potentials were used with α = 0.5, σ = 0.3 nm, and power = 1. Resulting dH/dayλ time series were numerically integrated over λ to obtain ΔG. Relative binding free energies were calculated as ΔΔG_bind = ΔΔG_complex − ΔΔG_monomer, with calcium dependence estimated as ΔΔΔG_Ca = ΔΔG_bind(holo) − ΔΔG_bind(apo) (Fig. 1).

Interchain interaction enthalpies

Procollagen heterotrimer (two α1(I) chains and one α2(I) chain) was modeled using the crystal structure of fibrillar procollagen type I

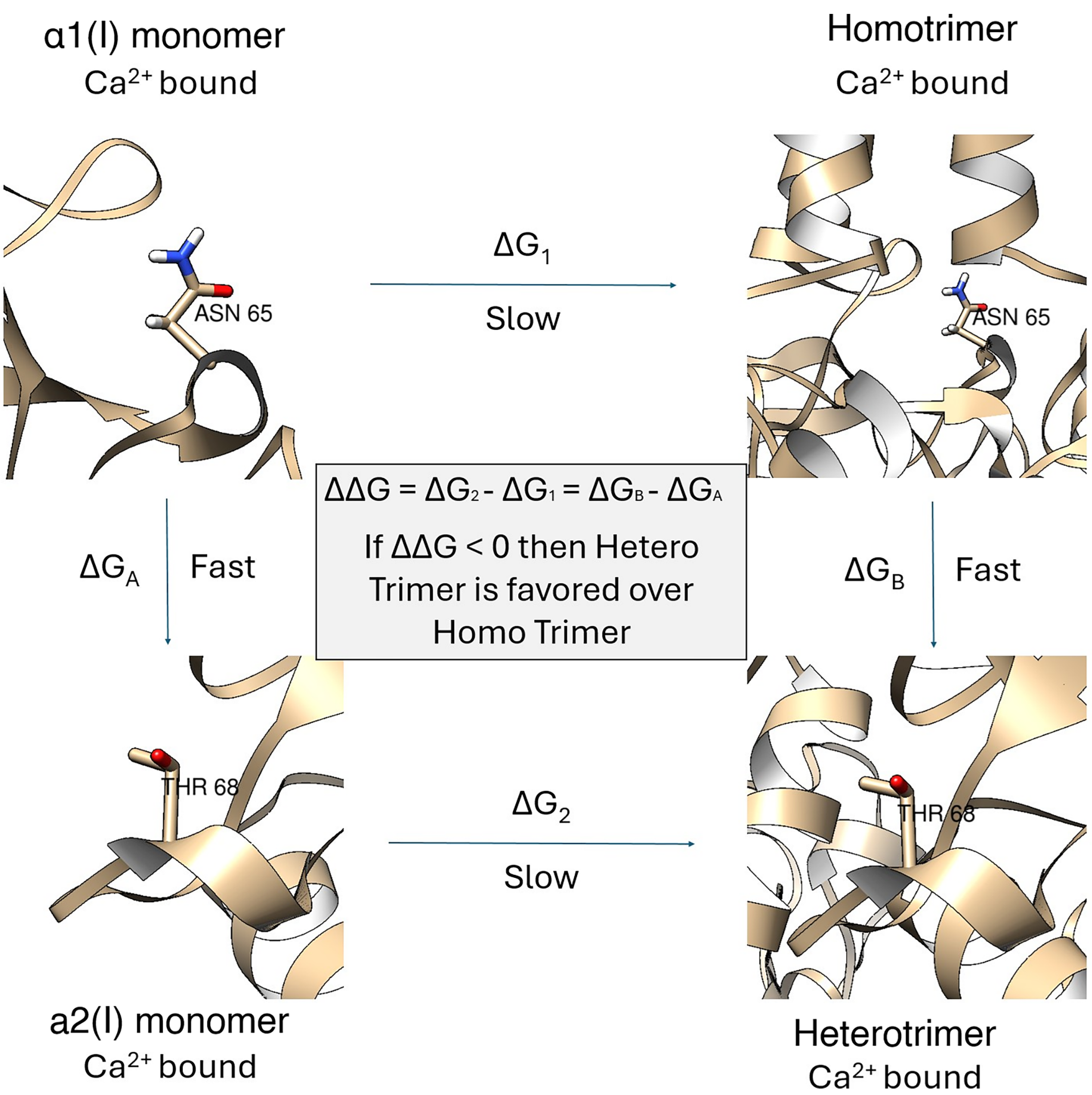

FIGURE 1 Thermodynamic cycle for relative binding free energy calculations. Top row: homotrimer and α1(I) chain; bottom row: heterotrimer and α2(I) chain. The left column is monomer, and the right column is trimer. Horizontal arrows ΔG_1 and ΔG_2 represent the assembly free energy of monomer to trimer (using slow TI); vertical arrows ΔGA and ΔGB represent alchemical mutations in the monomeric or trimer context (homotrimer to heterotrimer). Closure of the circuit gives the relative binding free energy (RBFE).

C-propeptide homotrimer (PDB: 5K31) and AlphaFold3 (45) model of α2(I) chains. The missing calcium cation was fitted, and both trimers were energy minimized. Interchain interaction enthalpy calculations for hetero- and homotrimers were performed using parameters derived from AMBER parm99 classical molecular mechanical force fields and a GB/SA implicit solvation model. All calculations were performed using INTAA webserver (46).

RESULTS

The C-propeptide shows increased RMSD but decreased radius of gyration over 1000 ns of simulation

A model for the type I collagen heterotrimeric C-propeptide was generated by homology modeling using the crystal structure of the homotrimeric form as a template (Fig. S3). MD simulations were performed for 1 μs for both the homotrimer (Fig. 2 *A*) and heterotrimer (Fig. 2 *B*), in either the holo or apo states. In all simulations, the backbone RMSD increased over time (Fig. 2 *C*), whereas the radius of gyration (Rg) showed a gradual decrease (Fig. 2 *D*), indicating compaction of the trimeric assemblies during equilibration. Individual replicates for RMSD (Fig. 2 *E*) and Rg are shown (Fig. 2 *F*).

Overall, the RMSD did not consistently differ between trimer types (Table 1; Fig. S4A). Holo forms were generally more stable than corresponding apo forms, although variability was high, particularly for the apo-homotrimer. Conversely, the homotrimer maintained a slightly tighter fold and appeared to be more compact than the heterotrimer,

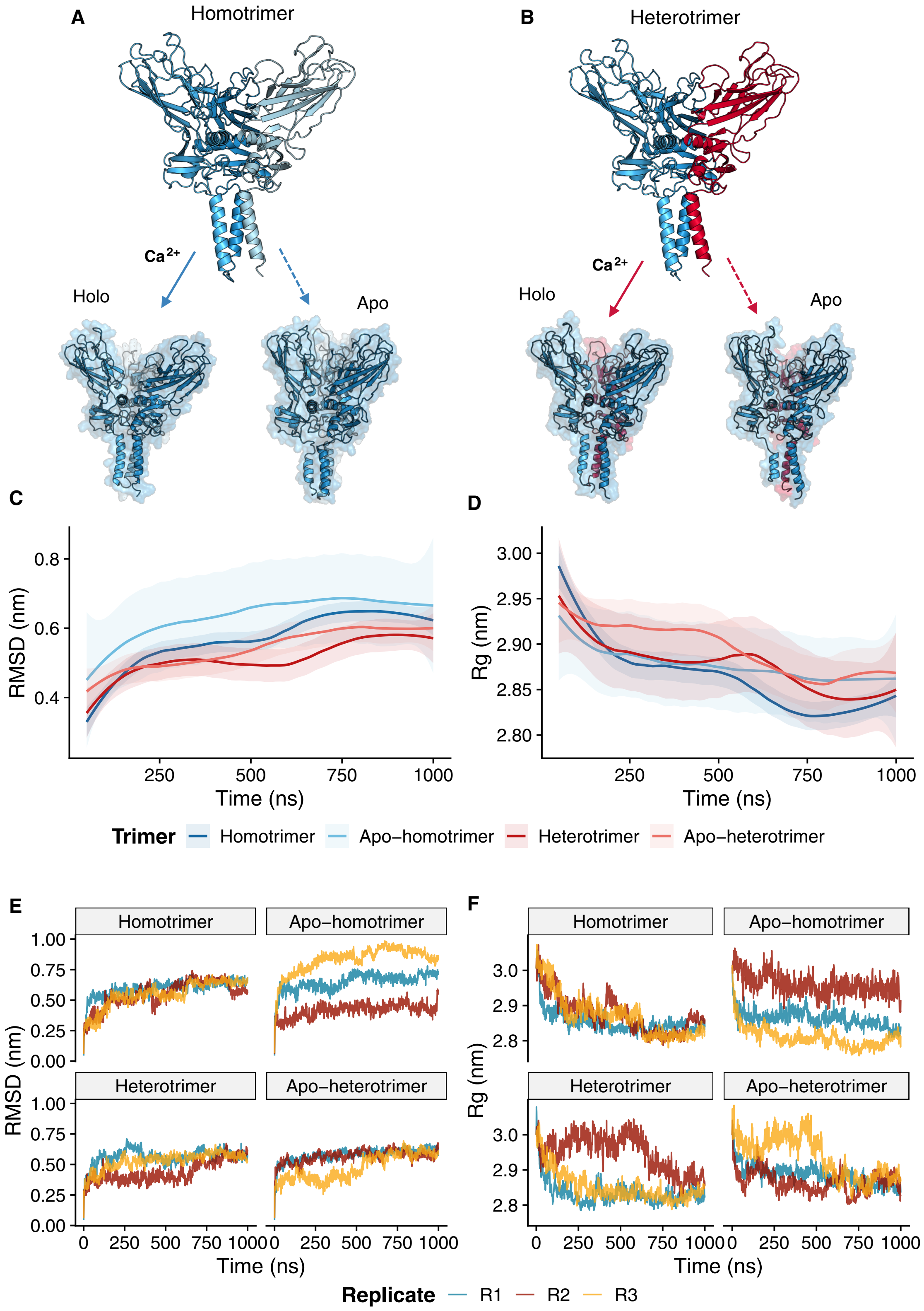

FIGURE 2 Molecular dynamics simulations of apo and holo version of the type I collagen C-propeptide homotrimers and heterotrimers. (*A* and *B*) Starting structures of the C-propeptide homotrimer (*A*) and heterotrimer (*B*) compared with the holo- and apo-trimers after 1000 ns of simulation. The α2(I) chain is

(legend continued on next page)

TABLE 1 Intertrimer Type Differences in Mean RMSD Model Predictions

Contrast	Difference in Mean RMSD Predictions (Median, 95% CrI)
Homotrimer - Apo-homotrimer	−0.0027 (−0.027–0.022)
Heterotrimer - Apo-heterotrimer	−0.0018 (−0.027–0.024)
Homotrimer - Heterotrimer	0.00022 (−0.026–0.026)
Apo-homotrimer - Apo-heterotrimer	0.0014 (−0.024–0.026)

For each contrast, mean RMSD predictions were estimated for the stated trimer types using all draws from the model posterior. The differences in per-draw predictions were calculated and summarized here as median and 95% credible (quantile) intervals.

based on lower Rg values. The holo forms showed marginally lower Rg than the corresponding apo forms, perhaps suggesting a loss of compactness upon calcium depletion. However, for both RMSD and Rg, the sampling error was large, making it difficult to infer any structure-wide differences (Table S1; Figs. S4 *B* and S5). As such, we next examined local residue-level interactions and bonding patterns at the trimer interfaces.

Hydrogen bonding is affected by the loss of calcium

To examine how calcium influences the hydrogen bonding network, inter- and intrachain hydrogen bonds were identified and analyzed using the Cytoscape–Chimera StructureViz package (Figs. 3 and S6; Table S2). In the homotrimer, residues CYS-64 (C3), ASP- 67 and ASN-61 within the calcium-binding loop formed hydrogen bonds with ASP-43, ARG-42, and ARG-39, respectively, on the neighboring chain. This region corresponds to the α-helix containing the partner cysteine (C2) involved in C2–C3 binding. These interactions were conserved between the α1(I) and α2(I) chains.

The α1(I) alpha-helical central region contains an ASP-129 residue that formed a salt bridge with ARG-42 on the neighboring chain as previously described (7). ALA-128 could also form a hydrogen bond with ARG-42, although not as frequently. In the heterotrimer, the α2(I) chain GLU-130 residue formed a salt bridge with ARG-42 on the neighboring chain instead.

These interchain interactions were stabilized by intrachain hydrogen bonds. In the α1(I) chains, the ARG-39–ASN-61 hydrogen bond was reinforced by additional intrachain interactions between ASN-61 and GLN-133, and between ARG-39 and both PRO-60 and GLN-62. The ARG-42 and ASP-67/ASP-129 salt bridges were stabilized by ARG-42 forming hydrogen bonds with LEU-246 and THR-142. ASP-43 also formed an intrachain salt bridge with ARG-39, which may act to stabilize the protein structure and overall interchain hydrogen bonding network, as ARG-39 is one of the residues that participates in binding at the interface. In the heterotrimer, the stabilizing intrachain hydrogen bonds for the α2(I) chain involved ARG-45–PHE-246, ASN-64–GLN-134, and ASP-70–GLN-134 interactions.

In addition to ASP-43 forming an interchain hydrogen bond with CYS-64 and intrachain salt bridge with ARG-39, it also forms an intrachain hydrogen bond with CYS-47 (i.e., it binds both partners in C2-C3 disulfide bond formation). It may be that this residue shuttles between forming hydrogen bonds with one cysteine or the other, coordinating their association during trimer assembly.

To assess whether the hydrogen-bonding networks at the chain interfaces are affected by calcium loss, RMSF values were calculated per residue over each 1000-ns trajectory (Fig. S7) and averaged across the three replicates (Fig. 4).

To capture changes in dynamics that were directly due to calcium loss, RMSF differences between apo and holo states were calculated for both the heterotrimer and homotrimer (Fig. 5) (termed RMSF “hotspots”). Positive values indicated increased flexibility in the apo form relative to the holo form (destabilization); negative values indicated reduced flexibility in the apo form relative to the holo form (stabilization). This revealed an overall destabilization of the α1(I) chains of the heterotrimer and homotrimer without calcium, indicated by positive values.

To visualize the location of the RMSF hotspots, residues with ΔRMSF values >0.05 or <−0.05 were mapped onto the trimer structures (Fig. 6; Table S3). This confirmed that interface regions near the base of the trimers were most perturbed by removal of calcium ions from the structures. Many of the residues that were found to participate in interchain hydrogen bonds or salt bridges were among those destabilized. Interestingly, the exterior face of the α2(I) chain was stabilized in response to calcium depletion in the heterotrimer (Fig. S8). This could reflect the true interface no longer being favorable in the absence of bound ions. A notable stabilization trough was observed in the α1(I) B chain of the homotrimer (Fig. 5); however, this region was largely disordered and appeared to be driven by a single replicate (Fig. S7).

To visualize the dynamic effects of calcium depletion over time, snapshots of the calcium binding region were taken every 200 ns, starting from 0 ns, for both the holo and apo systems, and then the structures were overlaid

shown in red for the heterotrimer structures, and the corresponding α1(I) chain in the homotrimer is shown in a lighter blue. Images are derived from one of three repeat simulations. (*C* and *D*) Time evolution of the root mean-square deviation (RMSD) (*C*) and radius of gyration (Rg) (*D*) over 1000 ns. Shaded regions represent ±1 standard error from block-averaged data across three replicate simulations, with smoothed mean trajectories overlaid. (*E* and *F*) Individual replicate trajectories for RMSD (*E*) and Rg (*F*) for each trimer system (homotrimer, apo-homotrimer, heterotrimer, apo-heterotrimer). Each replicate (termed “R1,” “R2,” and “R3”) is shown as a separate trace in blue, red, and yellow.

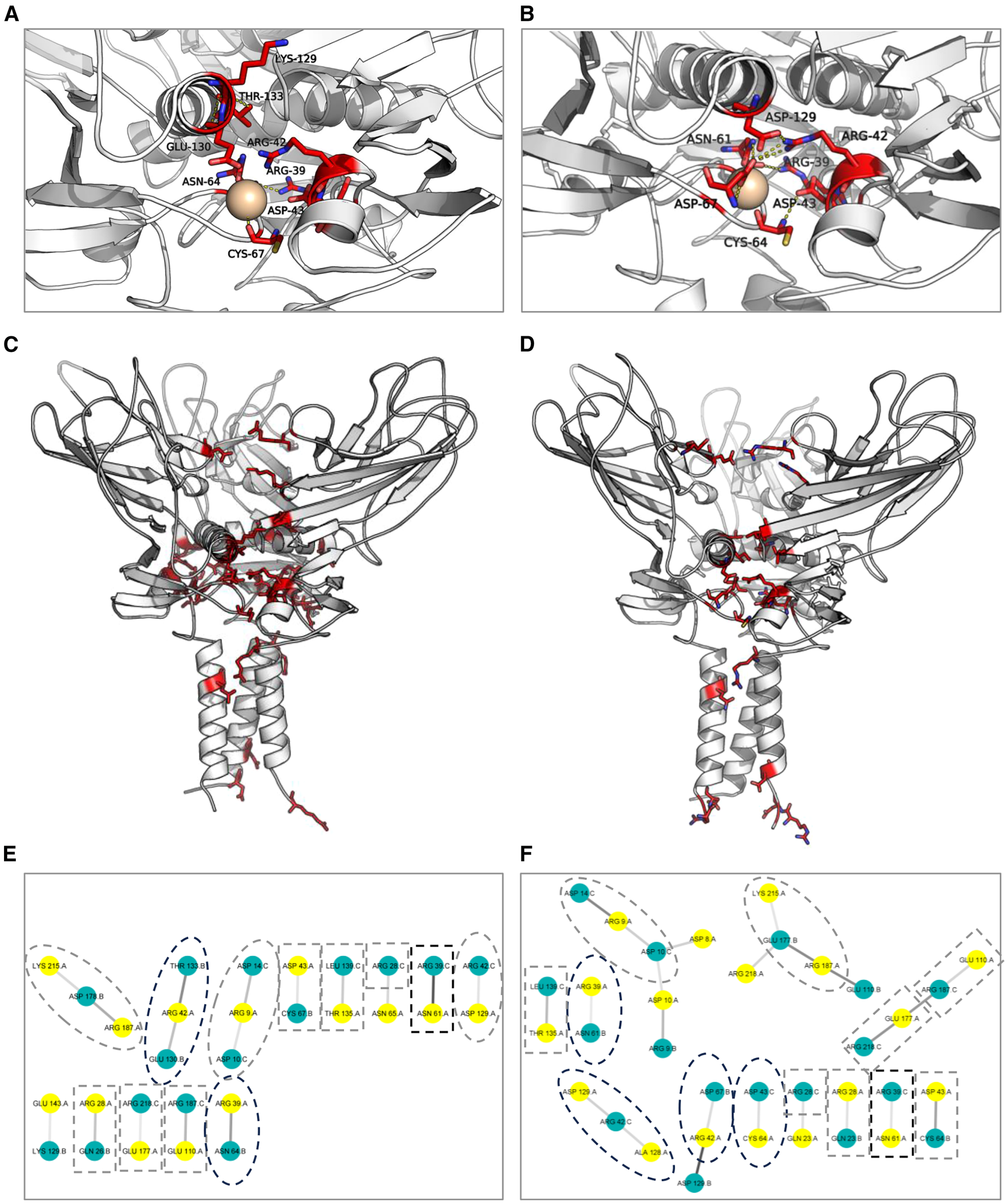

FIGURE 3 Hydrogen bonding networks in C-propeptide heterotrimers and homotrimers revealed by StructureViz analysis. (*A–D*) Trimer cartoons with residues that participate in interchain hydrogen bonds shown in red and as "stick" representations. (*A* and *B*) Hydrogen bonds at the chain interface for the heterotrimer (*A*) and homotrimer (*B*). (*C* and *D*) Interchain hydrogen bonds for the whole structure for the heterotrimer (*C*) and homotrimer (*D*). The calcium ion is shown as a wheat-colored sphere. The N-terminal regions demonstrate increased flexibility due to the lack of Gly-X-Y domain. (*E* and *F*) Hydrogen bonding network for the α1(I) chain A from explicit hydrogen MD simulations for the heterotrimer (*E*) and homotrimer (*F*). Only one replicate was carried out with explicit hydrogens, with a time step of 2 fs. α1(I) chain A is shown in yellow; chain B, i.e., α1(I) in the homotrimer and α2(I) in the heterotrimer, is shown in teal; α1(I) chain C is also shown in teal. The edge weight corresponds to how conserved the bond was throughout the simulation. The darker edges represent bonds that were present throughout most of the simulation; the lighter ones were more transient bonds. Black circles denote bonds referred to in the text. Gray circles denote bonds that are conserved, or similar, between the heterotrimer and homotrimer.

(Fig. 7). In both trimers, intrinsically disordered regions became more mobile upon calcium loss, but the calcium-binding loop was most obviously affected. In the holoproteins (Figs 7, A and C), the calcium-binding loops maintained a consistent orientation, remaining close to the neighboring chain. In contrast, in the apo systems the loop exhibited increased conformational flexibility, moving inward and away from the interface. The apo-homotrimer

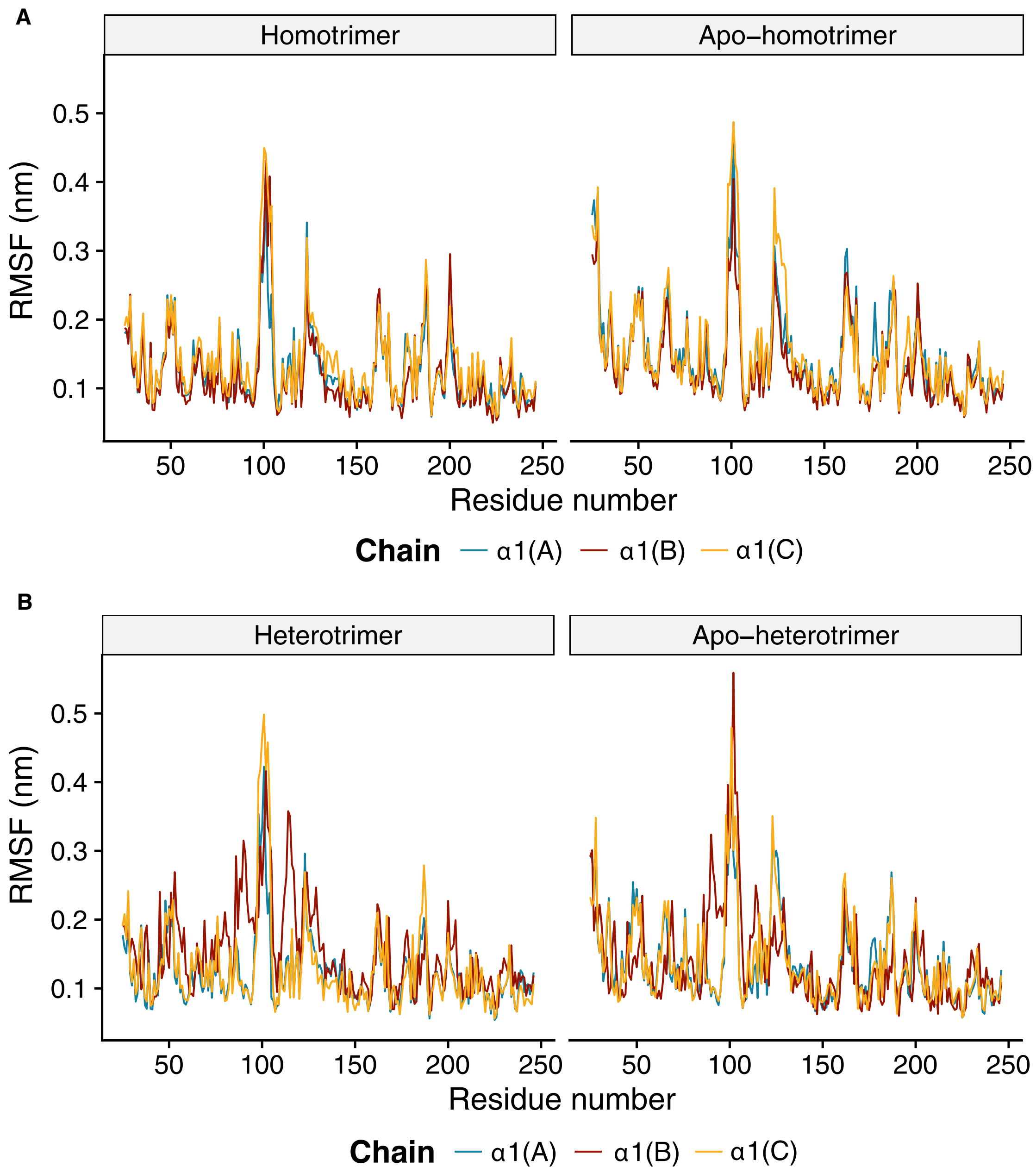

FIGURE 4 The RMSF of the backbone as a function of amino acids. (*A*) Holo-homotrimer and apo-heterotrimer. (*B*) Holo-heterotrimer and apo-heterotrimer. Each trimer has three chains: chains A, B, and C. In the homotrimer, all three chains are α1(I). In the heterotrimer chains A and C are α1(I) and chain B is α2(I). Each chain is colored separately per trimer: blue (chain A), red (chain B), and yellow (chain C).

also developed a minor fold at later time points (Figs 7, B and D). The calcium-binding loop appeared more stable in the holo-heterotrimer than the holo-homotrimer.

Together, the StructureViz analysis, RMSF values, and trajectory visualization demonstrate a role for structural calcium in coordinating hydrogen bond and salt bridge formation at the interface between chains. In the absence of calcium, these interface regions display increased conformational flexibility, which is likely to impair trimer assembly. Notably, these interface loops also contain the disulfide-forming cysteines, which next prompted the investigation of calcium coordination and cysteine pairing.

Calcium maintains sufficient proximity for interchain disulfide bonding

It is known that after the individual α-chains associate, they become irreversibly disulfide bonded by protein disulfide isomerase (PDI). The cysteine code ensures the proper assembly of homotrimers and heterotrimers and prevents

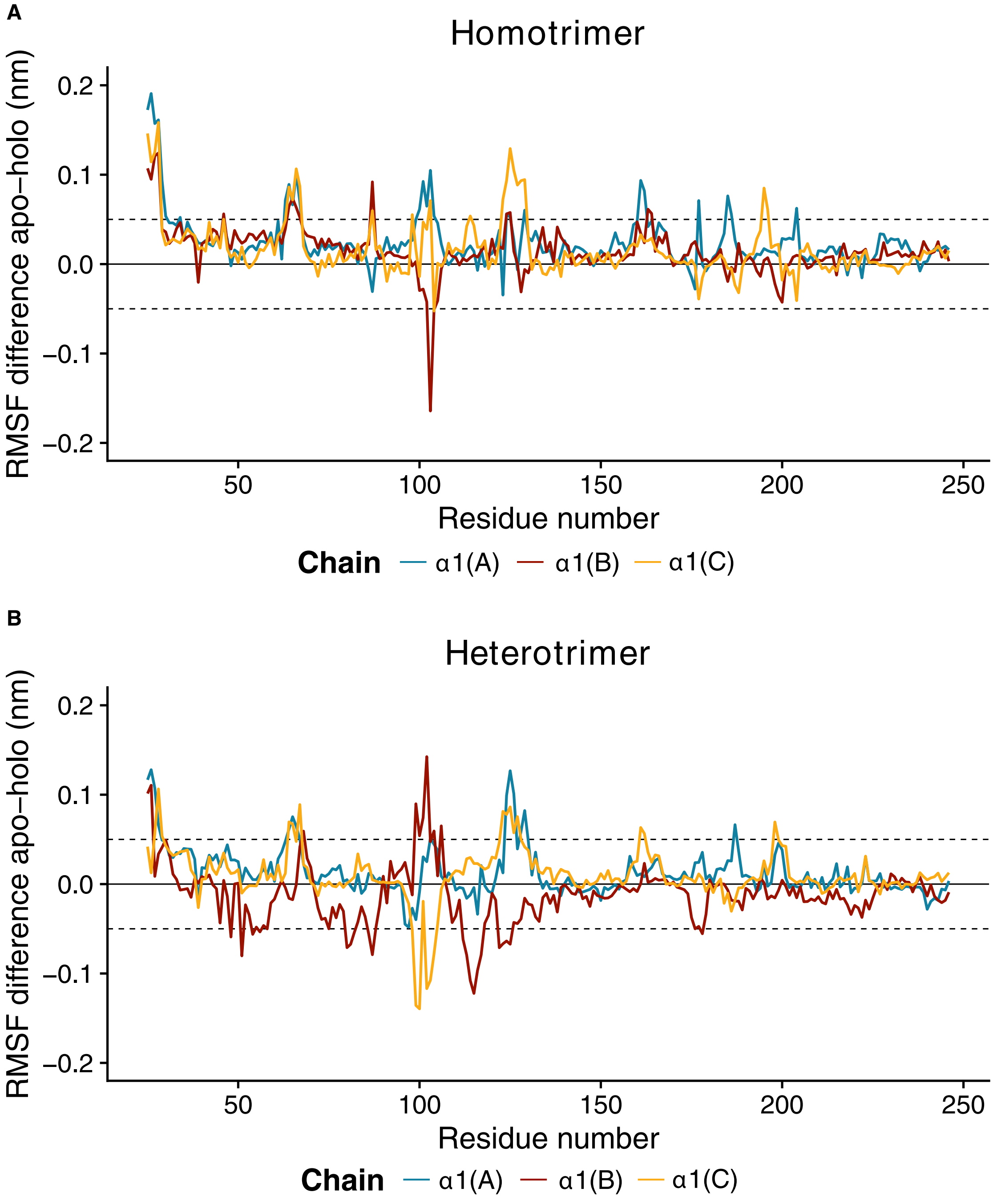

FIGURE 5 Per-residue RMSF differences between holo- and apo-trimers. The RMSF values for the apo-trimers were subtracted from the holo-trimers and the difference was then plotted per chain for the homotrimers (*A*) and heterotrimers (*B*).

α2(I) homotrimers from forming interchain disulfide bonds. In the absence of calcium, no trimers form; only monomers and short-lived dimers are present (20). The relevant cysteines and the α2(I) serine are visualized in Figs. 8, *A* and *B*.

To determine the impact loss of calcium has on covalent bond formation, the distance between the Cα atoms of C2 and C3 cysteines, and the α2(I) serine to C3 cysteine for the heterotrimer, at each chain interface was measured (Fig. 8 *C*). Disulfide bonds can form between cysteines at distances of 0.3–0.75 nm (47,48). After the disulfide bond has formed the linkage is typically about 2.04 Å (0.2 nm) in length. At distances greater than 0.8 nm, disulfide bonds are unlikely to be observed (47). For the holo-trimers, the distance between the C2 and C3 cysteines remained near constant through the equilibrium simulations, fluctuating between 0.6 and 0.8 nm (Figs. 8 *C*, S9, and S10), with only modest differences in mean distance observed between the holo-trimers at chain

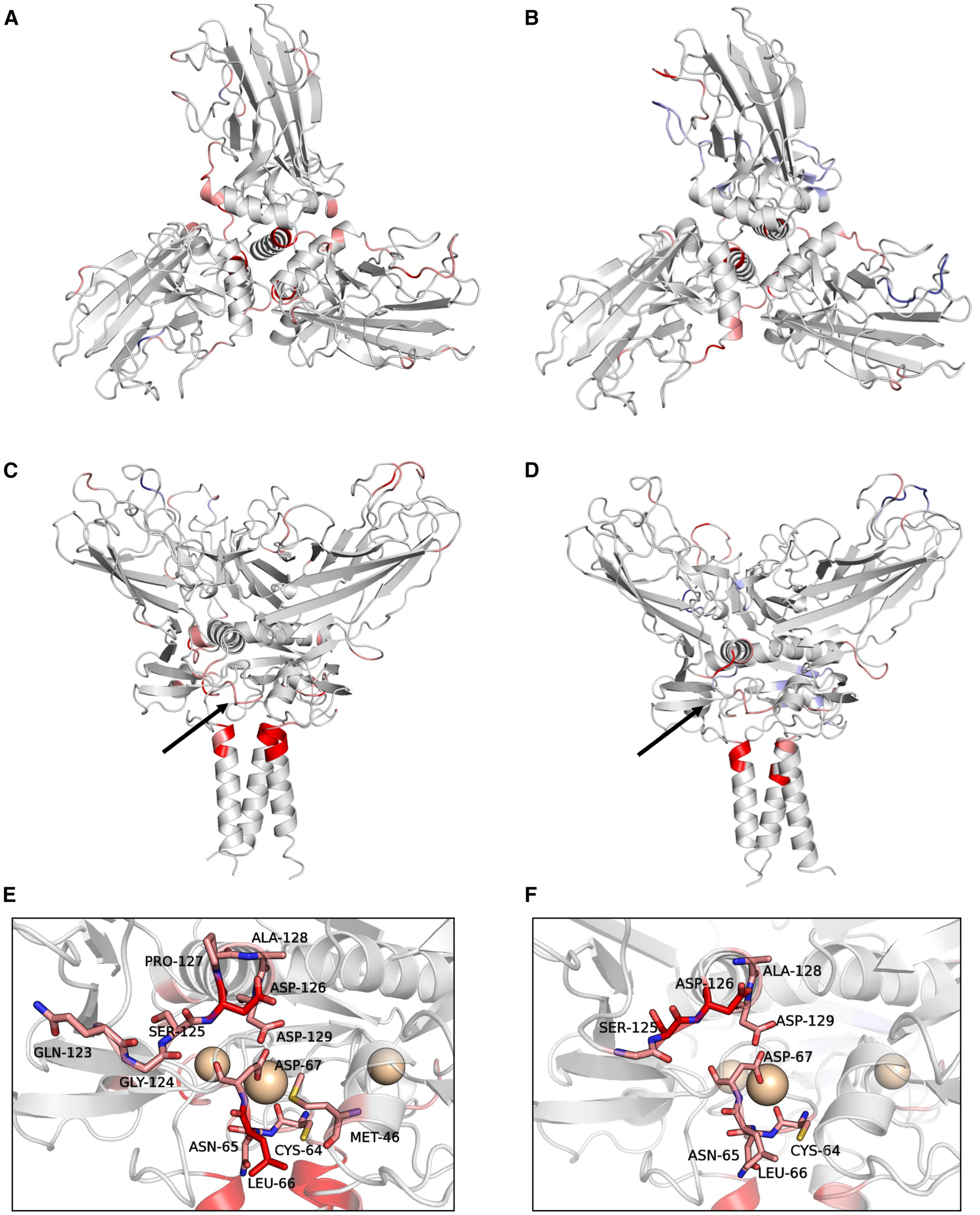

FIGURE 6 RMSF hotspots in the heterotrimer and homotrimer. (*A* and *B*) Top-down view of the homotrimer (*A*) and heterotrimer (*B*). (*C* and *D*) Side-on view of the homotrimer (*C*) and heterotrimer (*D*). Left chain is an α1(I) chain, and right chain is an α1(I) B chain in the homotrimer or an α2(I) B chain in the heterotrimer. (*E* and *F*) Zoomed view of calcium-binding region at the interface of the homotrimer (*E*) and heterotrimer (*F*). For all panels, RMSF hotspots (defined as residues with ΔRMSF values >0.05 or <-0.05) are colored in. Red: ΔRMSF >0.1; salmon: ΔRMSF 0.05–0.1; light blue: ΔRMSF -0.05 – 0.1; dark blue: ΔRMSF <-0.1. Calcium ions are shown as wheat-colored spheres.

interfaces B and C (Fig. S11; Table S4). In contrast, for the apo-trimers, the C2-C3 distance increased over the course of the simulation, with average values generally greater than 0.9 nm (Fig. S10). Consistent with this observation, mean distances were greater for apo-trimers than their respective holo-trimers across all interfaces (Fig. S11; Table S4). Hence, simulations indicate that structural calcium in the trimers maintains a suitable distance between the C2 and C3 cysteines for covalent disulfide bonds to form.

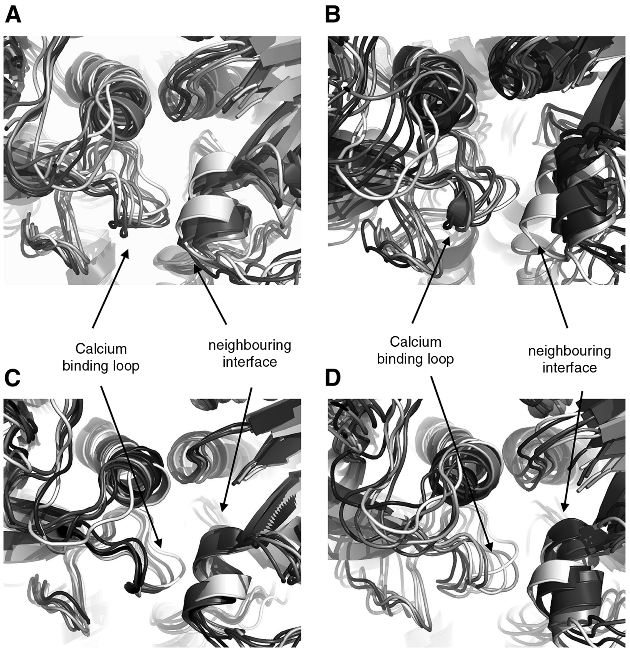

FIGURE 7 Time evolution of the calcium-binding region in homotrimers and heterotrimers. (*A*) Holo-homotrimer (*B*) Apo-homotrimer. (*C*) Holo-heterotrimer. (*D*) Apo-heterotrimer. Snapshots were taken every 200 ns from 0 to 1000 ns and overlaid. The time points are colored from the earliest time point in the lightest shade (*white*) to the latest time point in the darkest shade (*black*). Calcium ions are omitted from the holo structures for visual clarity.

Pulling simulations and τRAMD demonstrate that calcium is more strongly bound to α1(I) than the α2(I) chain

The calcium ion is coordinated by three conserved residues in the calcium binding loop: ASP-59, ASN-61, and ASP-67 in the α1(I) chain and ASP-62, ASN-64 and ASP-70 in the α2(I) chain. The calcium binding loops share 83% sequence identity and vary by only two residues. ASN-65 and LEU-66 in the α1(I) chain are replaced by THR-68 and MET-69 in the α2(I) chain. To compare calcium binding strength, COM pulling simulations and τ-random accelerated molecular dynamics (τRAMD) were performed (Fig. 9).

After a short 10-ns equilibration, COM pulling simulations (SMD) were carried out to extract the calcium ion from its binding site (Fig. S12) (49). The resulting force-time curves for the dissociation of calcium from each chain showed an approximately twofold difference in the rupture force (F_{max}) required to separate the calcium ion from its binding site in the α1(I) chain, compared with the α2(I) chain (Figs 9 *A* and S13). The force profiles demonstrated a three-step unbinding pattern, appearing to correspond to the sequential loss of the three coordinating residues.

Umbrella sampling was employed to compute the PMF along the unbinding coordinate (Fig. S14). Both α1(I) and α2(I) unbinding profiles featured a single intermediate (PL*), consistent with a two-state release mechanism. The bootstrapped standard deviation of the PMF was narrow (<0.5 kcal mol^{-1}) in the bound region and widened slightly upon ion release (Fig. S14 *A*). The α1(I) chain exhibited a deeper binding well (ΔG_{min} = – 4.99 kcal mol^{-1}) than α2(I) (ΔG_{min} = – 3.52 kcal mol^{-1}) (Table S5), suggesting a more stable bound state; however, there was only a modest change in Gibbs free energy, ΔG (50). The similar free energies suggested the α1(I) chain and α2(I) chains have comparable binding affinity for the calcium ion, in contrast with the SMD, which had higher rupture forces for α1(I). This could reflect the one-dimensional reaction coordinate being insufficient to capture all the barriers arising from coordination and local gating, and additionally, the histograms showed some limited sampling at the start of the reaction coordinate, despite the narrow windows (Fig. S14B).

τRAMD simulations were employed to cross-validate the pulling results and to quantify relative residence times (τ) of calcium in each chain (Figs 9 *B* and S15). Each trajectory was tested for distributional normality using the Kolmogorov-Smirnov (KS) test (Table 2; Figs. S15, *E* and *F*). Across replicates, calcium residence times were significantly longer in α1(I) (mean = 1.54 ± 0.22 ns) than in α2(I) (mean = 0.63 ± 0.06 ns), corresponding to a ∼2.4-fold difference (p = 0.014; Table 3). The slower dissociation kinetics in α1(I), coupled with the deeper PMF well and higher rupture forces, suggest that although both loops have similar overall thermodynamic affinity, calcium is mechanically and kinetically more tightly bound to α1(I).

Enhanced sampling methods suggest that the α2(I) chain has a higher trimer affinity than a third α1(I) chain in the presence of structural calcium

To investigate the energetic basis for preferential heterotrimer formation, a combination of SMD, umbrella sampling, interchain interaction enthalpy (INTAA) analysis, and thermodynamic integration calculations was employed. These complementary methods were used to evaluate both the mechanical and thermodynamic stability of homotrimeric and heterotrimeric type I collagen C-propeptide assemblies for both the holo and apo states.

The force-time profiles generated from SMD (Fig. S16) suggested that the holo-heterotrimer exhibited the highest rupture force, followed by the holo-homotrimer, whereas both apo systems were considerably weaker (Fig. 10 *A*). The heterotrimer displayed a biphasic unbinding process, corresponding to an initial rupture followed by a smaller secondary event that was mirrored in the number of interchain hydrogen bonds.

Umbrella sampling analyzed using the WHAM produced PMF profiles for chain dissociation (Fig. 10 *B*). Histogram overlap confirmed sufficient sampling (Fig. S17). The PMFs suggested the holo-heterotrimer had the highest binding affinity (ΔG) −159.4 kJ mol^{-1}, followed by the

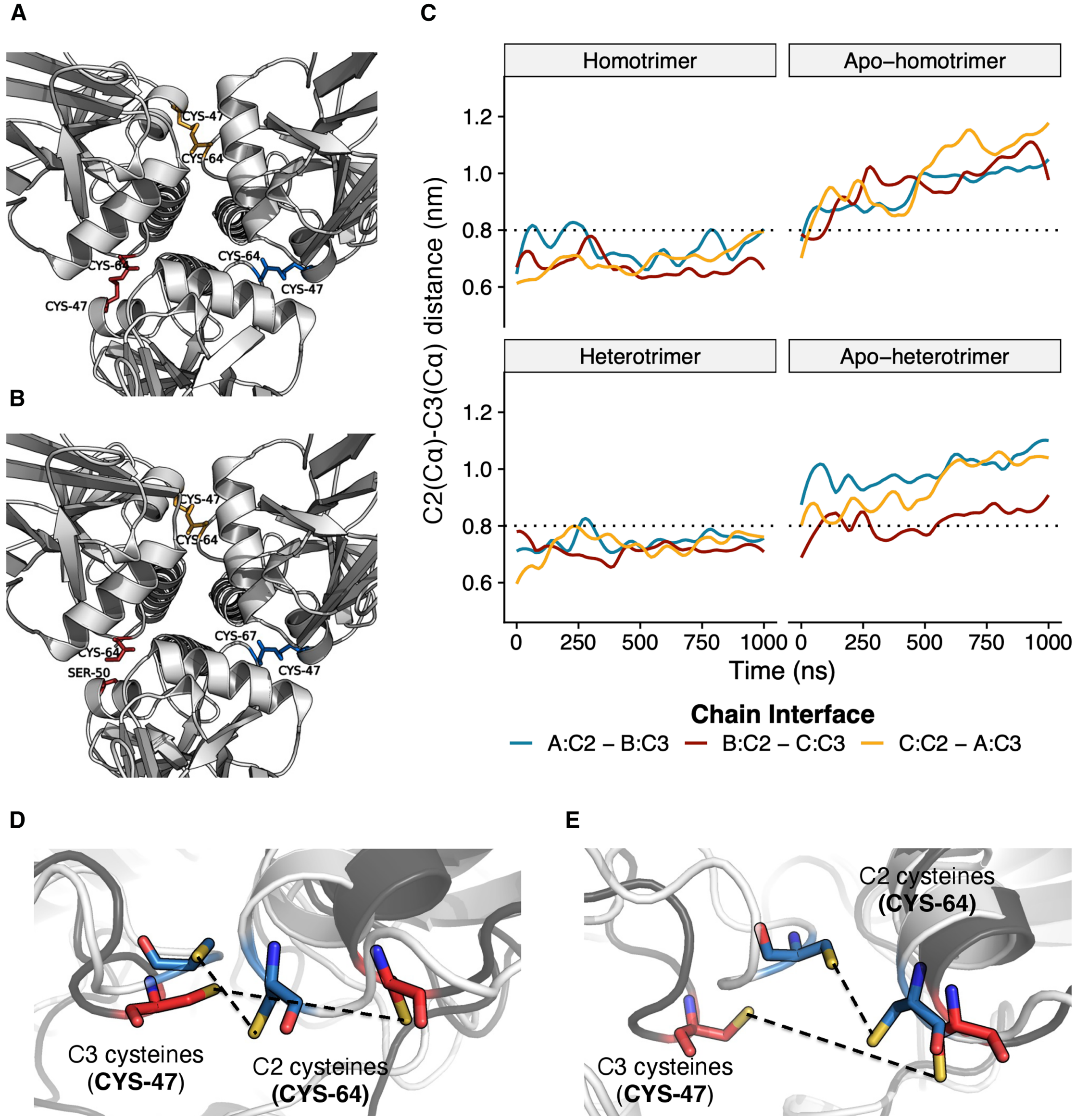

FIGURE 8 Interchain disulfide bonds and simulated interresidue distances for the C-propeptide heterotrimer and homotrimer. (*A* and *B*) Top-down view of the interchain disulfide bonding cysteines for the homotrimer (*A*) and heterotrimer (*B*). For the heterotrimer (*B*), the α2(I):α1(I) chain interface (chain B:chain C) (*red*) does not contain a disulfide bond, due to the Cys-Ser substitution in the α2(1) chain. The bonds are shown as sticks. The remainder of the chains are shown in light gray as cartoon representations. (*C*) Smoothed average distance between the Cα atoms of the interchain disulfide forming residues over the course of the three equilibrium simulation replicates. Each panel shows a different trimer, in order: the homotrimer, apo-homotrimer, heterotrimer, apo-heterotrimer. Each trimer has three chains: chains A, B, and C. In the homotrimer, all three chains are α1(I). In the heterotrimer, chains A and C are α1(I), and chain B is α2(I). The chain A-B interface C2-C3 distance is shown in blue, the chain B-C interface C2-C3 distance is shown in red, and the chain C-A interface C2-C3 distance is shown in yellow. In the heterotrimer the red interface is the distance between the equivalent SER-50 Cα and CYS-64 Cα (C3). (*D* and *E*) Zoomed-in view of the C2-C3 cysteines off the homotrimer (*D*) and heterotrimer (*E*) α1 chain A and α1 chain C (*yellow interface*). The cysteines are shown as stick representations. Blue: 0 ns; red: 1000 ns. Distances between the sulfhydryl groups are shown as dotted lines.

holo-homotrimer (−104.5 kJ mol^{-1}), whereas the apo forms were markedly weaker (−66.1 and −62.7 kJ mol^{-1}, respectively) (Table 4).

However, the one-dimensional coordinate used in the umbrella sampling does not fully represent the conformational rearrangements accompanying trimer association or dissociation. Consequently, the PMFs reflect relative dissociation barriers rather than absolute binding energies. To provide a more comprehensive energetic assessment, additional enthalpic and free energy analyses were undertaken. Interchain interaction enthalpies calculated using the INTAA method showed that the calcium-bound heterotrimer possessed stronger total interchain interactions (−255.7 kJ mol^{-1}) than the calcium-bound homotrimer (−234.6 kJ mol^{-1}), a difference of −21.1 kJ mol^{-1} (Fig. 10 *C*). The greatest difference in stabilization occurred at the

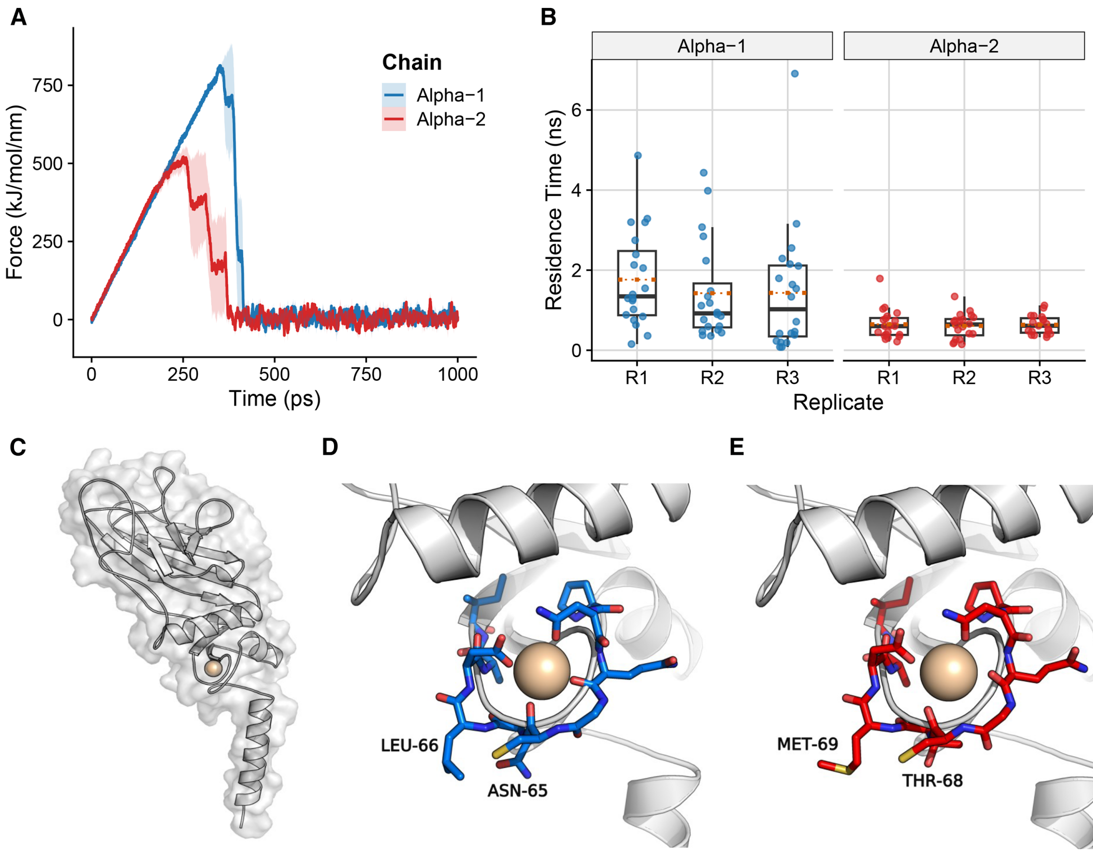

FIGURE 9 Steered molecular dynamics simulations and τRAMD simulations of calcium uncoupling from C-propeptide monomers. (*A*) Force-time curves derived using default Amber ff99SB-ILDN parameters. The darker lines are the average values over five replicates. The shaded areas are the error (standard deviation). (*B*) The residence times for three replicas for the α1(I) and α2(I) chain. Boxplots summarise the τRAMD results (25 independent trajectories per replicate). Boxes represent the interquartile range (IQR; 25th to 75th percentiles), whiskers extend to the most extreme values with 1.5 times the interquartile range. Individual points correspond to residence times from single τRAMD trajectories. The median value is shown as a black line and the mean value by an orange dotted line. (*C*) Surface representation of an α1(I) monomer, demonstrating that calcium is exposed to solvent. (*D* and *E*) Differences between the α1(I) (*D*) and α2(I) (*E*) calcium-binding loops. Calcium ions are shown as spheres.

α2(I)-α1(I) (B-C) interface (−14.9 kJ mol^{-1}). The additional stability was primarily attributed to hydrophobic contacts between MET-69 (from LEU-66 to MET) and MET-46, and favorable interactions near the C2-C3 disulfide bridge. THR-68 (from ASN-65 to THR) contributed additional solvent-exposed hydrogen bonding, improving the local electrostatic environment.

Alchemical relative free energy perturbation calculations quantify heterotrimer stabilization

To quantify the thermodynamic contribution of α2(I)-specific residues to trimer stability, thermodynamic integration simulations were performed. Two interface substitutions characteristic of the α2(I) chain (ASN-65 to THR and LEU-66 to MET) were evaluated individually and in combination in both the apo and holo states. Each alchemical transformation was propagated for 200 ns under identical force field and solvation conditions to ensure direct comparability.

Relative binding free energies (ΔΔGbind) were derived from paired simulations of the trimeric complex ("complex route") and corresponding monomeric chains ("solvent route"), with calcium dependence calculated as $\Delta\Delta\Delta GCa^{2+}$ = ΔΔGbind(holo) – ΔΔGbind(apo). In the holo systems, the combined mutations stabilized the heterotrimer relative to the homotrimer by ΔΔGbind = −5.6 kJ mol^{-1}, whereas in the apo systems the difference was negligible (−0.9 kJ mol^{-1}), resulting in a calcium-dependent stabilization of $\Delta\Delta\Delta GCa^{2+}$ = −4.7 kJ mol^{-1} (Table 5). At the simulation temperature of 300 K, this ΔΔGbind(holo) corresponds to approximately 2.2 RT units and an equilibrium constant (Krel) of approximately 9.5, indicating a 9.5-fold energetic preference for heterotrimer formation (51). This energetic preference is essentially eliminated under calcium depletion (ΔΔGbind = −0.9 kJ mol^{-1}, Krel ≈1.4), demonstrating the role of calcium in biasing trimer composition. The calcium-dependent difference ($\Delta\Delta\Delta GCa^{2+}$ = −4.7 kJ mol^{-1}, approximately 1.9 RT units) represents a 6.6-fold enhancement of heterotrimer preference upon

TABLE 2 Residence Times, Relative k_{off} Values, and Kolmogorov-Smirnov Test Results Calculated from τRAMD Simulations

Chain	Replicate	Mean Relative Residence Time (ns)	Standard Deviation (ns)	KS Test
α1(I)	1	1.76	0.14	0.2
α1(I)	2	1.42	0.09	0.15
α1(I)	3	1.43	0.36	0.16
α2(I)	1	0.65	0.06	0.26
α2(I)	2	0.61	0.05	0.18
α2(I)	3	0.64	0.06	0.39

TABLE 3 Mean Residence Times and Relative k_{off} Values Calculated from τRAMD Simulations

Monomer	Mean Residence Time (ns)	Standard Deviation	Relative k_{off} (ns^{-1})
α1(I)	1.54	0.22	0.65
α2(I)	0.63	0.06	1.59

calcium binding. This moderate but consistent energy difference (52) indicates that the α2(I)-specific residues confer a measurable thermodynamic advantage to trimer formation in the presence of calcium, which complements the structural constraints imposed by the cysteine code.

DISCUSSION

In the present study we have uncovered a calcium-dependent mechanism that determines whether type I collagen forms heterotrimers or homotrimers. Our approach, integrating multiple simulation approaches and analyses, reveals a hierarchical series of calcium-mediated effects: calcium binding stabilizes the hydrogen bonding network at chain interfaces, maintaining the proximity required for disulfide bond formation between cysteine residues; α1(I) chains bind calcium approximately twice as strongly as α2(I) chains; and α2(I)-specific residues confer a 9.5-fold thermodynamic advantage for heterotrimerization, which is essentially eliminated under calcium depletion. We propose a model whereby reduced calcium concentration in the endoplasmic reticulum would favor homotrimerization over heterotrimerization by allowing α1(I) chains to preferentially sequester available calcium ions (Fig. 11).

Previous studies have shown that the homotrimer will form in the absence of α2(I) chains due to genetic inactivation or epigenetic silencing of *COL1A2* (14,53,54) or by overproduction of the α1(I) chain, either artificially or due to a common *COL1A1* polymorphism (55,56). However gene dosage and the relative abundances of *COL1A1* and *COL1A2* mRNAs do not directly predict heterotrimerization versus homotrimerization (57,58), likely because *COL1* mRNA translation is coordinated by numerous cytosolic factors (59), and the α2(I) chain sequence itself favors heterotrimerization.

Collagens are co-translationally translocated into the lumen of the rough ER during biosynthesis. The entire chain must be translated before the C-propeptide can fold and mediate trimerization. Normal free ER calcium concentrations range from 0.5–2.0 mM in many different cell types. Calcium pumps and channels maintain calcium homeostasis (60). Calcium availability in the ER is buffered by calcium-binding proteins, primarily calsequestrin in skeletal and cardiac muscle, and calrecticulin in other tissues. Calcium-dependent collagen chaperones such as BiP/GRP78, GRP94, PDI, and calnexin also act as calcium stores (61). Previous sedimentation equilibrium experiments with recombinant C-propeptides lacking cysteines showed that homotrimerization occurred in 0.5 mM calcium, but that monomers predominated in the absence of calcium (20). Future studies with mixtures of α1(I) and α2(I) chains over a range of calcium concentrations could elucidate the minimum calcium concentration for heterotrimerization.

Chronic decreases in ER calcium concentrations can affect the unfolded protein response, leading to misfolded proteins and apoptosis (61). The importance of intracellular calcium homeostasis is demonstrated by the ER stress and dysregulated type I collagen synthesis caused by loss of *TMEM38B*/TRIC-B, an ER membrane cation channel, which results in osteogenesis imperfecta (OI) (62). However, ER calcium concentrations are decreased in many pathological states including diabetes, ischemia, cardiovascular disease, viral infections, asthma, liver disease, and cancer (61). Calcium homeostasis also becomes dysregulated in aging due to oxidative damage, decreased expression of the ER calcium ion pump SERCA, and alterations in calcium-sensing proteins (63–65). Hence age- and disease-associated decreases in ER calcium could lead to homotrimer formation. Chronic diseases can be associated with increased focal collagen production, and the corresponding production of the homotrimeric form could accelerate fibrosis, due to the increased resistance of the homotrimer to MMP-mediated turnover (15).

Fibroblasts characteristically produce abundant type I collagen, particularly during development and tissue homeostasis. In fibroblasts, calcium signaling is mechanosensitive and responds to the cell's three-dimensional environment (66), which is especially relevant for proliferation and migration of fibroblasts during wound healing and leads to cyclic fluctuations in the amount of free calcium available (67). Hence the chain-specific trimerization of type I collagen may be influenced by the mechanical environment; we previously noted evidence of type I collagen homotrimer synthesis in precontracted but not fully contracted tendon-like constructs (58).

In two diseases of the skin, Darier disease and Hailey-Hailey disease, mutations in the genes for the ER calcium ion pumps SERCA2 and SPCA1, respectively, lead to dysregulation of calcium homeostasis and phenotypic features such as the formation of warts, lesions, blisters, and malodorous plaques (68). It is unknown if homotrimeric

TABLE 4 Apparent Binding Free Energy (ΔG_app) Derived from PMF Dissociation Profiles

Trimer	ΔG
Homotrimer	−104.52
Heterotrimer	−159.40
Apo-homotrimer	−66.13
Apo-heterotrimer	−62.70

Values represent relative association strength rather than absolute binding energies due to the one-dimensional reaction coordinate used. Values were derived from chain B, either an α1(I) or α2(I), uncoupling from chains A and C, which were both α1(I). Simulations were carried out in apo- and holo-type I collagen trimer isoforms.

type I collagen is present in these conditions, though a *COL1A1-PDGFB* fusion gene is implicated in similar skin conditions (69).

Type I collagen homotrimers promote metastasis and invasion in cancer cells by providing MMP-resistant pathways for cell migration (8,54). ER calcium concentrations are often decreased in cancer cells, creating apoptosis resistance (70). It may therefore be that this depleted ER calcium contributes to homotrimerization of type I collagen during malignant transformation.

Our reported changes in hydrogen bond formation and salt bridge formation also demonstrate how known type I collagen C-propeptide mutations can cause disease. Approximately 6.5% of OI patients have mutations in the C-propeptide. The OI-causing mutation P-1182-R (at position 63 in the α2(I) C-propeptide) is proximal to the ASN-64 residue (71,72), which is important in stabilizing the binding interface between chains. Replacing the

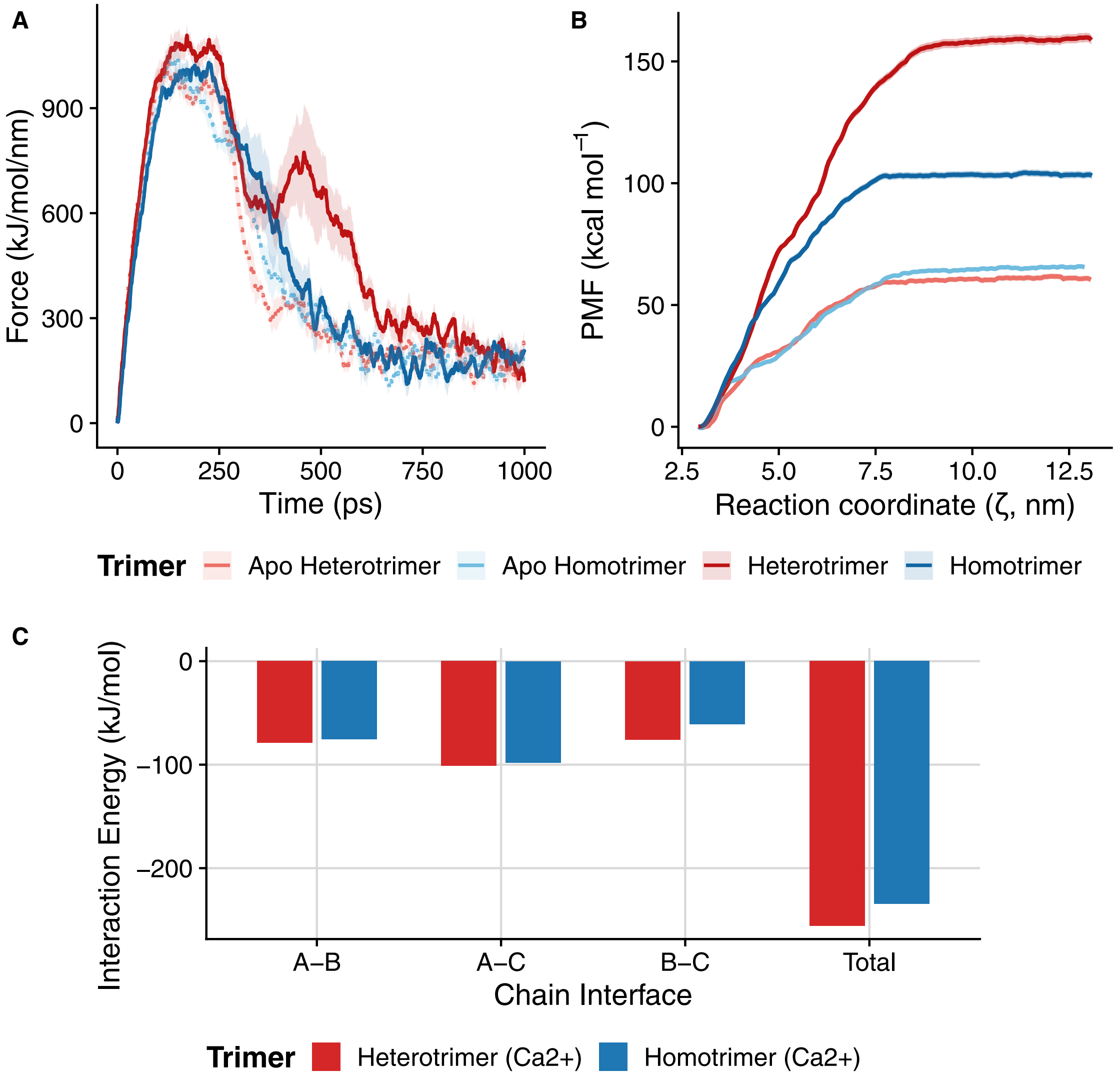

FIGURE 10 Steered molecular dynamics simulations of the α2(I) or corresponding α1(I) chain pulled from C-propeptide heterotrimers or homotrimers and interchain interaction enthalpies. (*A*) Force-time curves of type I collagen trimers with and without calcium bound (holo and apo). The darker lines are the average values over 5 replicates. The shaded areas are the error (standard deviation). (*B*) Potential of mean force (PMF) curves for: heterotrimer, apo-heterotrimer, homotrimer, and apo-homotrimer, obtained via weighted histogram analysis (WHAM). The PMF is shown as a solid line, and the shaded regions denote the bootstrapped ±1 SD uncertainties (100 resamples). (*C*) Summary of interchain interaction enthalpy (INTAA) calculations for each trimer at each interface.

TABLE 5 Summary of FEP Calculations for Alchemical Thermodynamic Integration Energy Perturbation $\Delta\Delta G_{bind} = \Delta G_{trimer} - \Delta G_{monomer}$

Mutations	Ca^{2+} Status kJ/mol	ΔG_Trimer kJ/mol	ΔG_Monomer kJ/mol	ΔΔG_bind kJ/mol kJ/mol	ΔΔΔG_Ca^{2+} kJ/mol
ASN-65→THR	Apo	248.7	249.1	−0.4	–
ASN-65→THR	Holo	244.3	257.4	−13.1	–
LEU-66→MET	Apo	77.7	78.2	−0.5	–
LEU-66→MET	Holo	79.7	72.2	7.5	–
Sum of both mutations	Apo	326.4	327.3	**−0.9**	–
Sum of both mutations	Holo	324.0	329.6	**−5.6**	–
Holo − Apo	–	–	–	–	**−4.7**

Values in bold were used to calculate the equilibrium constant (Krel)

neighboring small, neutrally charged proline with a bulky, positively charged arginine likely interferes with hydrogen bond formation and chain incorporation. The same principle applies for other OI-causing mutations. THR-1431 (located at position 213 in the α1(I) C-propeptide) is close to the ARG-218 residue that participates in hydrogen bonding with GLU-177 in the neighboring chain. TYR-1263 (located at position 144 in the α2(I) C-propeptide) (71) has two neighboring residues that participate in inter- and intrachain hydrogen bonding respectively. It may also be that these mutations interfere with the solvent accessible area or directly repel their neighbors.

While the combination of equilibrium and nonequilibrium simulations, alchemical free energy calculations, and enhanced sampling techniques employed in this work provide strong computational evidence for calcium-mediated control of trimerization, several avenues for experimental validation and extension remain. First, sedimentation equilibrium or isothermal titration calorimetry experiments with mixtures of recombinant α1(I) and α2(I) C-propeptides across a range of calcium concentrations could test our predictions of the calcium threshold for heterotrimerization and the predicted 9.5-fold preference. Second, cellular studies monitoring trimer composition in response to controlled ER calcium depletion (using thapsigargin or

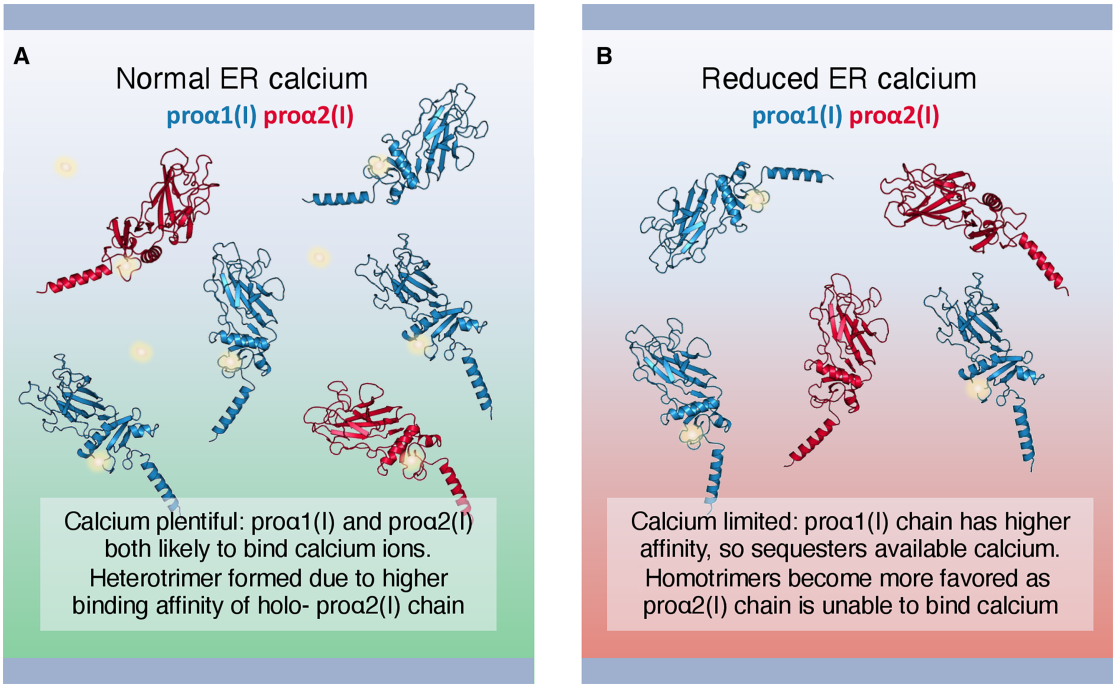

FIGURE 11 Proposed model for calcium regulation of type I collagen hetero- versus homotrimerization. (*A*) Heterotrimers are energetically favored in normal ER calcium concentrations, where there is sufficient calcium to bind all proα1(I) and proα2(I) monomers. (*B*) Under calcium-depleted conditions, energetic preference for heterotrimers is lost, and if proα1(I) chains preferentially sequester available calcium due to their longer residence times, homotrimer formation may be favored. This model is supported by free energy calculations, calcium residence times, and structural dynamics simulations. For clarity, only the C-propeptides of the procollagen proα1(I) and proα2(I) chains are shown; triple-helical regions and N-propeptides are omitted. Other ER chaperones required for procollagen folding are not shown. The proα1(I) C-propeptide monomers are shown in blue and the proα2(I) monomers in red. Calcium ions are depicted as light-yellow spheres.

ionomycin) (73–75) would validate the physiological relevance of our model. Third, the simulations presented here focus on the C-propeptide; inclusion of the full-length triple helical domain (21) (or partial inclusion of the sequences near the C-terminus) in future studies could reveal how the differential stability of α1(I) and α2(I) helices further modulates calcium-dependent assembly. The α2(I) helix is less stable than α1(I), which could amplify the effects of calcium depletion on heterotrimerization. The stability of this region could be explored using the computational approaches we've used herein, adding to our understanding the multiple mechanisms governing type I trimerization. Finally, the one-dimensional distance coordinate used to generate the PMF profiles cannot fully capture the conformational complexity of trimer assembly. PMFs have been previously used to study tetramers with some caveats (76). Here, we interpreted the PMF profiles as representing dissociation barriers rather than absolute binding free energies, and thus, they should only be treated as relative comparisons. In future work, coarse-grained approaches could be used to better sample the collective variable (77).

Nevertheless, the consistency across our multiple independent computational approaches—equilibrium MD, steered MD, τRAMD, umbrella sampling, and thermodynamic integration—all demonstrating calcium-dependent preferential stabilization of heterotrimers, provides strong convergent evidence for the calcium-dependent mechanism proposed here and strengthens confidence that conclusions reflect genuine physical phenomena. Most critically, alchemical thermodynamic integration provides rigorous, pathway-independent quantification of α2(I)-specific contributions to binding. Furthermore, the relative nature of our comparisons (heterotrimer versus homotrimer; α1(I) versus α2(I)) ensures systematic errors largely cancel when computing differences.

From a translational perspective, our findings suggest that therapeutic interventions targeting ER calcium homeostasis might prevent or reduce pathological homotrimer formation. Calcium channel modulators, SERCA pump activators, or agents that enhance ER calcium buffering capacity could shift the equilibrium back toward heterotrimerization in disease contexts. Conversely, understanding the conditions favoring homotrimerization could inform tissue engineering applications where the distinct mechanical properties of homotrimeric collagen might be deliberately exploited. Further investigation of the relationship between calcium concentration and trimer composition in pathological tissues would be valuable for developing such targeted therapeutic strategies.

DATA AND CODE AVAILABILITY

The data can be obtained from Emily J. Johnson (emily.johnson@liverpool.ac.uk) or from the corresponding author on request. Code is provided in the following link: https://github.com/CBFLivUni/EJohnson_calcium_collagen_trimerisation.

ACKNOWLEDGMENTS

This work made use of the Barkla High Performance Computing facilities at the University of Liverpool. The authors would also like to thank Professor Dan Rigden for his advice and support during the revisions of this paper. Finally, the authors acknowledge use of the Computational Biology Facility provided by Liverpool Shared Research Facilities, University of Liverpool.

This work was funded by the Biotechnology and Biological Sciences Research Council (BBSRC), UK (BB/M011186/1, 1945098).

AUTHOR CONTRIBUTIONS

Conceptualization: E.J.J. and E.G.C.-L.; data curation: E.J.J.; formal analysis: E.J.J., S.X., J.V.d.S, and A.E.; funding acquisition: E.G.C.-L.; investigation: E.J.J., S. X., J.V.d.S, and A.E.; project administration: E.G.C.-L.; supervision: A.K.B. and E.G.C.-L.; visualization: E.J.J., S.X., and A.E.; writing – original draft: E.J.J. and E.G.C.-L.; writing – review & editing: E.J.J., S.X., J.V.d.S, A.E., A.K.B., and E.G.C.-L.

DECLARATION OF INTERESTS

The authors declare no competing interests.

SUPPORTING MATERIAL

Supporting Material can be found online at https://doi.org/10.1016/j.bpj.2026.01.033.

REFERENCES

1. Shoulders, M. D., and R. T. Raines. 2009. Collagen structure and stability. *Annu. Rev. Biochem.* 78:929–958.
2. Fidler, A. L., S. P. Boudko, …, B. G. Hudson. 2018. The triple helix of collagens - an ancient protein structure that enabled animal multicellularity and tissue evolution. *J. Cell Sci.* 131:jcs203950.
3. Hofmann, H., P. P. Fietzek, and K. Kühn. 1978. The role of polar and hydrophobic interactions for the molecular packing of type I collagen: a three-dimensional evaluation of the amino acid sequence. *J. Mol. Biol.* 125:137–165.
4. Khoshnoodi, J., J. P. Cartailler, …, B. G. Hudson. 2006. Molecular recognition in the assembly of collagens: terminal noncollagenous domains are key recognition modules in the formation of triple helical protomers. *J. Biol. Chem.* 281:38117–38121.
5. Uitto, J. 1979. Collagen polymorphism: isolation and partial characterization of alpha 1(I)-trimer molecules in normal human skin. *Arch. Biochem. Biophys.* 192:371–379.
6. Jimenez, S. A., R. I. Bashey, …, R. Yankowski. 1977. Identification of collagen alpha1(I) trimer in embryonic chick tendons and calvaria. *Biochem. Biophys. Res. Commun.* 78:1354–1361.
7. Sharma, U., L. Carrique, …, D. J. S. Hulmes. 2017. Structural basis of homo- and heterotrimerization of collagen I. *Nat. Commun.* 8:14671.
8. Makareeva, E., S. Han, …, S. Leikin. 2010. Carcinomas Contain a Matrix Metalloproteinase–Resistant Isoform of Type I Collagen Exerting Selective Support to Invasion. *Cancer Res.* 70:4366–4374.
9. Chang, S. W., S. J. Shefelbine, and M. J. Buehler. 2012. Structural and mechanical differences between collagen homo- and heterotrimers: relevance for the molecular origin of brittle bone disease. *Biophys. J.* 102:640–648.

10. Miles, C. A., T. J. Sims, …, A. J. Bailey. 2002. The role of the alpha2 chain in the stabilization of the collagen type I heterotrimer: a study of the type I homotrimer in oim mouse tissues. *J. Mol. Biol.* 321:797–805.

11. Carriero, A., E. A. Zimmermann, …, S. J. Shefelbine. 2014. How tough is brittle bone? Investigating osteogenesis imperfecta in mouse bone. *J. Bone Miner. Res.* 29:1392–1401.

12. Sims, T. J., C. A. Miles, …, N. P. Camacho. 2003. Properties of collagen in OIM mouse tissues. *Connect. Tissue Res.* 44:202–205.

13. Pfeiffer, B. J., C. L. Franklin, …, C. L. Phillips. 2005. Alpha 2(I) collagen deficient oim mice have altered biomechanical integrity, collagen content, and collagen crosslinking of their thoracic aorta. *Matrix Biol.* 24:451–458.

14. Lee, K. J., L. Rambault, …, E. G. Canty-Laird. 2022. Collagen (I) homotrimer potentiates the osteogenesis imperfecta (oim) mutant allele and reduces survival in male mice. *Dis. Model. Mech.* 15:dmm049428.

15. Han, S., E. Makareeva, …, S. Leikin. 2010. Molecular mechanism of type I collagen homotrimer resistance to mammalian collagenases. *J. Biol. Chem.* 285:22276–22281.

16. Adhikari, A. S., E. Glassey, and A. R. Dunn. 2012. Conformational dynamics accompanying the proteolytic degradation of trimeric collagen I by collagenases. *J. Am. Chem. Soc.* 134:13259–13265.

17. Chang, S. W., B. P. Flynn, …, M. J. Buehler. 2012. Molecular mechanism of force induced stabilization of collagen against enzymatic breakdown. *Biomaterials.* 33:3852–3859.

18. Bourhis, J. M., N. Mariano, …, D. J. S. Hulmes. 2012. Structural basis of fibrillar collagen trimerization and related genetic disorders. *Nat. Struct. Mol. Biol.* 19:1031–1036.

19. Lees, J. F., M. Tasab, and N. J. Bulleid. 1997. Identification of the molecular recognition sequence which determines the type-specific assembly of procollagen. *EMBO J.* 16:908–916.

20. DiChiara, A. S., R. C. Li, …, M. D. Shoulders. 2018. A cysteine-based molecular code informs collagen C-propeptide assembly. *Nat. Commun.* 9:4206.

21. Yammine, K. M., R. C. Li, …, M. D. Shoulders. 2024. An outcome-defining role for the triple-helical domain in regulating collagen-I assembly. *Proc. Natl. Acad. Sci. USA.* 121:e2412948121.

22. Chessler, S. D., G. A. Wallis, and P. H. Byers. 1993. Mutations in the carboxyl-terminal propeptide of the pro alpha 1(I) chain of type I collagen result in defective chain association and produce lethal osteogenesis imperfecta. *J. Biol. Chem.* 268:18218–18225.

23. Waterhouse, A., M. Bertoni, …, T. Schwede. 2018. SWISS-MODEL: homology modelling of protein structures and complexes. *Nucleic Acids Res.* 46:W296–W303.

24. Abraham, M. J., T. Murtola, …, E. Lindahl. 2015. GROMACS: High performance molecular simulations through multi-level parallelism from laptops to supercomputers. *SoftwareX.* 1–2:19–25.

25. Páll, S., M. J. Abraham, …, E. Lindahl. 2015. Tackling Exascale Software Challenges in Molecular Dynamics Simulations with GROMACS. *In* Solving Software Challenges for Exascale. S. Markidis and E. Laure, eds Springer International Publishing, pp. 3–27.

26. Lindorff-Larsen, K., S. Piana, …, D. E. Shaw. 2010. Improved side-chain torsion potentials for the Amber ff99SB protein force field. *Proteins.* 78:1950–1958.

27. Hess, B., C. Kutzner, …, E. Lindahl. 2008. GROMACS 4: Algorithms for Highly Efficient, Load-Balanced, and Scalable Molecular Simulation. *J. Chem. Theory Comput.* 4:435–447.

28. Darden, T., D. York, and L. Pedersen. 1993. Particle mesh Ewald: An N·log(N) method for Ewald sums in large systems. *J. Chem. Phys.* 98:10089–10092.

29. Essmann, U., L. Perera, …, L. G. Pedersen. 1995. A smooth particle mesh Ewald method. *J. Chem. Phys.* 103:8577–8593.

30. Verlet, L. 1967. Computer “Experiments” on Classical Fluids. I. Thermodynamical Properties of Lennard-Jones Molecules. *Phys. Rev.* 159:98–103.

31. Hess, B., H. Bekker, …, J. G. E. M. Fraaije. 1997. LINCS: A linear constraint solver for molecular simulations. *J. Comput. Chem.* 18:1463–1472.

32. Humphrey, W., A. Dalke, and K. Schulten. 1996. VMD: visual molecular dynamics. *J. Mol. Graph.* 14:33.

33. Bürkner, P.-C. 2017. brms: An R Package for Bayesian Multilevel Models Using Stan. *J. Stat. Software.* 80:1–28.

34. Stan Development Team. 2025. Stan Reference Manual (v2.37.0). https://mc-stan.org.

35. Arel-Bundock, V., N. Greifer, and A. Heiss. 2024. How to Interpret Statistical Models Using marginaleffects for R and Python. *J. Stat. Softw.* 111:1–32.

36. Kay, M. 2024. tidybayes: Tidy Data and Geoms for Bayesian Models (v3.0.7). https://doi.org/10.5281/zenodo.13770114.

37. Wickham, H. 2016. ggplot2: Elegant Graphics for Data Analysis. Springer-Verlag.

38. Kokh, D. B., M. Amaral, …, R. C. Wade. 2018. Estimation of Drug-Target Residence Times by τ-Random Acceleration Molecular Dynamics Simulations. *J. Chem. Theory Comput.* 14:3859–3869.

39. Lemkul, J. A., and D. R. Bevan. 2010. Assessing the Stability of Alzheimer’s Amyloid Protofibrils Using Molecular Dynamics. *J. Phys. Chem. B.* 114:1652–1660.

40. Kumar, S., J. M. Rosenberg, …, P. A. Kollman. 1992. THE weighted histogram analysis method for free-energy calculations on biomolecules. I. The method. *J. Comput. Chem.* 13:1011–1021.

41. Hub, J. S., B. L. de Groot, and D. van der Spoel. 2010. g_wham—A Free Weighted Histogram Analysis Implementation Including Robust Error and Autocorrelation Estimates. *J. Chem. Theory Comput.* 6:3713–3720.

42. Shirts, M. R., and D. L. Mobley. 2013. An Introduction to Best Practices in Free Energy Calculations. *In* Biomolecular Simulations: Methods and Protocols. L. Monticelli and E. Salonen, eds Humana Press, pp. 271–311.

43. Gapsys, V., S. Michielssens, …, B. L. de Groot. 2015. pmx: Automated protein structure and topology generation for alchemical perturbations. *J. Comput. Chem.* 36:348–354.

44. Seeliger, D., and B. L. de Groot. 2010. Protein thermostability calculations using alchemical free energy simulations. *Biophys. J.* 98:2309–2316.

45. Abramson, J., J. Adler, …, J. M. Jumper. 2024. Accurate structure prediction of biomolecular interactions with AlphaFold 3. *Nature.* 630:493–500.

46. Galgonek, J., J. Vymetal, …, J. Vondrášek. 2017. Amino Acid Interaction (INTAA) web server. *Nucleic Acids Res.* 45:W388–W392.

47. Gao, X., X. Dong, …, H. Liu. 2020. Prediction of disulfide bond engineering sites using a machine learning method. *Sci. Rep.* 10:10330.

48. Sun, M. A., Y. Wang, …, D. Guo. 2017. Prediction of reversible disulfide based on features from local structural signatures. *BMC Genom.* 18:279.

49. Mowrey, D. D., L. Xu, …, N. V. Dokholyan. 2017. Ion-pulling simulations provide insights into the mechanisms of channel opening of the skeletal muscle ryanodine receptor. *J. Biol. Chem.* 292:12947–12958.

50. Limongelli, V. 2020. Ligand binding free energy and kinetics calculation in 2020. *WIREs Comput. Mol. Sci.* 10:e1455.

51. Kastritis, P. L., and A. M. J. J. Bonvin. 2013. On the binding affinity of macromolecular interactions: daring to ask why proteins interact. *J. R. Soc. Interface.* 10:20120835.

52. Schreiber, G., and A. R. Fersht. 1995. Energetics of protein-protein interactions: analysis of the barnase-barstar interface by single mutations and double mutant cycles. *J. Mol. Biol.* 248:478–486.

53. Malfait, F., S. Symoens, …, A. De Paepe. 2006. Total absence of the alpha2(I) chain of collagen type I causes a rare form of Ehlers-Danlos syndrome with hypermobility and propensity to cardiac valvular problems. *J. Med. Genet.* 43:e36.

54. Chen, Y., S. Yang, …, R. Kalluri. 2022. Oncogenic collagen I homotrimers from cancer cells bind to α3β1 integrin and impact tumor microbiome and immunity to promote pancreatic cancer. *Cancer Cell.* 40:818–834.e9.

55. Myllyharju, J., A. Lamberg, …, K. I. Kivirikko. 1997. Expression of wild-type and modified proalpha chains of human type I procollagen in insect cells leads to the formation of stable [alpha1(I)]2alpha2(I) collagen heterotrimers and [alpha1(I)]3 homotrimers but not [alpha2(I)]3 homotrimers. *J. Biol. Chem.* 272:21824–21830.

56. Mann, V., E. E. Hobson, …, S. H. Ralston. 2001. A COL1A1 Sp1 binding site polymorphism predisposes to osteoporotic fracture by affecting bone density and quality. *J. Clin. Investig.* 107:899–907.

57. Barsh, G. S., K. E. David, and P. H. Byers. 1982. Type I osteogenesis imperfecta: a nonfunctional allele for pro alpha 1 (I) chains of type I procollagen. *Proc. Natl. Acad. Sci. USA.* 79:3838–3842.

58. Williamson, K., K. J. Lee, …, E. G. Canty-Laird. 2025. Active synthesis of type I collagen homotrimer in Dupuytren's fibrosis is unaffected by anti-TNF-α treatment. *JCI Insight.* 10:e175188.

59. Stefanovic, B., L. Stefanovic, and Z. Manojlovic. 2021. Imaging of type I procollagen biosynthesis in cells reveals biogenesis in highly organized bodies. *Matrix Biol.* 12:100076.

60. Putney, J. W., Jr. 1986. A model for receptor-regulated calcium entry. *Cell Calcium.* 7:1–12.

61. Mekahli, D., G. Bultynck, …, L. Missiaen. 2011. Endoplasmic-reticulum calcium depletion and disease. *Cold Spring Harb. Perspect. Biol.* 3:a004317.

62. Cabral, W. A., M. Ishikawa, …, J. C. Marini. 2016. Absence of the ER Cation Channel TMEM38B/TRIC-B Disrupts Intracellular Calcium Homeostasis and Dysregulates Collagen Synthesis in Recessive Osteogenesis Imperfecta. *PLoS Genet.* 12:e1006156.

63. Viner, R. I., T. D. Williams, and C. Schöneich. 1999. Peroxynitrite modification of protein thiols: oxidation, nitrosylation, and S-glutathiolation of functionally important cysteine residue(s) in the sarcoplasmic reticulum Ca-ATPase. *Biochemistry.* 38:12408–12415.

64. Puzianowska-Kuznicka, M., and J. Kuznicki. 2009. The ER and ageing II: calcium homeostasis. *Ageing Res. Rev.* 8:160–172.

65. Celli, A., C. L. Tu, …, T. M. Mauro. 2021. Decreased Calcium-Sensing Receptor Expression Controls Calcium Signaling and Cell-To-Cell Adhesion Defects in Aged Skin. *J. Invest. Dermatol.* 141:2577–2586.

66. Ruder, W. C., E. D. Pratt, …, J. F. Antaki. 2012. Calcium signaling is gated by a mechanical threshold in three-dimensional environments. *Sci. Rep.* 2:554.

67. Donati, V., C. Peres, …, F. Mammano. 2022. Calcium Signaling in the Photodamaged Skin: In Vivo Experiments and Mathematical Modeling. *Function (Oxf).* 3:zqab064.

68. Foggia, L., and A. Hovnanian. 2004. Calcium pump disorders of the skin. *Am. J. Med. Genet. C Semin. Med. Genet.* 131C:20–31.

69. Saab, J., I. M. Rosenthal, …, T. J. Hollmann. 2017. Dermatofibrosarcoma Protuberans-Like Tumor With COL1A1 Copy Number Gain in the Absence of t(17;22). *Am. J. Dermatopathol.* 39:304–309.

70. Monteith, G. R., D. McAndrew, …, S. J. Roberts-Thomson. 2007. Calcium and cancer: targeting Ca2+ transport. *Nat. Rev. Cancer.* 7:519–530.

71. Symoens, S., D. J. S. Hulmes, …, F. Malfait. 2014. Type I procollagen C-propeptide defects: study of genotype-phenotype correlation and predictive role of crystal structure. *Hum. Mutat.* 35:1330–1341.

72. Doan, N. D., A. S. Hosseini, …, M. D. Shoulders. 2020. Elucidation of proteostasis defects caused by osteogenesis imperfecta mutations in the collagen-alpha2(I) C-propeptide domain. *J. Biol. Chem.* 295:9959–9973.

73. Nüsse, O., L. Serrander, …, K. H. Krause. 1997. Store-operated Ca2+ influx and stimulation of exocytosis in HL-60 granulocytes. *J. Biol. Chem.* 272:28360–28367.

74. Werno, C., J. Zhou, and B. Brüne. 2008. A23187, ionomycin and thapsigargin upregulate mRNA of HIF-1alpha via endoplasmic reticulum stress rather than a rise in intracellular calcium. *J. Cell. Physiol.* 215:708–714.

75. Huang, Y., and J. W. Putney. 1998. Relationship between Intracellular Calcium Store Depletion and Calcium Release-activated Calcium Current in a Mast Cell Line (RBL-1). *J. Biol. Chem.* 273:19554–19559.

76. Tse, C., L. Wickstrom, …, N. Deng. 2020. Exploring the Free-Energy Landscape and Thermodynamics of Protein-Protein Association. *Biophys. J.* 119:1226–1238.

77. Domański, J., G. Hedger, …, M. S. P. Sansom. 2017. Convergence and Sampling in Determining Free Energy Landscapes for Membrane Protein Association. *J. Phys. Chem. B.* 121:3364–3375.

Biophysical Journal, Volume *125*

Supplemental information

Molecular dynamics reveals how calcium drives hetero- versus homo-dimerization of type I collagen

Emily J. Johnson, Shangze Xu, João V. de Souza, Anthony Evans, Agnieszka K. Bronowska, and Elizabeth G. Canty-Laird

Supporting Material for "Molecular dynamics reveals how calcium drives hetero- versus homo-trimerisation of type I collagen"

Emily J Johnson[1,2,3], Shangze Xu[4], João V de Souza[4,5], Anthony Evans[2,3], Agnieszka K Bronowska[4,6] and Elizabeth G Canty-Laird[1]

[1]Department of Musculoskeletal and Ageing Science, Institute of Life Course and Medical Sciences, University of Liverpool, William Henry Duncan Building, 6 West Derby Street, Liverpool, L7 8TX, United Kingdom

[2]Computational Biology Facility, LIV-SRF, MerseyBio, University of Liverpool, Crown Street, Liverpool, L69 7ZB, United Kingdom

[3] Institute of Systems, Molecular and Integrative Biology, University of Liverpool, Liverpool L69 7ZB, United Kingdom

[4] Chemistry-School of Natural and Environmental Sciences, Newcastle University, Newcastle Upon Tyne, NE1 7RU, United Kingdom

[5] Current location: RxCelerate Ltd, Babraham Research Campus, Cambridge CB22 3FH, United Kingdom

[6] Newcastle University Centre for Cancer, Newcastle University, Newcastle Upon Tyne, NE1 7RU, United Kingdom

Supplementary Tables

Table S1: Time averaged structural properties calculated for the homotrimer, heterotrimer, apo-homotrimer and apo-heterotrimer across three replicates.

Trimer type	Backbone RMSD (nm)	Backbone-Rg (nm)
Holo-homotrimer	0.564 (0.015)	2.867 (0.007)
Holo-heterotrimer	0.511 (0.037)	2.878 (0.035)
Apo-homotrimer	0.632 (0.120)	2.877 (0.044)
Apo-heterotrimer	0.540 (0.032)	2.895 (0.021)

Each value represents the mean across three replicate trajectories, with standard errors shown in parentheses. Standard errors were estimated using block averaging with a block size of 50 ns, determined from analysis of the standard error dependence on block size (see Figure S5).

Table S2. Notable hydrogen bonds in the homotrimer and heterotrimer.

Trimer	Bond	Position in full length chain	Inter-chain/ intra-chain	Weight
Homotrimer	ARG 42.A - ASP 129.B	ARG 1260.A - ASP 1347.B	Inter-chain	2.00794
Homotrimer	ARG 39.B - ASN 61.C	ARG 1257.B - ASN 1279.C	Inter-chain	1.48413
Homotrimer	ARG 42.B - ASP 129.C	ARG 1260.B - ASP 1347.C	Inter-chain	1.1746
Homotrimer	ASP 43.A - CYS 64.B	ASP 1261.A - CYS 1282.B	Inter-chain	1.09524
Homotrimer	ALA 128.C - ARG 42.B	ALA 1346.C - ARG 1260.B	Inter-chain	0.650794
Homotrimer	ARG 42.C - ASP 129.A	ARG 1260.C - ASP 1347.A	Inter-chain	0.579365
Homotrimer	ALA 128.A - ARG 42.C	ALA 1346.A - ARG 1260.C	Inter-chain	0.515873
Homotrimer	ARG 42.A - ASP 67.B	ARG 1260.A - ASP 1285.B	Inter-chain	0.460317
Homotrimer	ASP 43.C - CYS 64.A	ASP 1261.C - CYS 1282.A	Inter-chain	0.444444
Homotrimer	ARG 39.C - ASN 61.A	ARG 1257.C - ASN 1279.A	Inter-chain	0.380952

Homotrimer	ARG 39.A - ASN 61.B	ARG 1257.A - ASN 1279.B	Inter-chain	0.301587
Homotrimer	ARG 39.B - GLN 62.C	ARG 1257.B - GLN 1280.C	Inter-chain	0.301587
Homotrimer	CYS 64.C - MET 46.B	CYS 1282.C - MET 1264.B	Inter-chain	0.246032
Homotrimer	PHE 245.A - THR 80.A	PHE 1463.A - THR 1298.A	Intra-chain	2.01587
Homotrimer	ILE 58.A - ILE 69.A	ILE 1276.A - ILE 1287.A	Intra-chain	2
Homotrimer	TYR 56.C - VAL 71.C	TYR 1274.C - VAL 1289.C	Intra-chain	2
Homotrimer	ILE 58.B - ILE 69.B	ILE 1276.B - ILE 1287.B	Intra-chain	2
Homotrimer	ILE 58.C - ILE 69.C	ILE 1276.B - ILE 1287.B	Intra-chain	2
Homotrimer	TYR 56.A - VAL 71.A	TYR 1274.A - VAL 1289.A	Intra-chain	2
Homotrimer	TYR 56.B - VAL 71.B	TYR 1274.B - VAL 1289.B	Intra-chain	1.98413
Homotrimer	CYS 41.B - THR 80.B	CYS 1259.B - THR 1298.B	Intra-chain	1.7619
Homotrimer	CYS 41.A - THR 80.A	CYS 1259.A - THR 1298.A	Intra-chain	1.68254
Homotrimer	CYS 41.C - THR 80.C	CYS 1259.C - THR 1298.C	Intra-chain	1.61905
Homotrimer	ARG 39.B - ASP 43.B	ARG 1257.B - ASP 1261.B	Intra-chain	1.53968
Homotrimer	ASP 43.B - CYS 47.B	ASP 1261.B - CYS 1265.B	Intra-chain	1.38889
Homotrimer	ASN 61.B - GLN 133.B	ASN 1279.B - GLN 1351.B	Intra-chain	1.24603
Homotrimer	ASP 43.C - THR 40.C	ASP 1261.C - THR 1258.C	Intra-chain	1.23016
Homotrimer	ASP 43.B - THR 40.B	ASP 1261.B - THR 1258.B	Intra-chain	1.14286
Homotrimer	ASN 61.A - GLN 133.A	ASN 1279.A - GLN 1351.A	Intra-chain	1.05556
Homotrimer	ASN 61.C - GLN 133.C	ASN 1279.C - GLN 1351.C	Intra-chain	1.03968
Homotrimer	ASP 43.C - CYS 47.C	ASP 1261.C - CYS 1265.C	Intra-chain	0.968254
Homotrimer	ASP 43.A - THR 40.A	ASP 1261.A - THR 1258.A	Intra-chain	0.912698
Homotrimer	ASN 61.B - ASP 67.B	ASN 1279.B - ASP 1285.B	Intra-chain	0.888889
Homotrimer	ARG 42.B - LEU 246.B	ARG 1260.B -LEU 1464.B	Intra-chain	0.873016
Homotrimer	ARG 39.C - PRO 60.C	ARG 1257.C - PRO 1278.C	Intra-chain	0.865079
Homotrimer	ASN 61.A - ILE 132.A	ASN 1279.A - ILE 1350.A	Intra-chain	0.857143
Homotrimer	ASN 61.C - ASP 67.C	ASN 1279.C - ASP 1255.C	Intra-chain	0.84127
Homotrimer	ASN 61.A - ASP 67.A	ASN 1279.A - ASP 1255.A	Intra-chain	0.84127
Homotrimer	ARG 42.C - MET 46.C	ARG 1260.C - MET 1264.C	Intra-chain	0.785714
Homotrimer	ARG 42.B - THR 142.B	ARG 1260.B - THR 1360.B	Intra-chain	0.769841
Homotrimer	ASP 43.A - CYS 47.A	ASP 1261.A - CYS 1265.A	Intra-chain	0.746032
Homotrimer	ARG 42.A - MET 46.A	ARG 1260.A - MET 1264.A	Intra-chain	0.68254
Homotrimer	ARG 42.C - LEU 246.C	ARG 1260.C - LEU 1464.C	Intra-chain	0.674603
Homotrimer	ARG 39.C - ASP 43.C	ARG 1257.C - ASP 1261.C	Intra-chain	0.650794
Homotrimer	ARG 39.A - ASP 43.A	ARG 1257.A - ASP 1261.A	Intra-chain	0.619048
Homotrimer	ARG 42.B - MET 46.B	ARG 1260.B - MET 1264.B	Intra-chain	0.595238
Homotrimer	ARG 42.C - THR 142.C	ARG 1260.C - THR 1360.C	Intra-chain	0.5
Homotrimer	ARG 39.B - GLN 62.B	ARG 1257.B - GLN 1280.B	Intra-chain	0.468254
Homotrimer	ASN 61.C - ASP 59.C	ASN 1279.C - ASP 1277.C	Intra-chain	0.468254
Homotrimer	ARG 39.B - PRO 60.B	ARG 1257.B - PRO 1278.B	Intra-chain	0.34127
Homotrimer	ASN 61.B - ILE 132.B	ASN 1279.B - ILE 1350.B	Intra-chain	0.293651
Heterotrimer	ARG 42.A - GLU 130.B	ARG 1260.A - GLU 1249.B	Inter-chain	2.45238
Heterotrimer	ARG 39.A - ASN 64.B	ARG 1257.A - ASN 1183.B	Inter-chain	1.59524
Heterotrimer	ASP 43.A - CYS 67.B	ASP 1261.A - CYS 1186.B	Inter-chain	1.26984
Heterotrimer	ARG 42.C - ASP 129.A	ARG 1260.C - ASP 1347.A	Inter-chain	0.984127
Heterotrimer	ARG 39.C - ASN 61.A	ARG 1257.C - ASN 1279.A	Inter-chain	0.984127
Heterotrimer	ARG 42.B - ASN 61.C	ARG 1161.B - ASN 1279.C	Inter-chain	0.896825
Heterotrimer	ASP 46.B - CYS 64.C	ASP 1165.B - CYS 1282.C	Inter-chain	0.746032
Heterotrimer	ARG 45.B - ASP 129.C	ARG 1164.B - ASP 1347.C	Inter-chain	0.587302
Heterotrimer	ARG 45.B - ASP 67.C	ARG 1164.B - ASP 1285.C	Inter-chain	0.103175
Heterotrimer	ASP 43.C - CYS 64.A	ASP 1261.C - CYS 1282.A	Inter-chain	0.103175

Heterotrimer	ASP 43.A - THR 40.A	ASP 1261.A - THR 1258.A	Intra-chain	2.15873
Heterotrimer	ARG 42.A - LEU 246.A	ARG 1260.A -LEU 1464.A	Intra-chain	2.01587
Heterotrimer	TYR 56.C - VAL 71.C	TYR 1274.C - VAL 1289.C	Intra-chain	2
Heterotrimer	TYR 56.A - VAL 71.A	TYR 1274.A - VAL 1289.A	Intra-chain	2
Heterotrimer	ILE 58.A - ILE 69.A	ILE 1276.A - ILE 1287.A	Intra-chain	2
Heterotrimer	TYR 59.B - VAL 74.B	TYR 1178.B - VAL 1193.B	Intra-chain	1.99206
Heterotrimer	ILE 61.B - ILE 72.B	ILE 1180.B - ILE 1191.B	Intra-chain	1.99206
Heterotrimer	ILE 58.C - ILE 69.C	ILE 1276.C - ILE 1287.C	Intra-chain	1.99206
Heterotrimer	ASP 46.B - THR 43.B	ASP 1165.B - THR 1162.B	Intra-chain	1.96032
Heterotrimer	ARG 39.C - PRO 60.C	ARG 1257.C - PRO 1278.C	Intra-chain	1.94444
Heterotrimer	ARG 42.B - PRO 63.B	ARG 1161.B - PRO 1182.B	Intra-chain	1.88889
Heterotrimer	CYS 41.C - THR 80.C	CYS 1259.C - THR 1298.C	Intra-chain	1.69841
Heterotrimer	ASP 59.C - GLN 62.C	ASP 1277.C - GLN 1280.C	Intra-chain	1.57143
Heterotrimer	CYS 41.A - THR 80.A	CYS 1259.A - THR 1298.A	Intra-chain	1.53968
Heterotrimer	ASP 43.A - CYS 47.A	ASP 1261.A - CYS 1265.A	Intra-chain	1.5
Heterotrimer	ASP 59.A - GLN 62.A	ASP 1277.A - GLN 1280.A	Intra-chain	1.47619
Heterotrimer	CYS 44.B - THR 83.B	CYS 1163.B - THR 1202.B	Intra-chain	1.46032
Heterotrimer	ARG 45.B - PHE 246.B	ARG 1164.B - PHE 1365.B	Intra-chain	1.36508
Heterotrimer	ARG 39.A - ASP 43.A	ARG 1257.A - ASP 1261.A	Intra-chain	1.33333
Heterotrimer	ASP 43.C - CYS 47.C	ASP 1261.C - CYS 1265.C	Intra-chain	1.30159
Heterotrimer	ASP 43.C - THR 40.C	ASP 1261.C - THR 1258.C	Intra-chain	1.27778
Heterotrimer	ASN 61.A - GLN 133.A	ASN 1279.A - GLN 1351.A	Intra-chain	1.24603
Heterotrimer	ASN 61.C - GLN 133.C	ASN 1279.C - GLN 1351.C	Intra-chain	1.03968
Heterotrimer	ASN 64.B - GLN 134.B	ASN 1183.B - GLN 1253.B	Intra-chain	1.03175
Heterotrimer	ASP 70.B - GLN 134.B	ASP 1189.B - GLN 1253.B	Intra-chain	1
Heterotrimer	ASP 67.A - GLN 133.A	ASP 1285.A - GLN 1351.A	Intra-chain	0.992063
Heterotrimer	ARG 42.C - LEU 246.C	ARG 1260.C - LEU 1464.C	Intra-chain	0.984127
Heterotrimer	ASN 61.A - ASP 67.A	ASN 1279.A - ASP 1285.A	Intra-chain	0.960317
Heterotrimer	ASN 61.C - ILE 132.C	ASN 1279.C - ILE 1350.C	Intra-chain	0.928571
Heterotrimer	ARG 45.B - LEU 49.B	ARG 1164.B - LEU 1168.B	Intra-chain	0.920635
Heterotrimer	ASN 64.B - ASP 70.B	ASN 1183.B - ASP 1189.B	Intra-chain	0.84127
Heterotrimer	ARG 39.C - ASP 43.C	ARG 1257.C - ASP 1261.C	Intra-chain	0.801587
Heterotrimer	ASN 64.B - THR 133.B	ASN 1183.B - THR 1252.B	Intra-chain	0.690476
Heterotrimer	ARG 42.C - MET 46.C	ARG 1260.C - MET 1264.C	Intra-chain	0.674603
Heterotrimer	ARG 42.A - MET 46.A	ARG 1260.A - MET 1264.A	Intra-chain	0.65873
Heterotrimer	ASN 61.A - ILE 132.A	ASN 1279.A - ILE 1350.A	Intra-chain	0.515873
Heterotrimer	ARG 42.B - ASP 46.B	ARG 1161.B - ASP 1165.B	Intra-chain	0.253968
Heterotrimer	ASP 67.C - GLN 133.C	ASP 1285.C - GLN 1351.C	Intra-chain	0.222222
Heterotrimer	ASN 61.C - ASP 67.C	ASN 1279.C - ASP 1285.C	Intra-chain	0.142857

Each trimer has three chains: A, B and C. In the homotrimer all three chains are α1. In the heterotrimer chains A and C are α1(I) and chain B is α2(I)α1(1) is denoted chain A, α2 in the heterotrimer and α1(2) in the homotrimer are denoted chain B, whilst α1(3) is denoted chain C. The weight corresponds to how conserved the bond was throughout the simulation, with higher weights corresponding to the most conserved bonds and lower weights corresponding to more transient bonds.

Table S3. ΔRMSF values between the apo- and holo- forms of the homotrimer and heterotrimer.

Trimer	Residue	Chain	Difference	Mean Holo	Mean Apo
Heterotrimer	26	A	0.1279	0.159567	0.287467
Heterotrimer	125	A	0.126733	0.173633	0.300367
Heterotrimer	25	A	0.116633	0.177433	0.294067
Heterotrimer	27	A	0.107333	0.151067	0.2584
Heterotrimer	126	A	0.101033	0.1857	0.286733
Heterotrimer	124	A	0.099167	0.190267	0.289433
Heterotrimer	129	A	0.082233	0.150167	0.2324
Heterotrimer	65	A	0.075333	0.1395	0.214833
Heterotrimer	187	A	0.066433	0.2024	0.268833
Heterotrimer	28	A	0.066	0.220633	0.286633
Heterotrimer	66	A	0.064833	0.137567	0.2024
Heterotrimer	64	A	0.061467	0.123267	0.184733
Heterotrimer	128	A	0.0591	0.156133	0.215233
Heterotrimer	103	A	0.051433	0.2087	0.260133
Heterotrimer	67	A	0.0501	0.107133	0.157233
Heterotrimer	102	B	0.1425	0.416633	0.559133
Heterotrimer	26	B	0.110533	0.190567	0.3011
Heterotrimer	25	B	0.101267	0.190033	0.2913
Heterotrimer	99	B	0.0898	0.306333	0.396133
Heterotrimer	101	B	0.0743	0.3144	0.3887
Heterotrimer	106	B	0.0651	0.1534	0.2185
Heterotrimer	104	B	0.059367	0.326	0.385367
Heterotrimer	68	B	0.059233	0.117267	0.1765
Heterotrimer	100	B	0.054067	0.250067	0.304133
Heterotrimer	75	B	-0.05	0.176933	0.126933
Heterotrimer	176	B	-0.05	0.1353	0.0853
Heterotrimer	58	B	-0.05013	0.188233	0.1381
Heterotrimer	56	B	-0.05347	0.1745	0.121033
Heterotrimer	82	B	-0.05433	0.174867	0.120533
Heterotrimer	178	B	-0.0555	0.184533	0.129033
Heterotrimer	86	B	-0.0561	0.2923	0.2362
Heterotrimer	54	B	-0.0562	0.2041	0.1479
Heterotrimer	48	B	-0.05913	0.200667	0.141533
Heterotrimer	112	B	-0.0618	0.235533	0.173733
Heterotrimer	124	B	-0.06367	0.268867	0.2052
Heterotrimer	123	B	-0.0668	0.2286	0.1618
Heterotrimer	125	B	-0.06723	0.224633	0.1574
Heterotrimer	81	B	-0.06733	0.176967	0.109633
Heterotrimer	80	B	-0.07107	0.210833	0.139767
Heterotrimer	122	B	-0.07117	0.2454	0.174233
Heterotrimer	87	B	-0.07897	0.1867	0.107733
Heterotrimer	117	B	-0.07913	0.270867	0.191733
Heterotrimer	113	B	-0.07937	0.261633	0.182267

Heterotrimer	51	B	-0.08027	0.239367	0.1591
Heterotrimer	116	B	-0.09653	0.280267	0.183733
Heterotrimer	114	B	-0.10797	0.357733	0.249767
Heterotrimer	115	B	-0.12227	0.350533	0.228267
Heterotrimer	28	C	0.106433	0.2417	0.348133
Heterotrimer	67	C	0.0889	0.1387	0.2276
Heterotrimer	125	C	0.0863	0.168733	0.255033
Heterotrimer	123	C	0.081333	0.269333	0.350667
Heterotrimer	124	C	0.077867	0.205033	0.2829
Heterotrimer	127	C	0.075467	0.135067	0.210533
Heterotrimer	64	C	0.069533	0.126767	0.1963
Heterotrimer	198	C	0.069433	0.1126	0.182033
Heterotrimer	65	C	0.067167	0.1599	0.227067
Heterotrimer	161	C	0.063267	0.1866	0.249867
Heterotrimer	126	C	0.057367	0.181267	0.238633
Heterotrimer	27	C	0.056867	0.171467	0.228333
Heterotrimer	162	C	0.056533	0.210433	0.266967
Heterotrimer	29	C	0.0538	0.127133	0.180933
Heterotrimer	98	C	-0.0515	0.403967	0.352467
Heterotrimer	104	C	-0.07797	0.375133	0.297167
Heterotrimer	103	C	-0.1076	0.457867	0.350267
Heterotrimer	102	C	-0.1169	0.4181	0.3012
Heterotrimer	99	C	-0.13567	0.417967	0.2823
Heterotrimer	100	C	-0.1395	0.465133	0.325633
Homotrimer	26	A	0.190767	0.182933	0.3737
Homotrimer	25	A	0.1724	0.179767	0.352167
Homotrimer	28	A	0.1613	0.225767	0.387067
Homotrimer	27	A	0.1572	0.167467	0.324667
Homotrimer	103	A	0.104833	0.1876	0.292433
Homotrimer	66	A	0.101533	0.151767	0.2533
Homotrimer	161	A	0.093567	0.196367	0.289933
Homotrimer	29	A	0.093433	0.131967	0.2254
Homotrimer	101	A	0.092467	0.3903	0.482767
Homotrimer	64	A	0.089133	0.138333	0.227467
Homotrimer	162	A	0.0819	0.220933	0.302833
Homotrimer	185	A	0.076267	0.165867	0.242133
Homotrimer	63	A	0.071433	0.1186	0.190033
Homotrimer	177	A	0.071033	0.153567	0.2246
Homotrimer	67	A	0.0696	0.116133	0.185733
Homotrimer	65	A	0.066233	0.1531	0.219333
Homotrimer	102	A	0.064767	0.2411	0.305867
Homotrimer	204	A	0.062433	0.0934	0.155833
Homotrimer	100	A	0.061233	0.2943	0.355533
Homotrimer	129	A	0.060233	0.147933	0.208167
Homotrimer	124	A	0.056167	0.2093	0.265467
Homotrimer	186	A	0.055833	0.1726	0.228433

Homotrimer	104	A	0.053667	0.236733	0.2904
Homotrimer	30	A	0.053567	0.115767	0.169333
Homotrimer	125	A	0.052467	0.188133	0.2406
Homotrimer	34	A	0.0523	0.1584	0.2107
Homotrimer	99	A	0.05	0.2543	0.3043
Homotrimer	28	B	0.124167	0.2363	0.360467
Homotrimer	27	B	0.1211	0.164667	0.285767
Homotrimer	25	B	0.107133	0.187767	0.2949
Homotrimer	26	B	0.094833	0.1858	0.280633
Homotrimer	87	B	0.091933	0.1013	0.193233
Homotrimer	65	B	0.073567	0.158233	0.2318
Homotrimer	64	B	0.0646	0.135233	0.199833
Homotrimer	66	B	0.0634	0.151833	0.215233
Homotrimer	163	B	0.061333	0.1714	0.232733
Homotrimer	125	B	0.0577	0.162333	0.220033
Homotrimer	164	B	0.056633	0.1501	0.206733
Homotrimer	46	B	0.056167	0.137767	0.193933
Homotrimer	124	B	0.056133	0.1806	0.236733
Homotrimer	67	B	0.052233	0.111233	0.163467
Homotrimer	104	B	-0.05113	0.301933	0.2508
Homotrimer	103	B	-0.16427	0.408333	0.244067
Homotrimer	28	C	0.158333	0.234133	0.392467
Homotrimer	25	C	0.146133	0.191	0.337133
Homotrimer	125	C	0.1292	0.195367	0.324567
Homotrimer	27	C	0.125	0.189933	0.314933
Homotrimer	26	C	0.1143	0.203833	0.318133
Homotrimer	66	C	0.106533	0.168733	0.275267
Homotrimer	126	C	0.104967	0.210033	0.315
Homotrimer	129	C	0.0945	0.177733	0.272233
Homotrimer	124	C	0.093333	0.217133	0.310467
Homotrimer	128	C	0.0933	0.188233	0.281533
Homotrimer	127	C	0.0884	0.190567	0.278967
Homotrimer	67	C	0.087133	0.130933	0.218067
Homotrimer	64	C	0.0862	0.158333	0.244533
Homotrimer	195	C	0.085	0.119433	0.204433
Homotrimer	123	C	0.072433	0.318767	0.3912
Homotrimer	65	C	0.0717	0.1742	0.2459
Homotrimer	103	C	0.071	0.341467	0.412467
Homotrimer	196	C	0.0612	0.100067	0.161267
Homotrimer	87	C	0.059733	0.135233	0.194967
Homotrimer	98	C	0.0553	0.342233	0.397533
Homotrimer	114	C	0.053733	0.132867	0.1866
Homotrimer	46	C	0.050167	0.144033	0.1942
Homotrimer	104	C	-0.05253	0.365333	0.3128

Results are sorted by trimer, then chain, then ΔRMSF value. Positive values are residues that were destabilised in response to calcium depletion and negative values were residues that were stablised in response to calcium depletion.

Table S4. Inter-trimer type differences in mean distance predictions.

Contrast	Difference in mean distance predictions (median, 95% CrI)
Chain interface A:C2 – B:C3	
Apo-homotrimer - Homotrimer	0.2 (0.19–0.21)
Apo-heterotrimer - Heterotrimer	0.25 (0.24–0.26)
Heterotrimer - Homotrimer	0.0044 (-0.0023–0.011)
Apo-heterotrimer - Apo-homotrimer	0.053 (0.046–0.06)
Chain interface B:C2 – C:C3	
Apo-homotrimer - Homotrimer	0.28 (0.28–0.29)
Apo-heterotrimer - Heterotrimer	0.1 (0.094–0.11)
Heterotrimer - Homotrimer	0.038 (0.032–0.044)
Apo-heterotrimer - Apo-homotrimer	-0.15 (-0.15–-0.14)
Chain interface C:C2 – A:C3	
Apo-homotrimer - Homotrimer	0.3 (0.3–0.31)
Apo-heterotrimer - Heterotrimer	0.21 (0.2–0.21)
Heterotrimer - Homotrimer	0.04 (0.033–0.047)
Apo-heterotrimer - Apo-homotrimer	-0.059 (-0.066–-0.052)

For each contrast, mean distance predictions were estimated for the stated trimer types using all draws from the model posterior. The differences in per-draw predictions between trimer type were calculated and summarised as the median plus 95% credible (quantile) intervals.

Table S5. Comparisons between umbrella sampling of calcium ion uncoupling from α1(I) or α2(I) chain

Comparison	α1	α2	Difference α1 − α2
ΔG_min (binding well)	−4.99	−3.52	−1.5 kcal mol^{-1}

Supplementary Figures

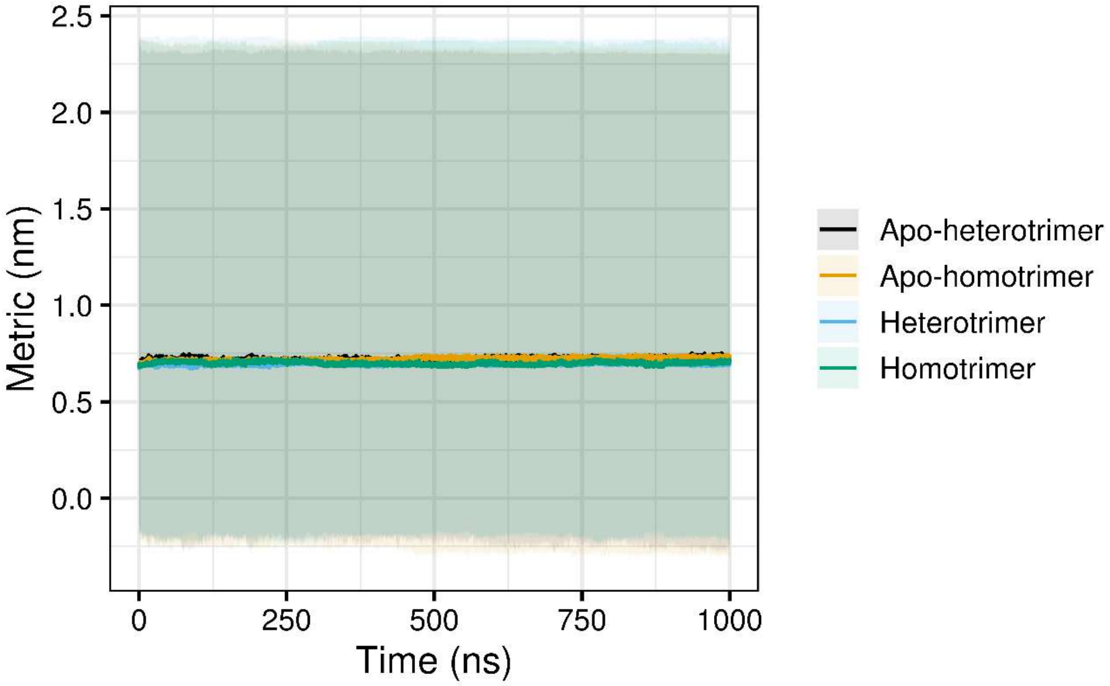

Figure S1: Prior predictive check for Bayesian linear autoregressive (AR) models. Implied model predictions from the weakly informative priors are shown, with no effect of time or trimer type imposed. Predictions for the metrics of interest (distance or RMSD) are mostly positive and fall within a plausible range of values. The means of the distributions are plotted as lines, with shaded regions indicating 95% credible intervals.

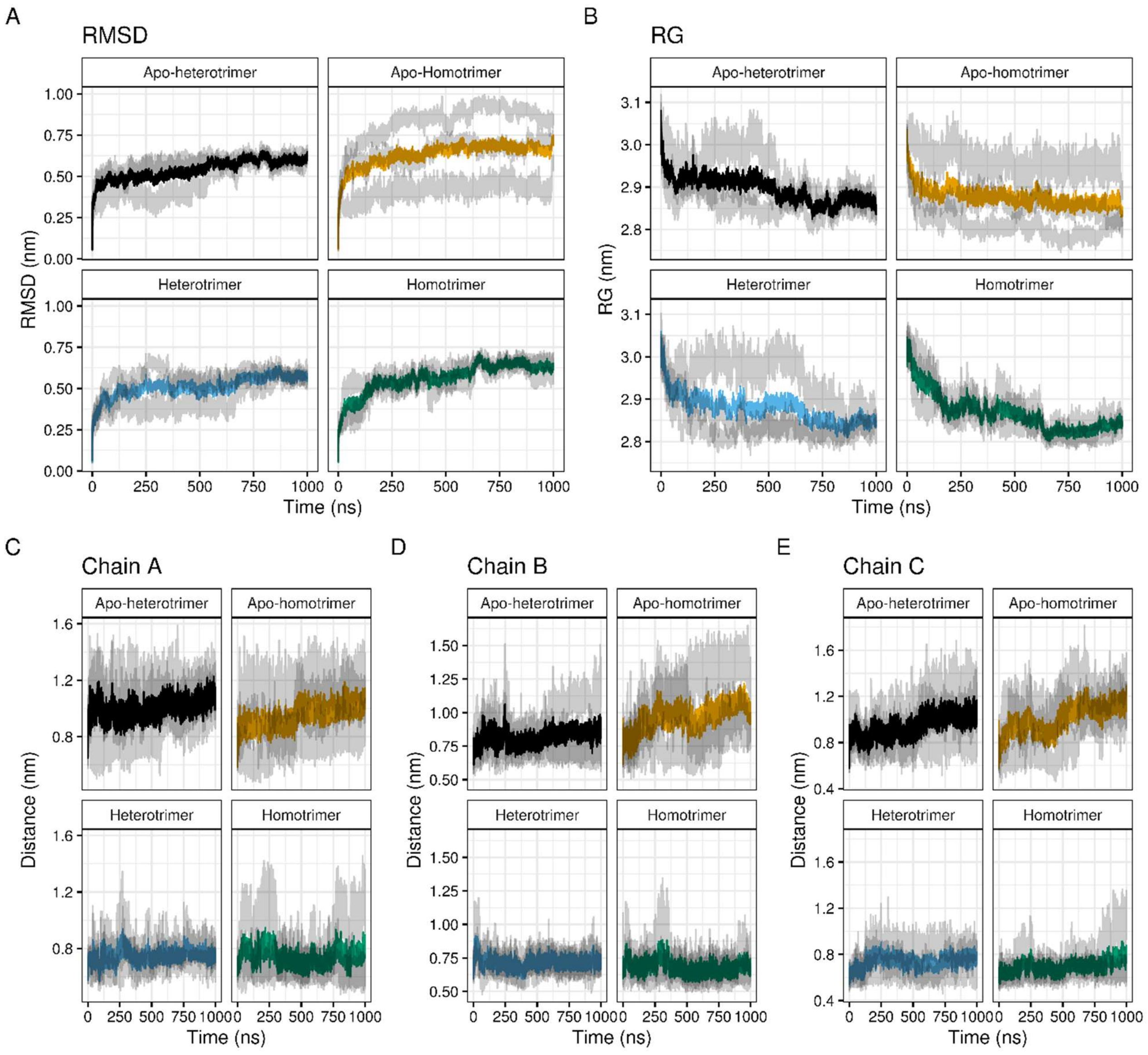

Figure S2: Posterior predictive checks on the fitted Bayesian linear autoregressive (AR) models. Posterior mean predicted distances are plotted as coloured lines with 95% credible intervals for RMSD (A), Rg (B), chain interface A (C), chain interface B (D) and chain interface C (E) distances for each trimer type. Raw data are plotted in grey for comparison, as separate replicate time series.

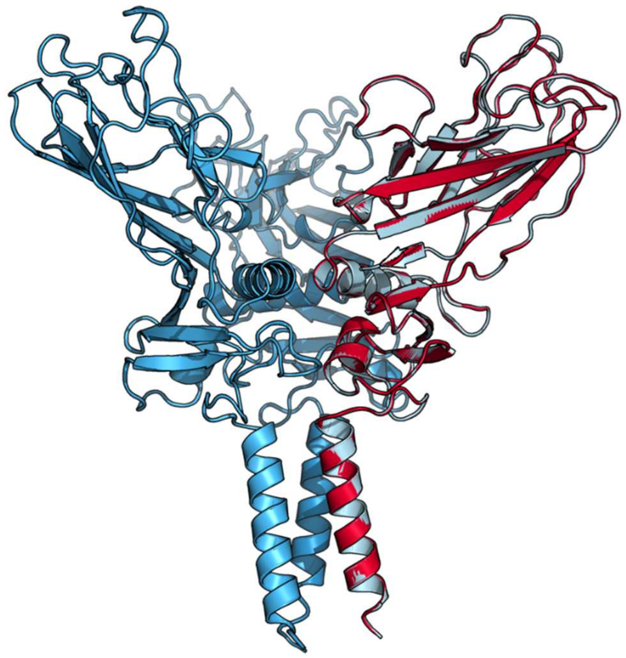

Figure S3: Comparison of the crystal structure of the homotrimeric type I collagen C-propeptide and the homology model of the heterotrimeric C-propeptide (SWISS-MODEL). The two structures are overlaid; in the heterotrimer the α2(I) chain is shown in red, in the homotrimer the corresponding chain is shown in light blue.

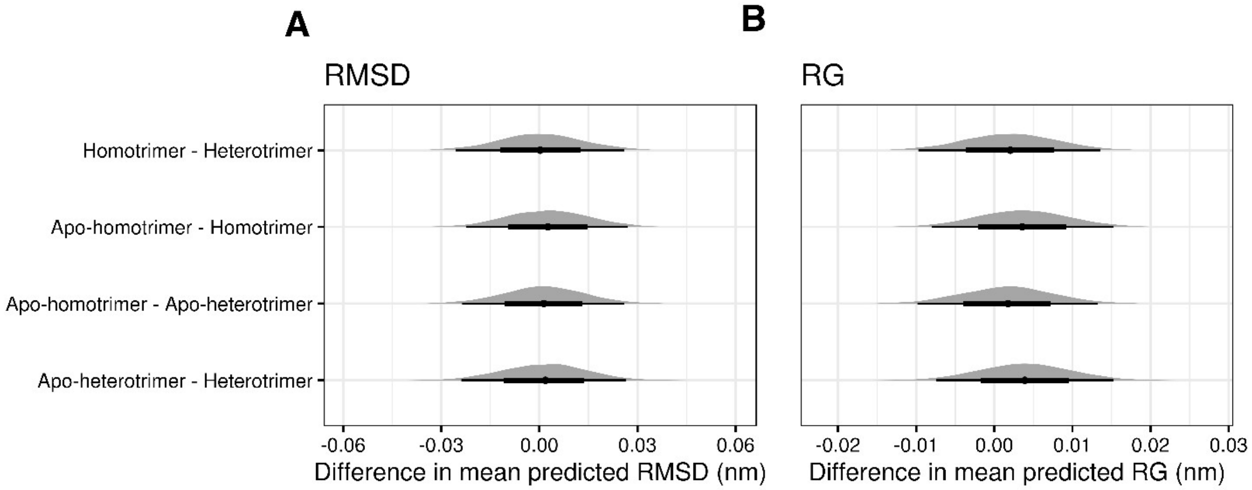

Figure S4: Posterior prediction contrasts between trimer types. Distributions of contrasted posterior predictions of RMSD (A) and Rg (B) independent of time, are plotted as kernel density estimation curves, alongside point intervals indicating the median, 66% and 95% credible intervals of the differences.

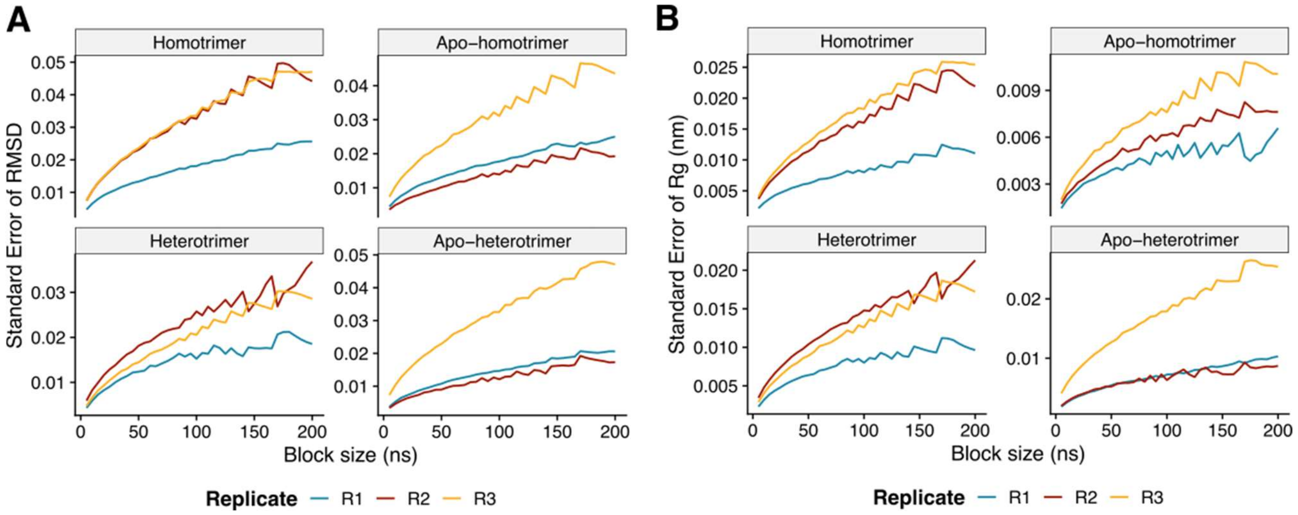

Figure S5: Block Error Analysis: Standard Error (SE) vs Block Size. The SE of the mean RMSD (A) and Rg (B) was evaluated as a function of block size for each replicate trajectory of the holo-homotrimer, apo-homotrimer, holo-heterotrimer, and apo-heterotrimer systems. The coloured lines represent individual replicates. For each trimer type, SE estimates stabilised at block sizes between 40–60 ns, after which variability in SE increased substantially. A block size of 50 ns was therefore selected for subsequent block averaging and summaries of equilibrium simulations.

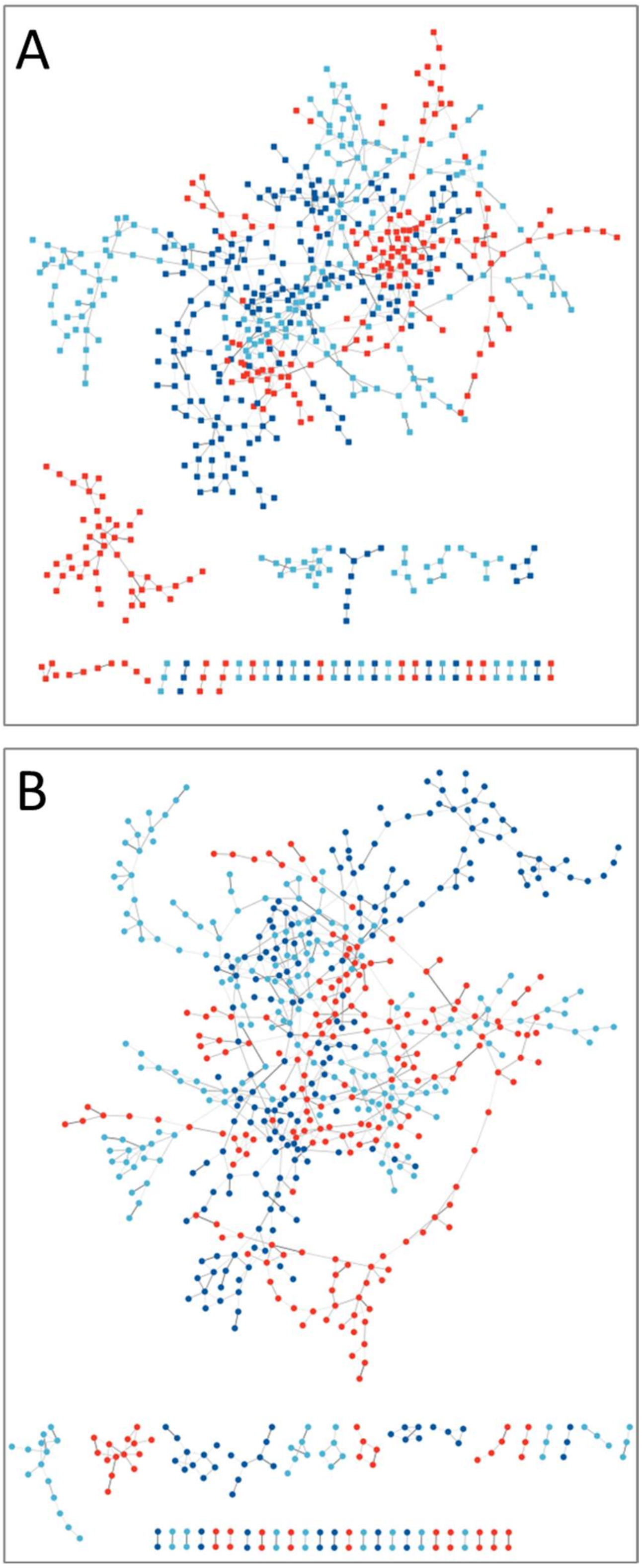

Figure S6: Cytoscape chimera full hydrogen bonding networks. A: Heterotrimer. B: Homotrimer. Red denotes the α2(I) chain for the heterotrimer (A) and the α1 B chain for the homotrimer (B). Blues represent the other α1 chains in each trimer (chains A and C).

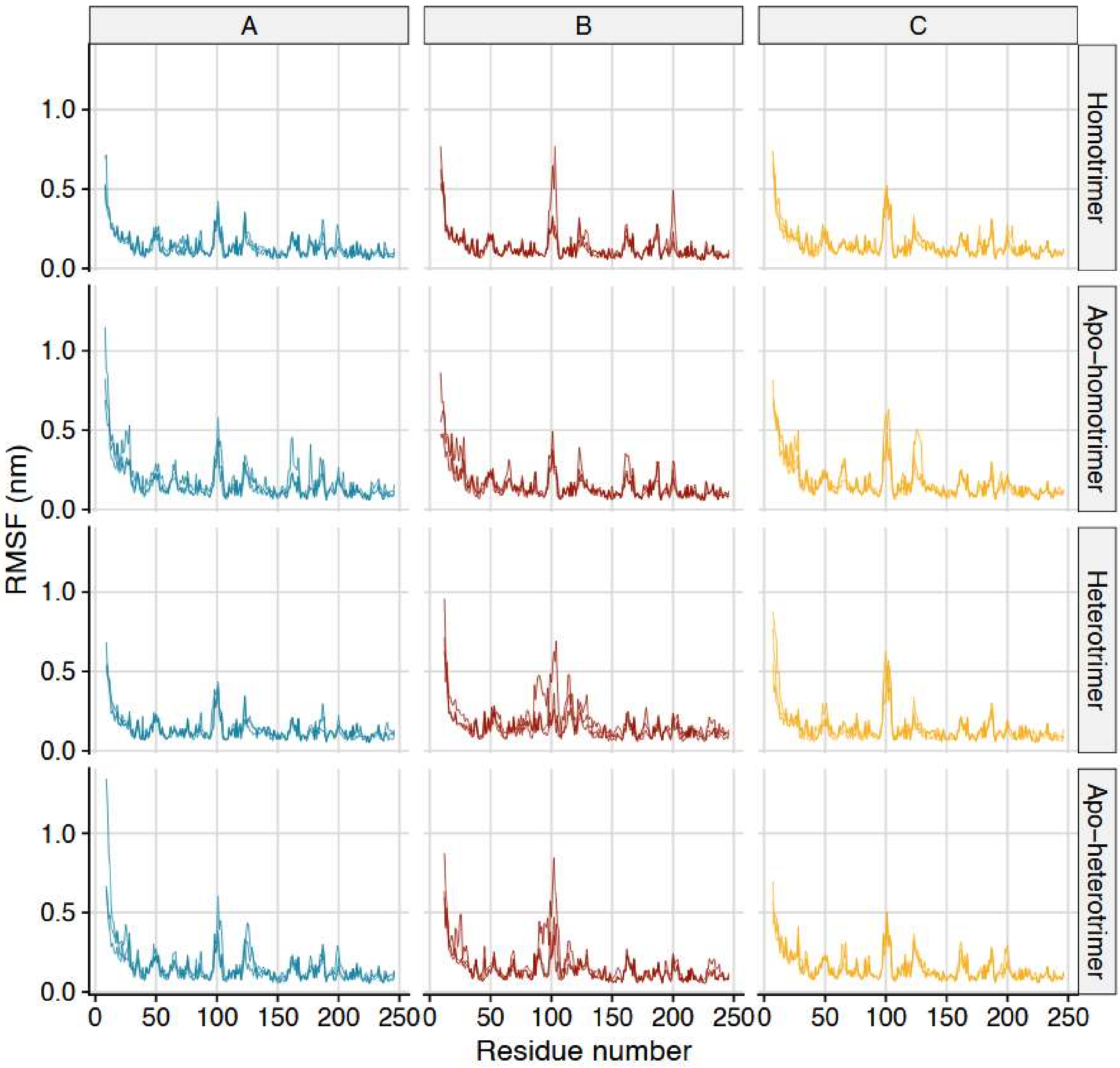

Figure S7: Backbone RMSF (nm) per trimer and chain. Each panel represents a different trimer and chain combination. There are three traces per panel, representing each replicate. The traces are coloured by chain. Each trimer has three chains: chains A, B and C. In the homotrimer all three chains are α1(I). In the heterotrimer chains A and C are α1(I) and chain B is α2(I). Chain A is shown in blue, chain B is shown in red and chain C is shown in yellow.

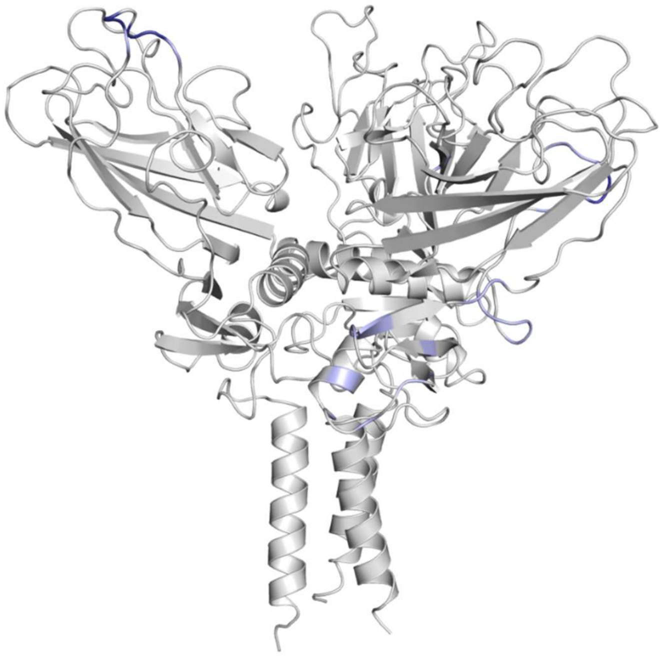

Figure S8: Residues that were stabilised by calcium depletion in the heterotrimer (shown in light blue: ΔRMSF < -0.05 or dark blue: ΔRMSF < -0.1). Most were confined to the exterior of the α2(I) chain.

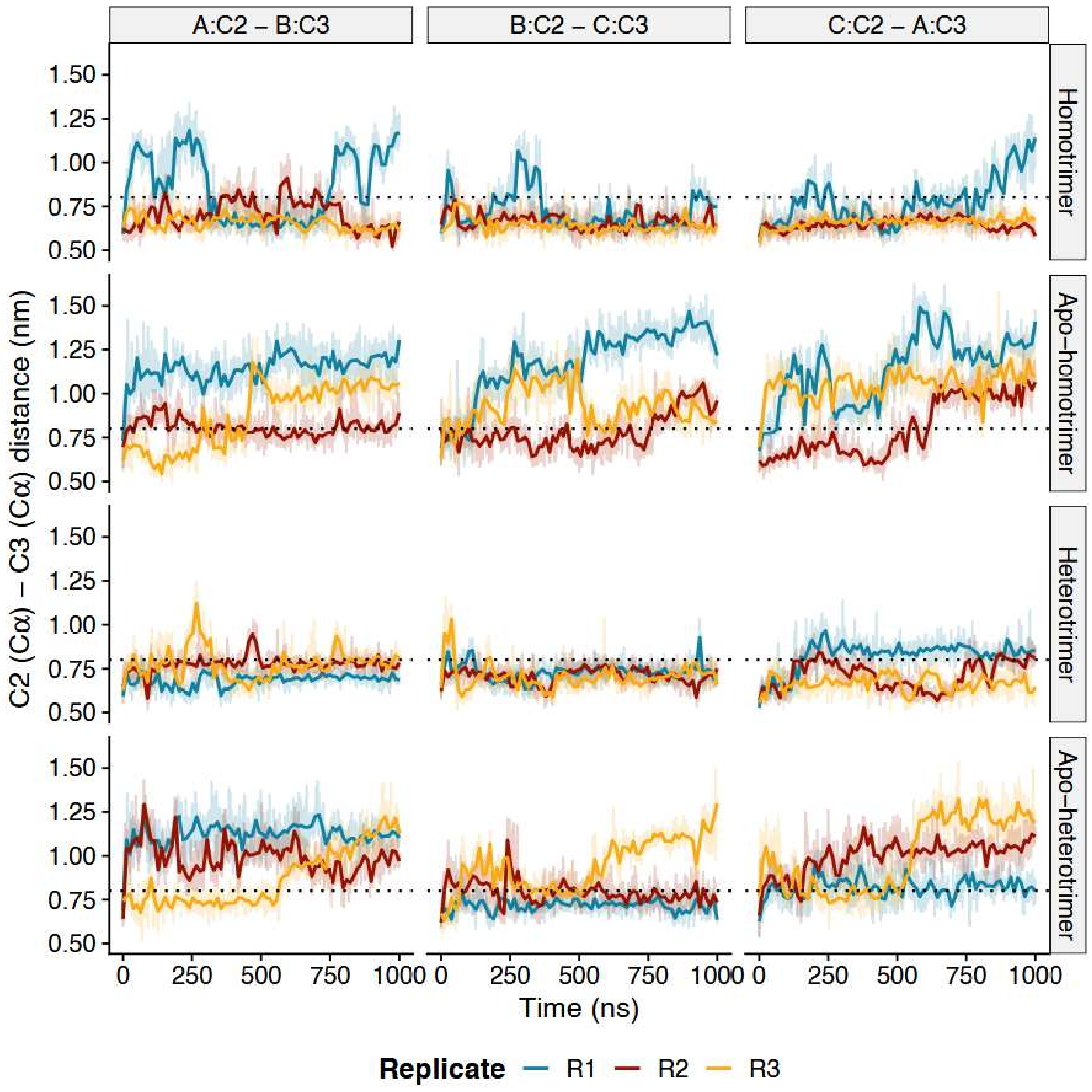

Figure S9: C2-C3 Cα distances per trimer and chain interface. Each panel shows a combination of trimer and chain interface, with distances between C2-C3 alpha carbons displayed as a time series, with each coloured trace representing a different simulation replica. The thick lines show the moving average of 20 ns, while the thinner lines show the distance every 1 ns. The dotted black line is at 0.8 nm, beyond which disulphide bonds are unlikely to form.

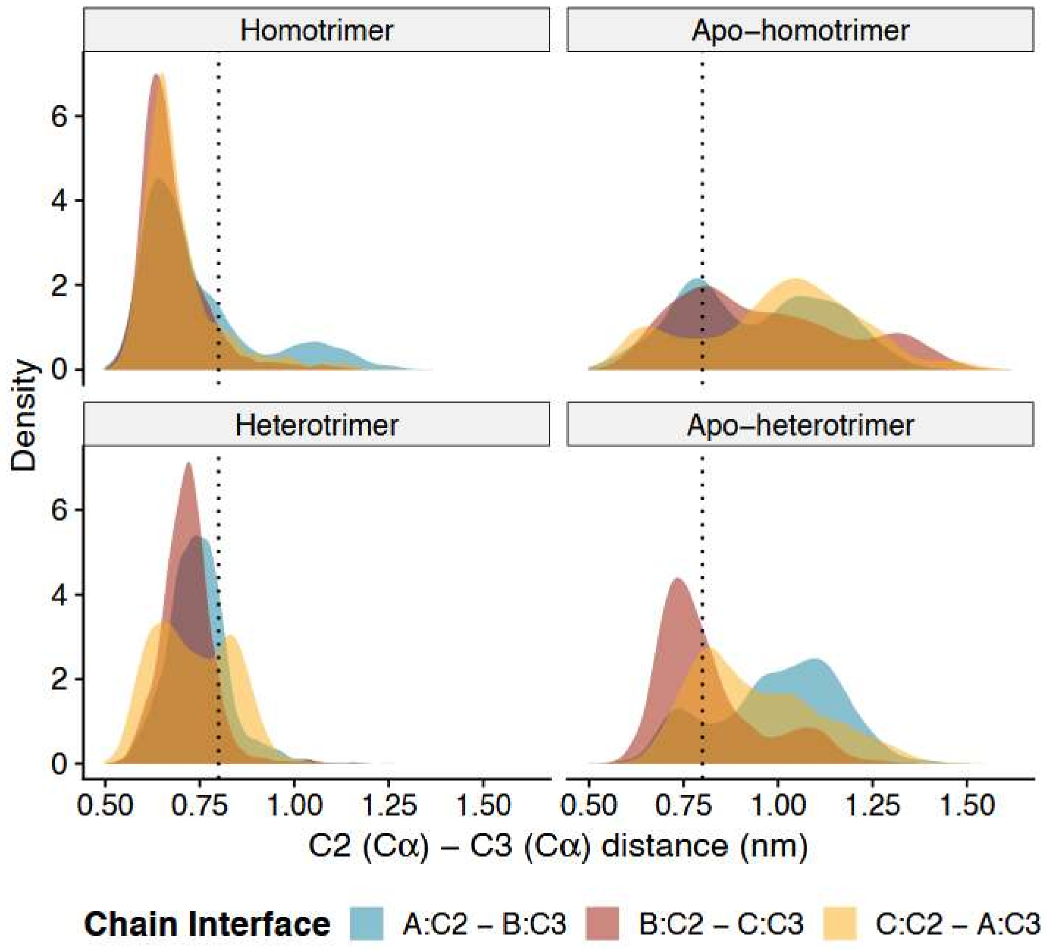

Figure S10: Distribution of C2-C3 Cα distances. The distribution of C2-C3 Cα distances is shown using a kernel density estimate (KDE) function with combined data of all three replicas. Each panel represents a different trimer. The dotted black line is at 0.8 nm, beyond which disulphide bonds are unlikely to form.

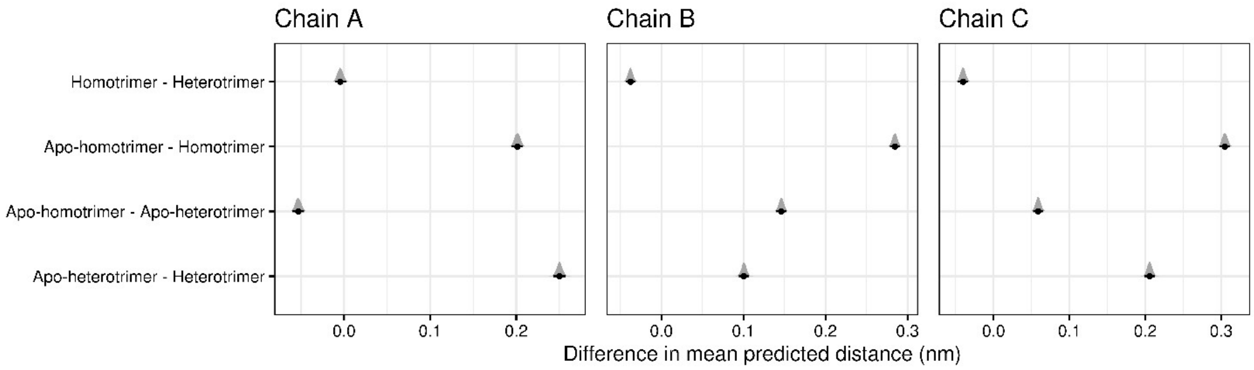

Figure S11: Posterior prediction contrasts between trimer types. Distributions of contrasted posterior predictions of chain interface distances, independent of time, are plotted as kernel density estimation curves, alongside point intervals indicating the median, 66% and 95% credible intervals of the differences.

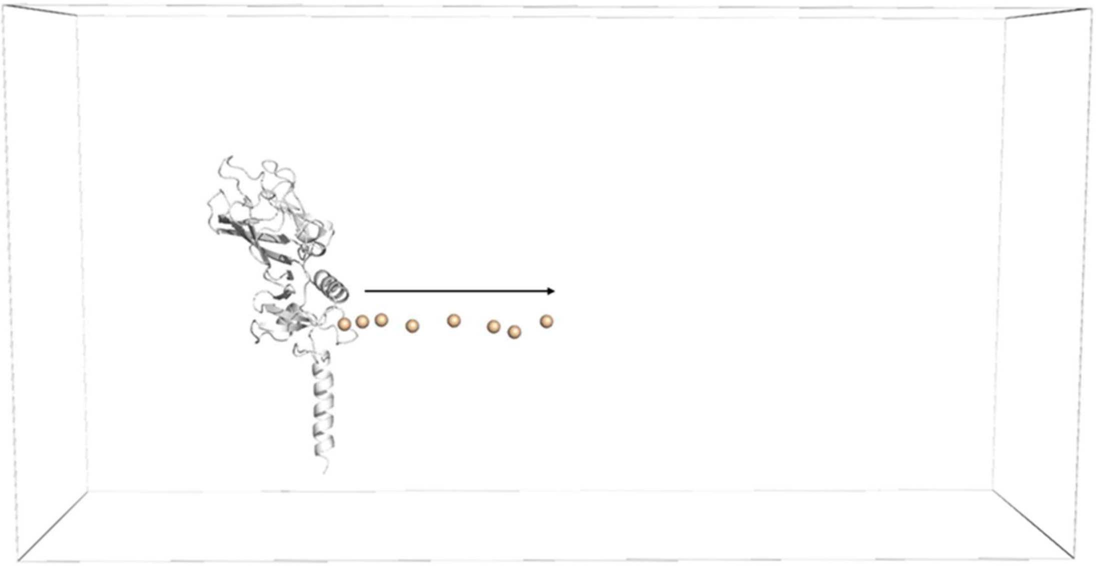

Figure S12: Centre of mass pulling simulation for a calcium ion extracted from its binding site in a monomer by steered molecular dynamics. An α1(I) monomer is shown in white and the calcium ion as a wheat-coloured sphere. The box defines the periodic boundary conditions. Multiple frames of the calcium moving away are shown along the arrow, which was the direction of the pull force.

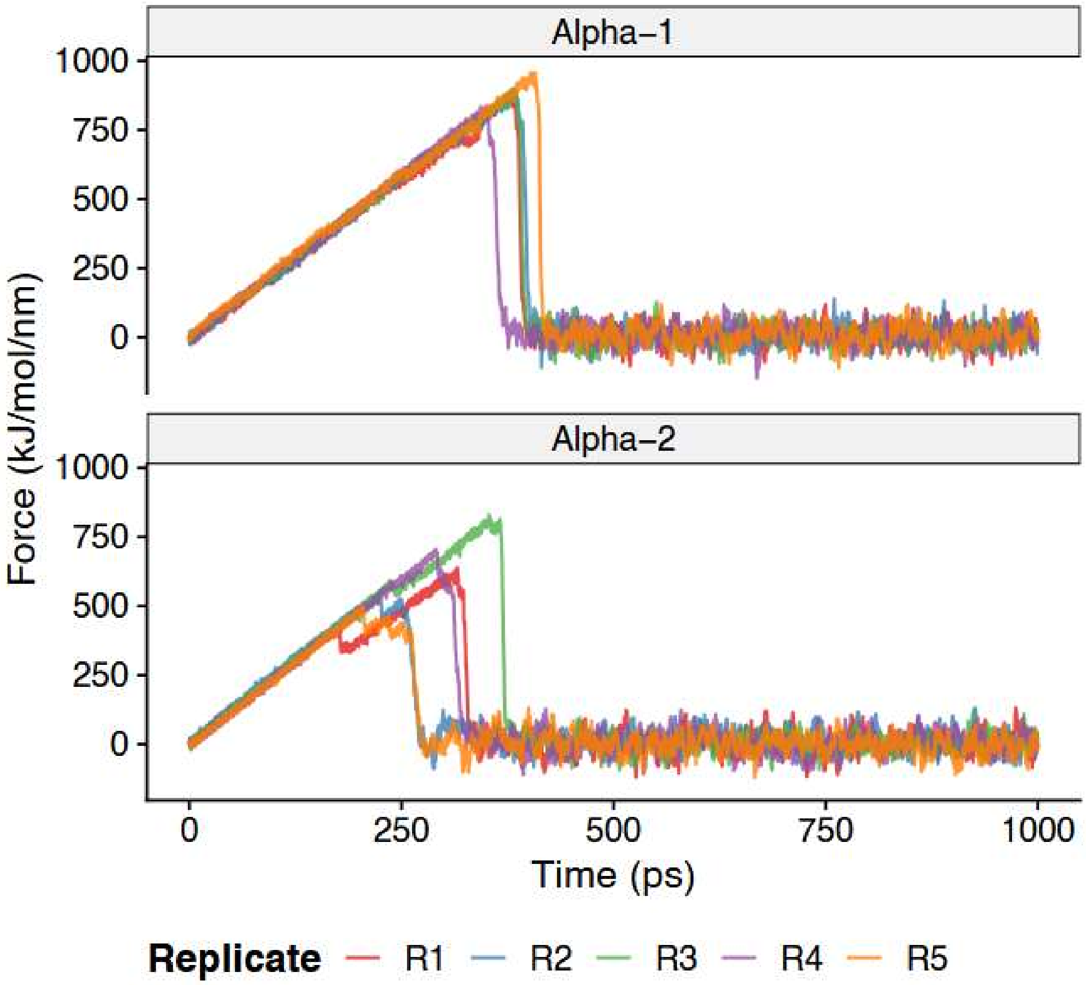

Figure S13: Pulling force per replicate for COM pulling simulations of calcium ion detaching from α1(I) or α2(I) chain. Each panel shows a different chain. The replicates (denoted ‘R1’, ‘R2’, etc.) are shown as individual traces.

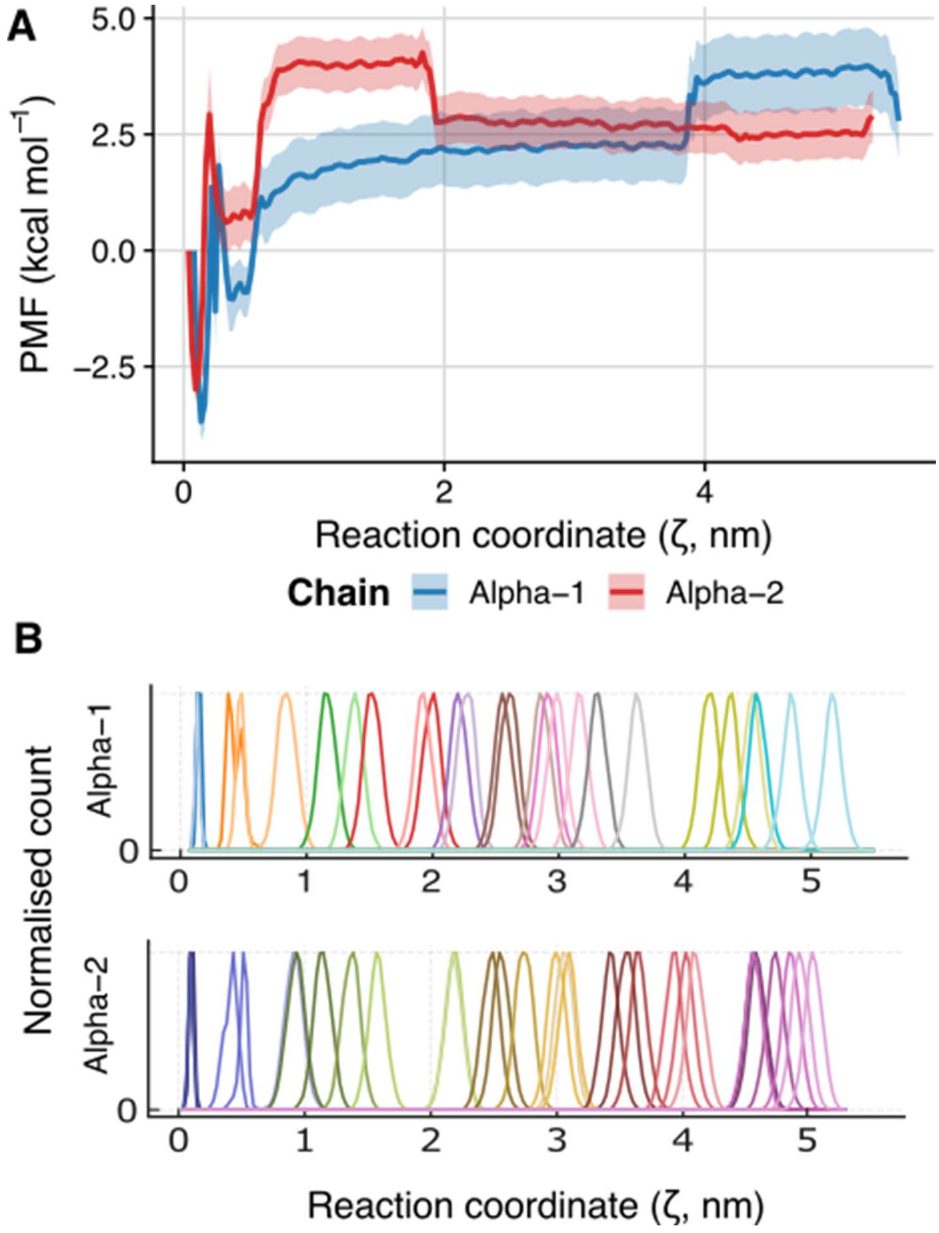

Figure S14: PMFs and umbrella sampling histograms for Ca^{2+} dissociation from the α1(I) and α2(I) chain, obtained via weighted histogram analysis (WHAM). A) The PMF is shown as a solid line for the α1(I) (blue) and α2(I) chains (red), the shaded regions denote the bootstrapped ± 1 SD uncertainties (100 resamples).. The bootstrapped SD bands are narrow (< 0.5 kcal mol^{-1}) in the bound region and widen in the unbound region. However, differences between the two were modest. The deeper α1(I) well could suggest lower bound-state energy. Consistently, t-RAMD yields ~2x longer residence times and steered molecular dynamics shows higher rupture forces for α1(I), implying additional pathway-dependent hidden barriers. These could arise from coordination and local gating, that are not fully captured by the 1D reaction coordinate. B) Normalised umbrella sampling histograms for the α1(I) and α2(I) windows, respectively, illustrating overlap along the reaction co-ordinate.

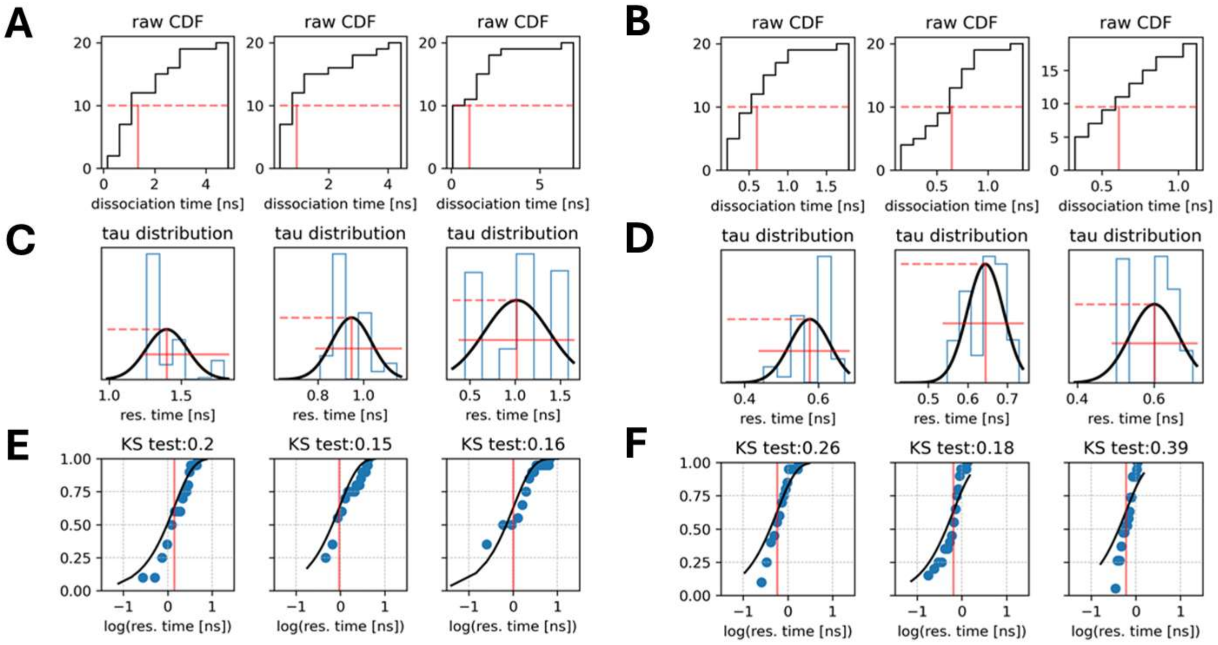

Figure S15: τRAMD statistical analysis for the α1(I) and α2(I) chains. A, B: Analysis of the time at which 50% of the trajectories had dissociated for the α1(I) (A) and α2(I) chain (B). C, D: Fit of a normal distribution to the data for the α1(I) (C) and α2(I) chain (D). E, F: Kolmogorov–Smirnov test results for the for the α1(I) (E) and α2(I) chain (F). The line is the Poisson cumulative distribution function and the blue dots the cumulative density function (CDF).

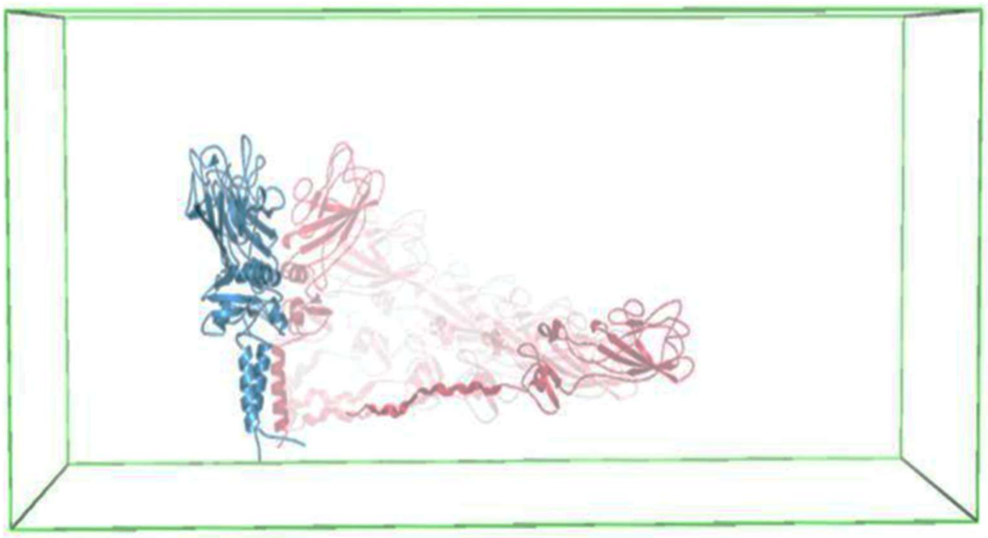

Figure S16: Sample unbinding pathway for the heterotrimer. An α2(I) chain is dissociating from two α1(I) chains along the collective variable (z-axis). The end configuration is shown as the darkest shade of red; the earliest configuration is shown as the next darkest shade. The α1(I) chains are shown in blue.

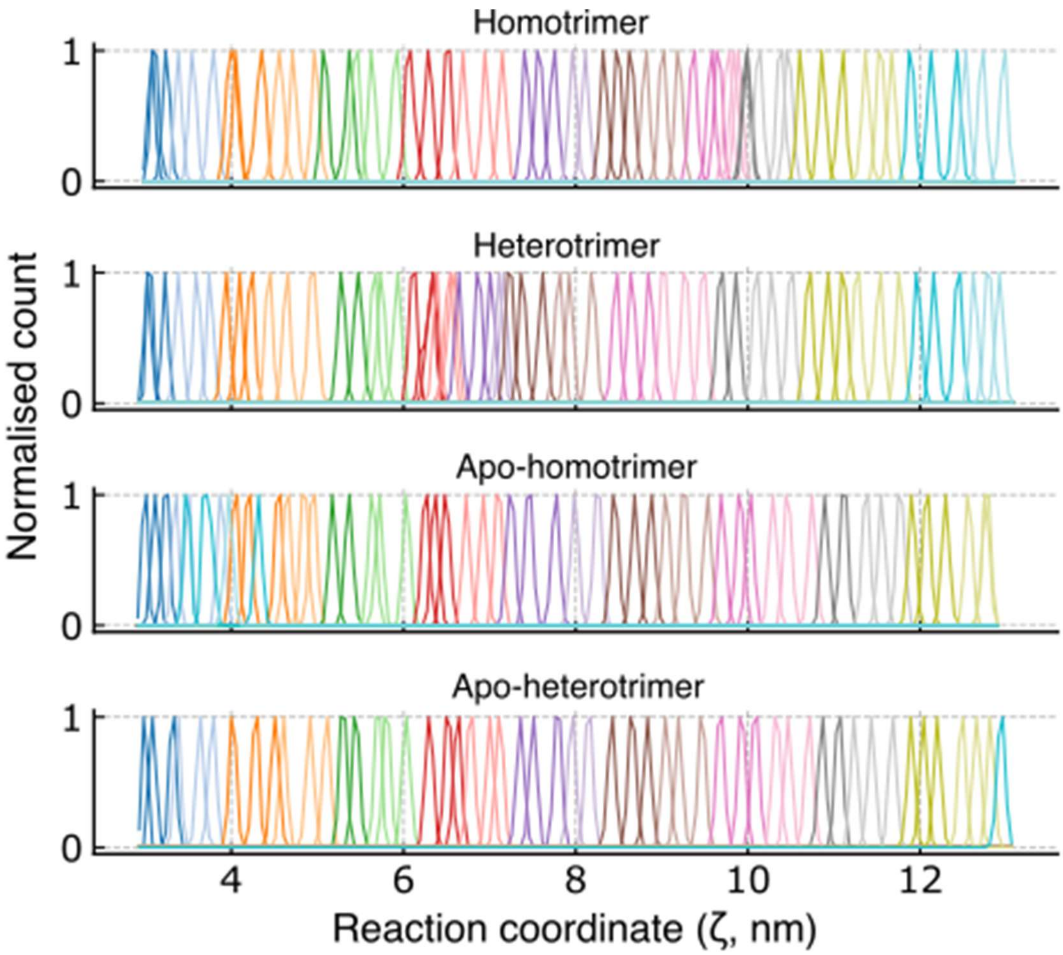

Figure S17: Histograms of the sampling windows for trimer pulling simulations. Histograms show the distribution of umbrella sampling windows used to construct the potential of mean force (PMF) profiles in Figure 10B. The reaction coordinate (ζ, nm) corresponds to the separation of the α2(I) chain (or the corresponding α1(I) chain in homotrimers) from the remaining two chains along a one-dimensional pulling pathway. Each coloured bin represents an individual umbrella sampling window, and the degree of overlap between adjacent windows indicates adequate sampling of configurational space for WHAM analysis. The trimers are a homotrimer, heterotrimer, apo-homotrimer and apo-heterotrimer respectively.
